# Canalization and Control in Automata Networks: Body Segmentation in *Drosophila melanogaster*

**DOI:** 10.1371/journal.pone.0055946

**Published:** 2013-03-08

**Authors:** Manuel Marques-Pita, Luis M. Rocha

**Affiliations:** 1 Instituto Gulbenkian de Ciência, Oeiras, Portugal; 2 Indiana University, Bloomington, Indiana, United States of America; Northwestern University, United States of America

## Abstract

We present schema redescription as a methodology to characterize canalization in automata networks used to model biochemical regulation and signalling. In our formulation, canalization becomes synonymous with redundancy present in the logic of automata. This results in straightforward measures to quantify canalization in an automaton (micro-level), which is in turn integrated into a highly scalable framework to characterize the collective dynamics of large-scale automata networks (macro-level). This way, our approach provides a method to link micro- to macro-level dynamics – a crux of complexity. Several new results ensue from this methodology: uncovering of dynamical modularity (modules in the dynamics rather than in the structure of networks), identification of minimal conditions and critical nodes to control the convergence to attractors, simulation of dynamical behaviour from incomplete information about initial conditions, and measures of macro-level canalization and robustness to perturbations. We exemplify our methodology with a well-known model of the intra- and inter cellular genetic regulation of body segmentation in *Drosophila melanogaster*. We use this model to show that our analysis does not contradict any previous findings. But we also obtain new knowledge about its behaviour: a better understanding of the size of its wild-type attractor basin (larger than previously thought), the identification of novel minimal conditions and critical nodes that control wild-type behaviour, and the resilience of these to stochastic interventions. Our methodology is applicable to any complex network that can be modelled using automata, but we focus on biochemical regulation and signalling, towards a better understanding of the (decentralized) control that orchestrates cellular activity – with the ultimate goal of explaining how do cells and tissues ‘compute’.

## Introduction and Background

The notion of *canalization* was proposed by Conrad Waddington [1] to explain why, under genetic and environmental perturbations, a wild-type phenotype is less variable in appearance than most mutant phenotypes during development. Waddington's fundamental hypothesis was that the robustness of wild-type phenotypes is the result of a *buffering of the developmental process*. This led Waddington to develop the well-known concept of *epigenetic landscape* [2], [3], where cellular phenotypes are seen, metaphorically, as marbles rolling down a sloped and ridged landscape as the result of interactions amongst genes and epigenetic factors. The marbles ultimately settle in one of the valleys, each corresponding to a stable configuration that can be reached via the dynamics of the interaction network. In this view, genetic and epigenetic perturbations can only have a significant developmental effect if they force the natural path of the marbles over the ridges of the epigenetic landscape, thus making them settle in a different valley or stable configuration.

Canalization, understood as the buffering of genetic and epigenetic perturbations leading to the stability of phenotypic traits, has re-emerged recently as a topic of interest in computational and systems biology [4]–[10]. However, canalization is an emergent phenomenon because we can consider the stability of a phenotypic trait both at the micro-level of biochemical interactions, or at the macro-level of phenotypic behaviour. The complex interaction between micro- and macro-level thus makes canalization difficult to study in biological organisms – but the field of complex systems has led to progress in our understanding of this concept. For instance, Conrad [3] provided a still-relevant treatment of evolvability [11] by analysing the tradeoff between genetic (micro-level) instability and phenotypic (macro-level) stability. This led to the concept of *extra-dimensional bypass*, whereby most genetic perturbations are buffered to allow the phenotype to be robust to most physiological perturbations, but a few genetic perturbations (e.g. the addition of novel genetic information) provide occasional instability necessary for evolution. Conrad highlighted three (micro-level) features of the organization of living systems that allows them to satisfy this tradeoff: *modularity* (or compartmentalization), *component redundancy*, and *multiple weak interactions*. The latter two features are both a form of redundancy, the first considering the redundancy of components and the second considering the redundancy of interactions or linkages. Perhaps because micro-level redundancy has been posited as one of the main mechanisms to obtain macro-level robustness, the term canalization has also been used – especially in discrete mathematics – to characterize redundant properties of automata functions, particularly when used to model micro-level dynamical interactions in models of genetic regulation and biochemical signalling.

An automaton is typically defined as *canalizing* if there is at least one state of at least one of its inputs that is sufficient to control the automaton's next state (henceforth *transition*), regardless of the states of any other inputs [12]. Clearly, this widely used definition refers to micro-level characteristics of the components of multivariate discrete dynamical systems such as automata networks, and not to canalization as the emergent phenomenon outlined above. Nonetheless, using this definition, it has been shown that (1) canalizing functions are widespread in eukaryotic gene-regulation dynamics [13]; (2) genetic regulatory networks modelled with canalizing automata are always stable [14], [15]; and (3) realistic biological dynamics are naturally observed in networks with scale-free connectivity that contain canalizing functions [16]. These observations suggest that the redundancy captured by this micro-level definition of canalization is a mechanism used to obtain stability and robustness at the macro-level of phenotypic traits.

Since the proportion of such ‘strictly’ canalizing functions drops abruptly with the number of inputs (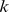) [17], it was at first assumed that (micro-level) canalization does not play a prominent role in stabilizing complex dynamics of gene regulatory networks. However, when the concept of canalization is extended to include *partially canalizing* functions, where subsets of inputs can control the automaton's transition, the proportion of available canalizing automata increases dramatically even for automata with many inputs [18]. Furthermore, partial canalization has been shown to contribute to network stability, without a detrimental effect on ‘evolvability’ [18]. Reichhardt and Bassler, point out that, even though strictly canalizing functions clearly contribute to network stability, they can also have a detrimental effect on the ability of networks to adapt to changing conditions [18] – echoing Conrad's tradeoff outlined above. This led them to consider the wider class of partially canalizing functions that confer stable network dynamics, while improving adaptability. A function of this class may ignore one or more of its inputs given the states of others, but is not required to have a single canalizing input. For example, if a particular input is *on*, the states of the remaining inputs are irrelevant, but if that same input is *off*, then the state of a subset of its other inputs is required to determine the function's transition. In scenarios where two or more inputs are needed to determine the transition, the needed inputs are said to be *collectively canalizing*.

Reichhardt and Bassler [18] have shown that the more general class of partially canalizing functions dominates the space of Boolean functions for any number of inputs 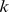. Indeed, for any value of 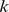, there are only two *non-canalizing functions* that always depend on the states of all inputs. Other classes of canalizing functions have been considered, such as *nested canalizing* functions [14], *Post classes* [19] and *chain functions* [20]. All these classes of functions characterize situations of input redundancy in automata. In other words, micro-level canalization is understood as a form of redundancy, whereby a subset of input states is sufficient to guarantee transition, and therefore its complement subset of input states is redundant. This does not mean that redundancy is necessarily the sole – or even most basic – mechanism to explain canalization at the macro-level. But the evidence we reviewed above, at the very least, strongly suggests that micro-level redundancy is a key mechanism to achieve macro-level canalization. Other mechanisms are surely at play, such as the topological properties of the networks of micro-level interactions. Certainly, modularity, as suggested by Conrad, plays a role in the robustness of complex systems and has rightly received much attention recently [21]. While we show below that some types of modularity can derive from micro-level redundancy, other mechanisms to achieve modularity are well-known [21].

Here, we explore partial canalization, as proposed by Reichhardt and Bassler [18], to uncover the loci of control in complex automata networks, particularly those used as models of genetic regulation and signalling. Moreover, we extend this notion to consider not only (micro-level) canalization in terms of input redundancy, but also in terms of input-permutation redundancy to account for the situations in which swapping the states of (a subset) of inputs has no effect on an automaton's transition. From this point forward, when we use the term *canalization* we mean it in the micro-level sense used in the (discrete dynamical systems) literature to characterize redundancy in automata functions. Nonetheless, we show that the quantification of such micro-level redundancy uncovers important details of macro-level dynamics in automata networks used to model biochemical regulation. This allows us to better study how robustness and control of phenotypic traits arises in such systems, thus moving us towards understanding canalization in the wider sense proposed by Waddington. Before describing our methodology, we introduce necessary concepts and notations pertaining to Boolean automata and networks, as well as the segment polarity gene-regulation network in *Drosophila melanogaster*, an automata model we use to exemplify our approach.

### Boolean Networks

**Figure 1:**
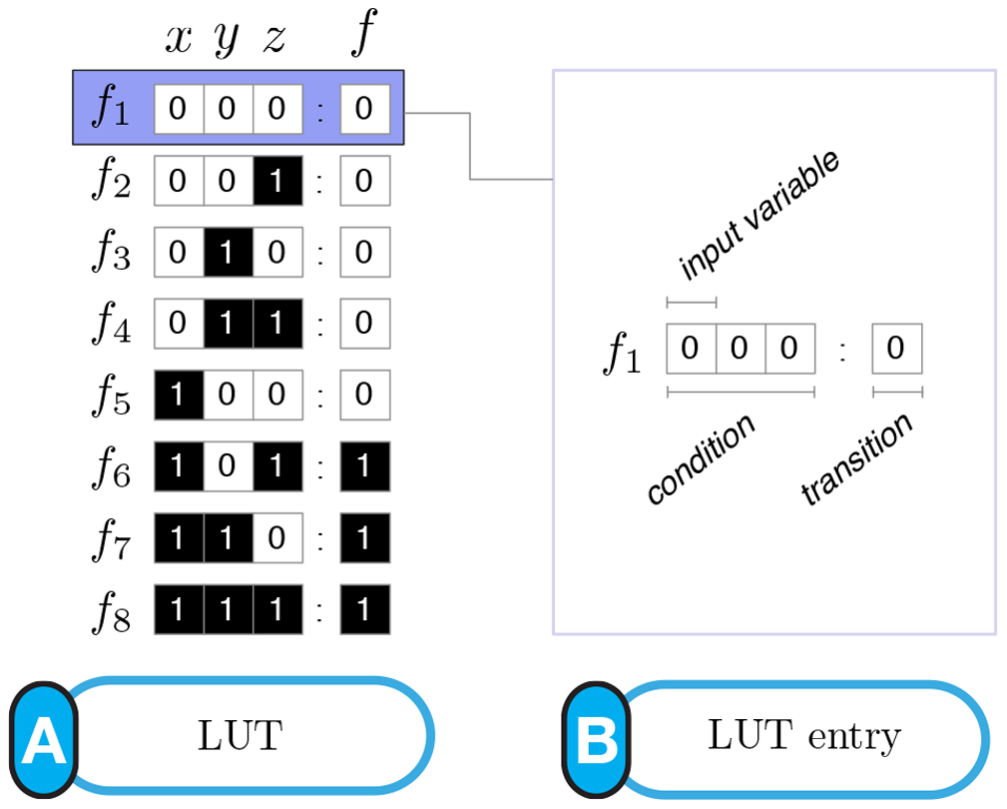
(A) LUT for Boolean automaton 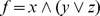
 **and (B) components of a single LUT entry.**

This type of discrete dynamical system was introduced to build qualitative models of genetic regulation, very amenable to large-scale statistical analysis [22] – see [23] for comprehensive review. A *Boolean automaton* is a binary variable, 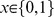, where state 0 is interpreted as *false* (*off* or *unexpressed*), and state 1 as *true* (*on* or *expressed*). The states of 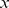 are updated in discrete time-steps, 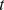, according to a *Boolean state-transition function* of 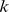 inputs: 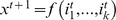. Therefore 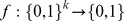. Such a function can be defined by a *Boolean logic formula* or by a *look-up (truth) table* (LUT) with 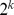 entries. An example of the former is 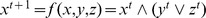, or its more convenient shorthand representation 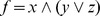, which is a Boolean function of 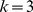 input binary variables 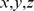, possibly the states of other automata; 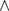, 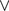 and 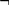 denote logical conjunction, disjunction, and negation respectively. The LUT for this function is shown in Figure 1. Each LUT entry of an automaton 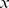, 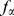, is defined by (1) a specific *condition*, which is a conjunction of 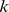 inputs represented as a unique 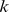-tuple of input-variable (Boolean) states, and (2) the automaton's *next state* (transition) 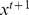, given the condition (see Figure 1). We denote the entire state transition function of an automaton 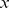 in its LUT representation as 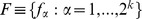.

A *Boolean Network* (BN) is a graph 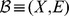, where 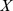 is a set of 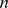 Boolean automata *nodes* 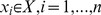, and 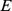 is a set of directed edges 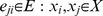. If 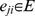, it means that automaton 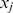 is an input to automaton 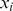, as computed by 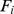. 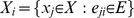 denotes the set of input automata of 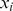. Its cardinality, 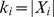, is the *in-degree* of node 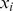, which determines the size of its LUT, 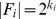. We refer to each entry of 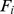 as 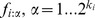. The *input nodes* of 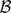 are nodes whose state does not depend on the states of other nodes in 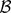. The state of *output nodes* is determined by the states of other nodes in the network, but they are not an input to any other node. Finally, the state of *inner nodes* depends on the state of other nodes and affect the state of other nodes in 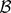. At any given time 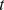, 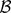 is in a specific *configuration* of node states, 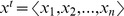. We use the terms *state* for individual automata 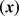 and *configuration* 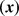 for the collection of states of the set of automata of 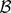, i.e. the collective network state.

Starting from an initial configuration, 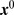, a BN updates its nodes with a *synchronous* or *asynchronous* policy. The *dynamics* of 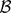 is thus defined by the temporal sequence of configurations that ensue, and there are 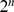 possible configurations. The transitions between configurations can be represented as a *state-transition graph*, 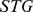, where each vertex is a configuration, and each directed edge denotes a transition from 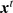 to 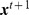. The STG of 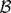 thus encodes the network's entire *dynamical landscape*. Under synchronous updating, configurations that repeat, such that 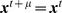, are known as *attractors*; *fixed point* when 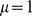, and *limit cycle* – with period 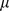 – when 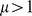, respectively. The disconnected subgraphs of a STG leading to an attractor are known as *basins of attraction*. In contrast, under asynchronous updating, there are alternative configuration transitions that depend on the order in which nodes update their state. Therefore, the same initial configuration can converge to distinct attractors with some probability [24], [25]. A BN 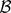 has a finite number 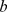 of attractors; each denoted by 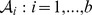. When the updating scheme is known, every configuration 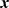 is in the basin of attraction of some specific attractor 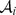. That is, the dynamic trajectory of 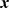 converges to 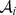. We denote such a dynamical trajectory by 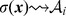. If the updating scheme is stochastic, the relationship between configurations and attractors can be specified as the conditional probability 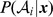.

### The Segment Polarity Network

The methodology introduced in this paper will be exemplified using the well-studied Boolean model of the segment polarity network in *Drosophila melanogaster* [26]. During the early ontogenesis of the fruit fly, like in every arthropod's development, there is body segmentation [27], [28]. The specification of adult cell types in each of these segments is controlled by a hierarchy of around forty genes. While the effect of most of the genes in the hierarchy is only transient, a subset of *segment polarity genes* remains expressed during the life of the fruit fly [29]. The dynamics of the segment polarity network was originally modelled using a system of non-linear ordinary differential equations (ODEs) [30], [31]. This model suggested that the regulatory network of segment polarity genes is a module largely controlled by external inputs that is robust to changes to its internal kinetic parameters. On that basis, Albert and Othmer later proposed a simpler discrete BN model of the dynamics of the *segment polarity network* (henceforth SPN) [26]. This was the first Boolean model of gene regulation capable of predicting the steady state patterns experimentally observed in wild-type and mutant embryonic development with significant accuracy, and has thus become the quintessential example of the power of the logical approach to modelling of biochemical regulation from qualitative data in the literature. Modelling with ODEs, in contrast, is hindered by the need of substantial quantitative data for parameter estimation [32]–[37].

**Figure 2:**
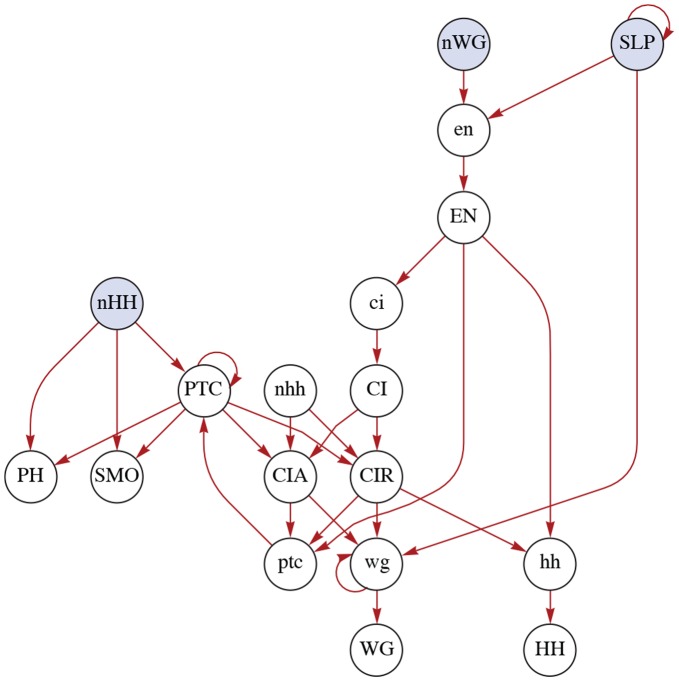
Connectivity graph of the SPN model. The fifteen genes and proteins considered in the SPN model are represented (white nodes). The incoming edges to a node 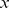 originate in the nodes that are used by 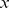 to determine its transition. Shaded nodes represent the spatial signals (states of WG, HH and 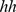 in neighbouring cells). Note that SLP – derived from an upstream intra-cellular signal – is an *input* node to this network. The self-connection it has represents the steady-state assumption: 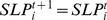. Notice also that this graph represents the fully synchronous version of the model, where modifications concerning PH and SMO have been made (see main text for details).

**Figure 3:**
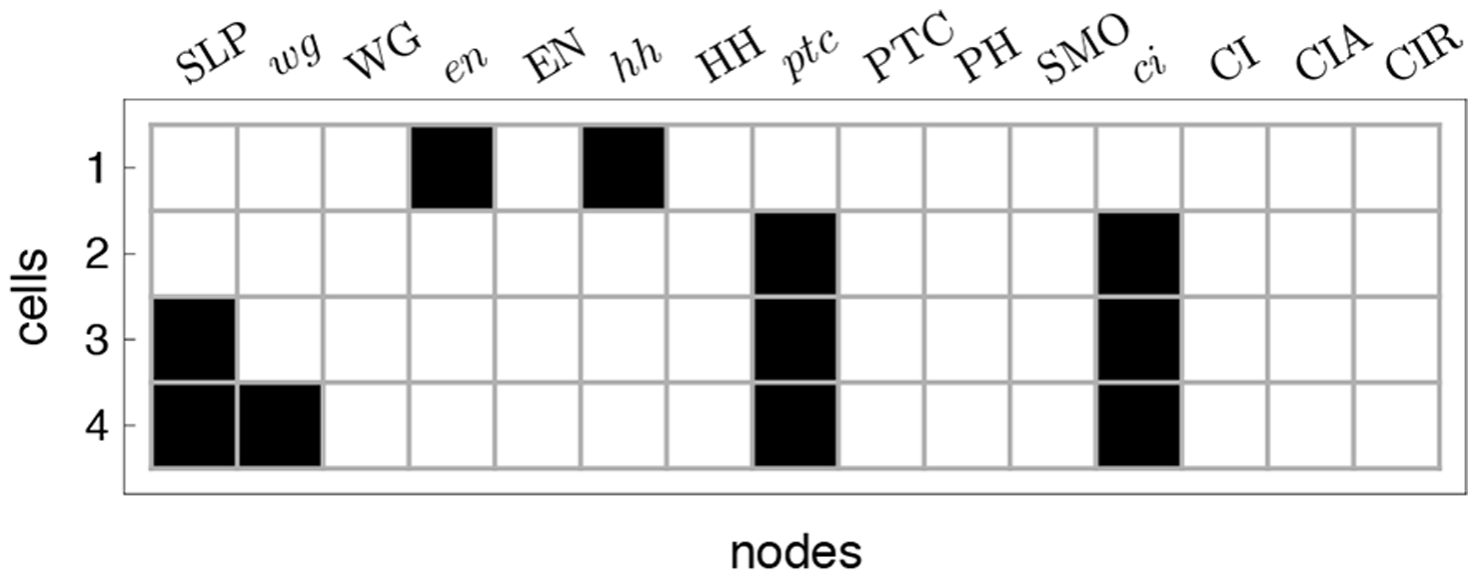
A parasegment in the SPN model. Cells are represented horizontally, where the top (bottom) row is the most anterior (posterior) cell. Each column is a gene, protein or complex – a node in the context of the BN model. The specific pattern shown corresponds to the wild-type initial expression pattern observed at the onset of the segment polarity genes regulatory dynamics (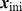); Black/on (white/off) squares represent expressed (not expressed) genes or proteins.

The SPN model comprises fifteen nodes that represent intra-cellular chemical species and the genes *engrailed (en)*; *wingless (wg)*; *hedgehog (hh)*; *patched (ptc)* and *cubitus interruptus (ci)* [29]–[31]. These genes encode a number of proteins such as the transcription factors Engrailed (EN), Cubitus Interruptus (CI), CI Activator (CIA), and CI repressor (CIR); the secreted proteins Wingless (WG) and Hedgehog (HH); and the transmembrane protein Patched (PTC). Other proteins included in the SPN model are Sloppy-Paired (SLP) – the state of which is previously determined by the *pair-rule* gene family that stabilizes its expression before the segment polarity genes – as well as Smoothened (SMO) and the PH complex that forms when HH from neighbouring cells binds to PTC. Figure 2 shows the topology and Table 1 lists the logical rules of the nodes in every cell of the SPN. This model consists of a spatial arrangement of four interconnected cells, a *parasegment*. While the regulatory interactions within each cell are governed by the same network, inter-cellular signalling affects neighbouring cells. That is, regulatory interactions in a given cell depend on the states of WG, 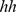 and HH in adjacent cells. Therefore, six additional (inter-cellular) ‘spatial signals’ are included: 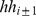, 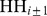 and 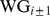, where 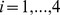 is the cell index in the four-cell parasegment. In a parasegment, the cell with index 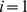 corresponds to its anterior cell and the cell with index 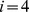 to its posterior cell (see Figure 3). In simulations, the four-cell parasegments assume periodic boundary conditions (i.e. anterior and posterior cells are adjacent to each other). Since each parasegment has 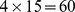 nodes, four of which are in a fixed state (SLP), there are 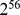 possible configurations – a dynamical landscape too large for exhaustive analysis. Even though the original model was not fully synchronous because 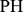 and 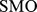 were updated instantaneously at time 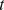, rather than at 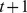, here we use the fully equivalent, synchronous version. Specifically, since 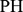 is an output node, synchronizing its transition with the remaining nodes at 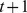 does not impact the model's dynamics. The state of 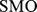 influences the updating of 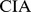 and 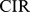; but since the update of 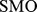 is instantaneous, we can include its state-transition function in the state-transition functions of 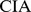 and 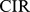 (which update at 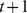) with no change in the dynamics of the model as described in [38].

**Table 1:** Boolean logic formulae representing the state-transition functions for each node in the SPN (four-cell parasegment) model.

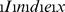	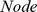	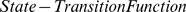
1	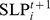	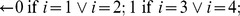
2	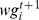	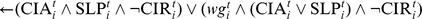
3	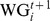	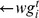
4	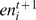	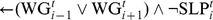
5	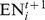	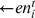
6	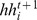	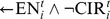
7	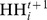	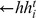
8	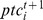	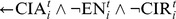
9	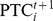	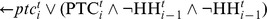
10	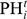	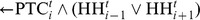
11	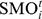	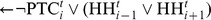
12	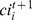	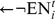
13	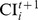	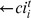
14	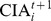	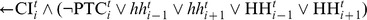
15	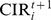	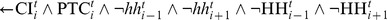

The subscript represents the cell index; the superscript represents time. Note that every node has a numerical index assigned to it, which we use for easy referral throughout the present work. The extra-cellular nodes, 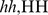 and 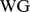 in adjacent cells are indexed as follows: 16 to 21 denote 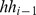, 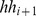, 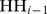, 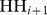, 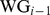 and 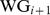 in this order.

**Figure 4:**
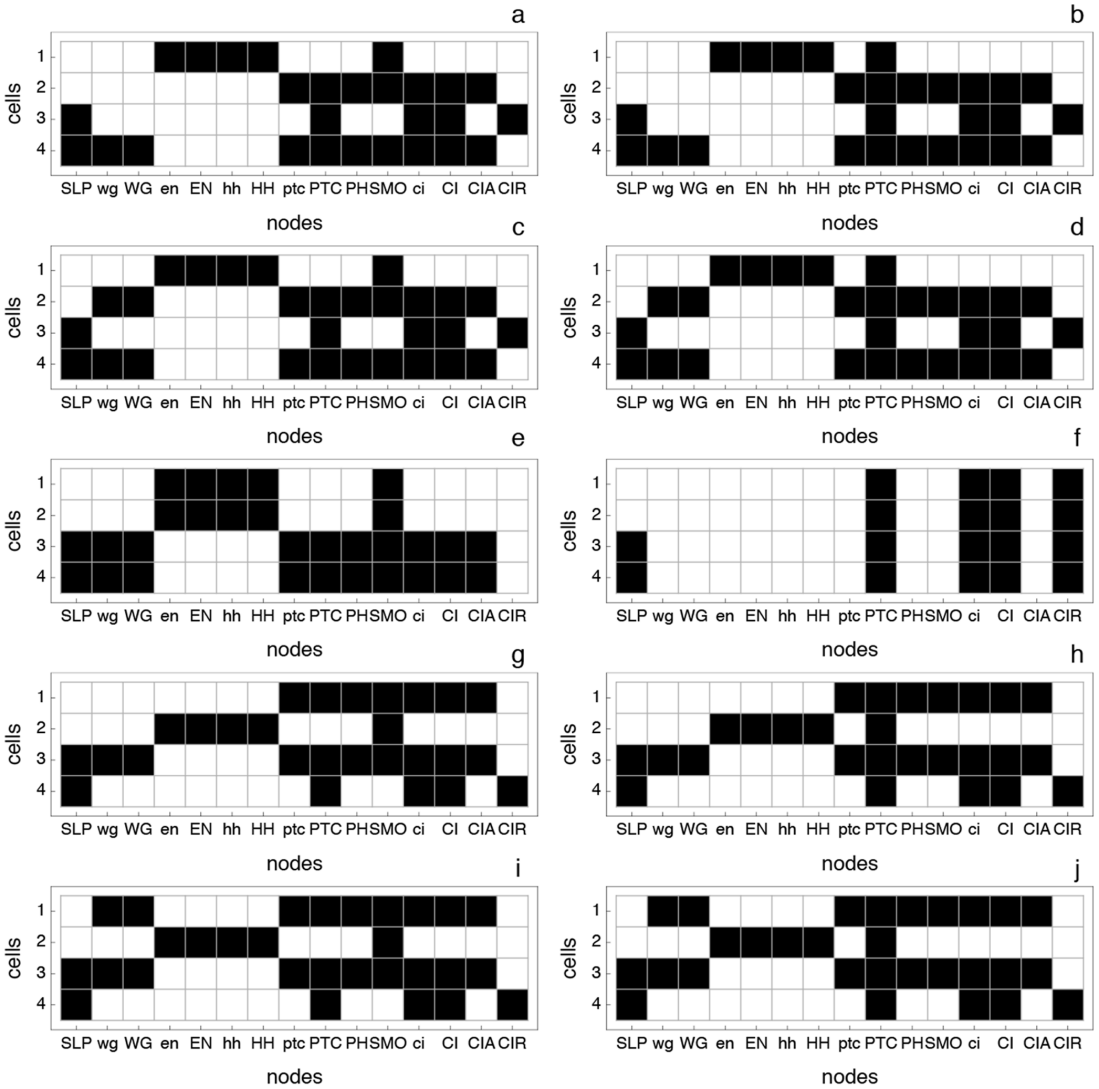
The ten attractors reached by the SPN. These attractors are divided in four groups: wild-type, broad-stripe, no segmentation and ectopic. More specifically: (a) wild-type, (b) variant of (a), (c) wild-type with two 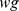 stripes, (d) variant of (c), (e) broad-stripe, (f) no segmentation, (g) ectopic, (h) variant of (g), (i) ectopic with two 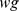 stripes, and (j) variant of (i). The wild-type attractor (a) is referred to as 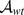 in the results and discussion sections.

The initial configuration (IC) of the SPN, depicted in Figure 3, and which leads to the wild-type expression pattern is known [26]: 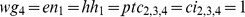 (*on* or expressed). The remaining nodes in every cell of the parasegment are set to 0 (*off*, or not expressed). Overall, the dynamics of the SPN settles to one of ten attractors – usually divided into four qualitatively distinct groups, see Figure 4: (1) wild-type with three extra variations (PTC mutant, double 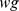 bands, double 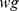 bands PTC mutant); (2) Broad-stripe mutant; (3) No segmentation; and (4) Ectopic (with the same variations as wild-type). Albert and Othmer estimated that the number of configurations that converge to the wild-type attractor is approximately 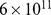 – a very small portion of the total number of possible configurations (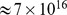) – and that the broad-stripe mutant attractor basin contains about 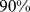 of all possible configurations [26].

The inner and output nodes of each cell in a parasegment – that is, every node except the input node SLP – that has reached a stable configuration (attractor) are always in one of the following five patterns.

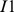
: all nodes are *off* except PTC, 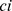, CI and CIR.

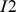
: same as 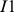 but states of 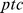, PH, SMO, CIA and CIR are negated.

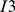
: all nodes are *off* except 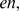 EN, 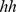, HH and SMO.

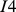
: same as 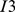 but PTC and SMO are negated.

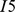
: negation of 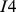, except PTC and CIR remain as in 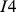.

For example, the wild-type configuration corresponds – from anterior to posterior cell – to the patterns 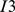, 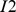, 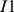 and 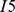. Thus the pattern 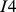 is only seen in mutant expression patterns. The patterns 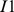 to 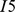 can be seen as attractors of the inner- and output-node dynamics of each cell in a parasegment.

Besides the fact that the SPN is probably the most well-known discrete dynamical system model of biochemical regulation, we chose it to exemplify our methodology because (1) it has been well-validated experimentally, despite the assumption that genes and proteins operate like *on/off* switches with synchronous transitions and (2) the model includes both intra-cellular regulation and inter-cellular signalling in a spatial array of cells. The intra and inter-cellular interactions in the SPN model result in a dynamical landscape that is too large to characterize via an STG, while adding also an extra level of inter-cellular (spatial) regulation. The ability to deal with such multi-level complexity makes our methodology particularly useful. As we show below, we can uncover the signals that control collective information processing in such (spatial and non-spatial) complex dynamics.

## Methodology and Results

### Micro-level Canalization via Schemata

In previous work, we used *schema redescription* to demonstrate that we can understand more about the dynamical behaviour of automata networks by analysing the patterns of *redundancy* present in their (automata) components (micro-level), rather than looking solely at their macro-level or collective behaviour [39]. Here we relate the redundancy removed via schema redescription with the concept of *canalization*, and demonstrate that a characterization of the full canalization present in biochemical networks leads to a better understanding of how cells and collections of cells ‘compute’. Moreover, we show that this leads to a comprehensive characterization of *control* in automata models of biochemical regulation. Let us start by describing the schema redescription methodology. Since a significant number of new concepts and notations are introduced in this, and subsequent sections, a succinct glossary of terms as well as a table with the mathematical notations used is available in Data *S1*.

From the extended view of canalization introduced earlier, it follows that the inputs of a given Boolean automaton do not control its transitions equally. Indeed, substantial redundancy in state-transition functions is expected. Therefore, filtering redundancy out is equivalent to identifying the loci of control in automata. In this section we focus on identifying the loci of control in individual automata by characterizing the canalization present in their transition functions. First, we consider how subsets of inputs in specific state combinations make other inputs *redundant*. Then we propose an additional form of canalization that accounts for *input permutations* that leave a transition unchanged. Later, we use this characterization of canalization and control in individual automata to study networks of automata; this also allows us to analyse robustness and collective computation in these networks.

#### Wildcard schemata and *enputs*

**Figure 5:**
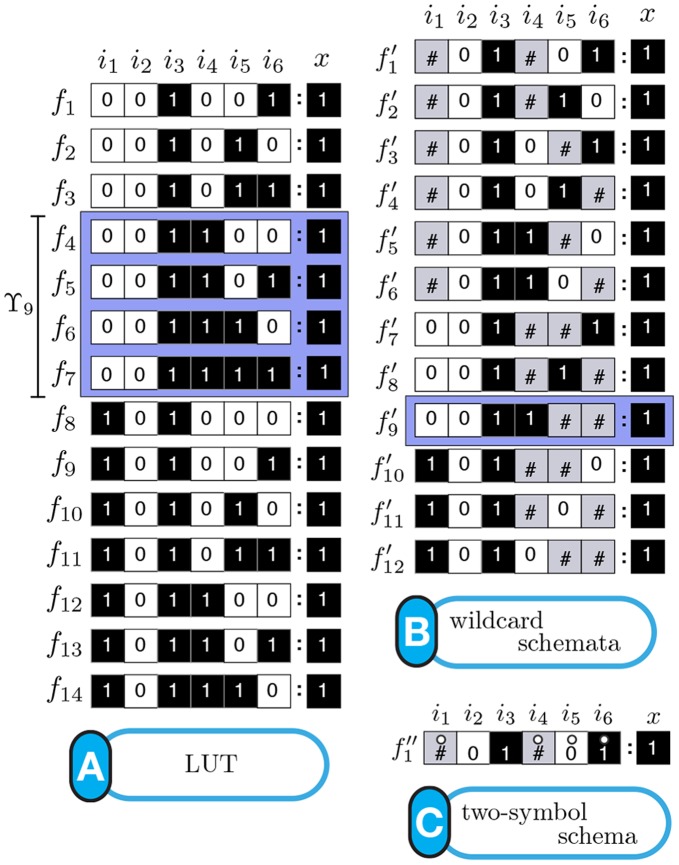
Schema redescription. (A) Subset of LUT entries of an example automaton 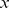 that prescribe state transitions to *on* (1); white (black) states are 0 (1). (B) Wildcard schema redescription; wildcards denoted also by grey states. Schema 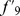 is highlighted to identify the subset of LUT entries 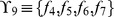 it redescribes. (C) Two-symbol schema redescription, using the additional position-free symbol; the entire set 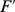 is compressed into a single two-symbol schema: 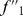. Any permutation of the inputs marked with the position-free symbol in 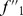 results in a schema in 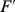. Note that 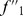 redescribes the entire set of entries with transition to *on* and thus 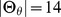. Since there is only one set of marked inputs, the position-free symbol does not require an index. Although this figure depicts only the schemata obtained for the subset of LUT entries of 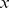 that transition to *on*, entries that do not match any of these schemata transition to *off* (since 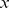 is a Boolean automaton).

Consider the example automaton 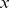 in Figure 5A, where the entire subset of LUT entries in 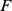 with transitions to *on* is depicted. This portion of entries in 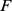 can be *redescribed* as a set of *wildcard schemata*, 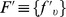. A wildcard schema 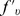 is exactly like a LUT entry, but allows an additional *wildcard* symbol, 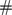 (also represented graphically in grey), to appear in its condition (see Figure 5B). A wildcard input means that it *accepts any state, leaving the transition unchanged*. In other words, wildcard inputs are *redundant* given the non-wildcard input states specified in a schema 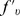. More formally, when the truth value of an input Boolean variable 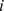 in a schema 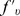 is defined by the third (wildcard) symbol, it is equivalent to stating that the truth value of automaton 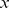 is unaffected by the truth value of 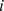 given the conditions defined by 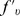: 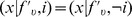. Each schema redescribes a subset of entries in the original LUT, denoted by 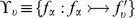 (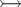 means ‘is redescribed by’).

Wildcard schemata are *minimal* in the sense that none of the (non-wildcard) inputs in the condition of a schema can be ‘raised’ to the wildcard status and still ensure the automaton's transition to the same state. Because wildcard schemata are minimal, 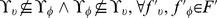. In other words, a wildcard schema is *unique* in the sense that the subset of LUT entries it redescribes is not fully redescribed by any other schema. However, in general 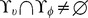. This means that schemata can overlap in terms of the LUT entries they describe. In Figure 5, 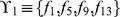 and 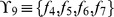, therefore 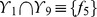. The set of wildcard schemata 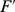 is also *complete*. This means that for a given LUT 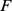 there is one and only one set 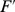 that contains all possible minimal and unique wildcard schemata. Since wildcard schemata are *minimal, unique* and they form a *complete* set 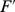, they are equivalent to the set of *all prime implicants* obtained during the first step of the Quine & McCluskey Boolean minimization algorithm [40]. Typically, prime implicants are computed for the fraction of the LUT that specifies transitions to *on*. Then a subset of the so-called *essential* prime implicants is identified. The set of essential prime implicants is the subset of prime implicants sufficient to describe (cover) every entry in the input set of LUT entries. However, to study how to control the transitions of automata we use the set of all prime implicants, since it encodes every possible way a transition can take place. The set 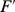 may also contain any original entry in 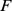 that could not be subsumed by a wildcard schema. Although the upper bound on the size of 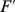 is known to be 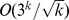 [41], the more input redundancy there is, the smaller the cardinality of 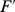.

The condition of a wildcard schema can always be expressed as a logical conjunction of literals (logical variables or their negation), which correspond to its non-wildcard inputs. Since a wildcard schema is a *prime implicant*, it follows that every literal is *essential* to determine the automaton's transition. Therefore, we refer to the literals in a schema as its *essential input states*, or *enputs* for short. To summarize, each enput in a schema is essential, and the conjunction of its enputs is a sufficient condition to *control* the automaton's transition. It also follows that the set 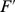 of wildcard schemata can be expressed as a *disjunctive normal form* (DNF) – that is, a disjunction of conjunctions that specifies the list of sufficient conditions to control automaton 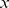, where each disjunction clause is a schema. The DNF comprising all the prime implicants of a Boolean function 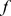 is known as its *Blake's canonical form* [42]. The canonical form of 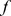 always preserves the input-output relationships specified by its LUT 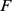. Therefore, the basic laws of Boolean logic – contradiction, excluded middle and de Morgan's laws – are preserved by the schema redescription.

Schema redescription is related to the work of John Holland on condition/action rules to model inductive reasoning in cognitive systems [43] and to the general *RR framework* proposed by Annette Karmiloff-Smith to explain the emergence of internal representations and external notations in human cognition [44]. Our methodology to remove redundancy from automata LUTs also bears similarities with the more general *mask analysis* developed by George Klir in his ‘reconstructability’ analysis, which is applicable to any type of variable [45]. In addition, prime implicants have been known and used for the minimization of circuits in electrical engineering since the notion was introduced by Quine & McCluskey [40]; similar ideas were also used by Valiant [46] when introducing *Probably Approximately Correct* (PAC) learning.

#### Two-symbol schemata

We now introduce a different and complementary form of redundancy in automata, which we consider another form of canalization. Wildcard schemata identify input states that are sufficient for controlling an automaton's transition (enputs). Now we identify subsets of inputs that can be permuted in a schema without effect on the transition it defines [39]. For this, a further redescription process takes as input the set of wildcard schemata (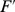) of 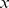 to compute a set of two-symbol schemata 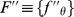 (see Figure 5C). The additional *position-free symbol* (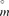) above inputs in the condition of a schema 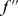 means that *any subset of inputs thus marked can ‘switch places’ without affecting the automaton's transition.* The index of the position-free symbol, when necessary, is used to differentiate among distinct subsets of ‘permutable’ inputs. A two-symbol schema 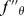 redescribes a set 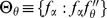 of LUT entries of 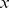; it also redescribes a subset 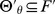 of wildcard schemata.

A two-symbol schema 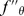 captures *permutation redundancy* in a set of wildcard schemata 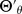. More specifically, it identifies subsets of input states whose permutations do not affect the truth value of the condition, leaving the automaton's transition unchanged. In group theory, a permutation is defined as a bijective mapping of a non-empty set onto itself; a permutation group is any set of permutations of a set. Permutation groups that consist of *all* possible permutations of a set are known as *symmetric groups* under permutation [47]. For Boolean functions in general, the study of permutation/symmetric groups dates back to Shannon [48] and McCluskey [49] (see also [50]).

Two-symbol schemata identify subsets of wildcard schemata that form symmetric groups. We refer to each such subset of input states that can permute in a two-symbol schema – those marked with the same position-free symbol – as a *group-invariant enput*. Note that a group-invariant enput may include wildcard symbols marked with a position-free symbol. More formally, a two-symbol schema 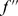 can be expressed as a logical conjunction of enputs – literal or group-invariant. Let us denote the set of literal enputs on the condition of 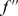 by 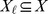 – the non-wildcard inputs not marked with the position-free symbol. For simplicity, 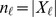.

A group-invariant enput 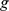 is defined by (1) a subset of input variables 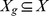 that are marked with an identical position-free symbol, and (2) a *permutation constraint* (a bijective mapping) on 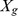 defined by the expression 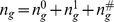, where 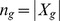, 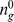 is the number of inputs in 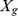 in state 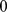 (off), and 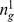 is the number of inputs in 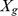 in state 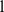 (on). We further require that at least two of the quantities 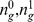 and 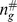 are positive for any group-invariant enput 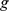. We can think of these two required positive quantities as *subconstraints*; in particular, we define a group-invariant enput by the two subconstraints 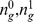, since 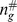 is always derivable from those two given the expression for the overall permutation constraint. This precludes the trivial case of subsets of inputs in the same state from being considered a valid group-invariant enput – even though they can permute leaving the transition unchanged. A two-symbol schema 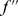 has 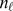 literal enputs and 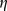 group-invariant enputs; each of the latter type of enputs is defined by a distinct permutation constraint for 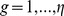. An input variable whose truth value is the wildcard symbol in a given schema is never a literal enput (it is not essential by definition). However, it can be part of a group-invariant enput, if it is marked with a position-free symbol. Further details concerning the computation of wildcard and two-symbol schemata are available in Data *S2*.

In our working example, the resulting two-symbol schema (see Figure 5C) contains 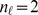 literal inputs: 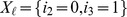. It also contains one (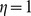) group-invariant enput 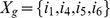 with size 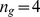 and subconstraints 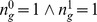. This redescription reveals that the automaton's transition to *on* is determined only by a subset of its six inputs: *as long as inputs 2 and 3 are off and on, respectively, and among the others at least one is on and another is off, the automaton will transition to on*. These minimal control constraints are not obvious in the original LUT and are visible only after redescription.

We equate *canalization* with redundancy. The more redundancy exists in the LUT of automaton 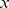, as input-irrelevance or input-symmetry (group-invariance), the more canalizing it is, and the more compact its two-symbol redescription is, thus 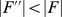. In other words – after redescription – input and input-symmetry redundancy in 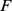 is removed in the form of the two symbols. The input states that remain are essential to determine the automaton's transition. Below we quantify these two types of redundancy, leading to two new measures of canalization. Towards that, we must first clearly separate the two forms of redundancy that exist in 2-symbol schemata. The *condition* of a two-symbol schema 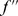 with a single group-invariant enput 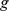 – such as the one in Figure 5C – can be expressed as:
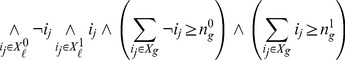
(1)where 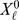 is the set of literal enputs that must be *off*, and 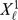 is the set of literal enputs that must be *on* (thus 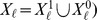. This expression separates the contributions (as conjunctions) of the literal enputs, and each subconstraint of a group-invariant enput. Since we found no automaton in the target model (see below) with schemata containing more than one group-invariant enput, for simplicity and without lack of generality, we present here only this case (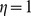). See *Data S3* for the general expression that accounts for multiple group-invariant enputs (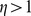).

All possible transitions of 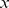 to *on* are described by a set 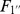 of two-symbol schemata. This set can also be expressed in a DNF, where each disjunction clause is given by Expression 1 for all schemata 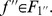 Transitions to *off* are defined by the negation of such DNF expression: 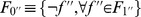. Canalization of an automaton 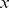 is now characterized in terms of two-symbol schemata that capture two forms of redundancy: (1) *input-irrelevance* and (2) *input-symmetry* (group-invariance). We next describe the procedure to compute 2-symbol schemata for a an automaton 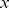. Readers not interested in the algorithmic details of this computation can safely move to the next subsection.

The procedure starts with the set of wildcard schemata 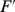 obtained via the first step of the Quine & McCluskey algorithm [40] (see above). The set 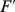 is then partitioned into subsets 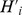 such that,
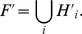
where each 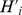 contains every schema 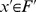 with equal number of zeroes (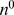), ones (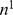), and wildcards (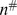), with 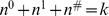. In other words, the 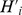 are equivalence classes induced on 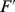 by 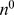, 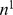, and 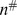. This is a necessary condition for a set of wildcard schemata to form a symmetric group. The algorithm then iterates on each 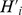, checking if it contains a symmetric group; i.e. if it contains wildcard schemata with all the permutations of the largest set of inputs variables possible. If it does, it marks those input variables as a group-invariant enput in 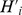 and moves to another subset 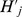. If it does not, then it checks for symmetric groups in smaller sets of input variables within each set 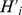. It does this by iteratively expanding the search space to include all subsets of 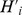 with cardinality 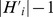. The procedure is repeated, if no symmetric groups are found, until the subsets contain only one wildcard schema.

Although several heuristics are implemented to prune the search space, the algorithm is often not suitable for exhaustively searching symmetric groups in large sets of schemata. However, the individual automata found in models of biochemical regulation and signalling networks typically have a fairly low number of inputs. Therefore, schema redescription of their LUT leads to manageable sets of wildcard schemata, which can be exhaustively searched for symmetric groups. Indeed, as shown below, all automata in the SPN model have been exhaustively redescribed into two-symbol schemata. For additional details on the computation of schemata see *Data S2*.

### Quantifying Canalization: Effective Connectivity and Input Symmetry

Schemata uncover the ‘control logic’ of automata by making the smallest input combinations that are necessary and sufficient to determine transitions explicit. We equate canalization with the redundancy present in this control logic: the smaller is the set of inputs needed to control an automaton, the more redundancy exists in its LUT and the more canalizing it is. This first type of canalization is quantified by computing the mean number of unnecessary inputs of automaton 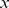, which we refer to as *input redundancy*. An upper bound is given by,
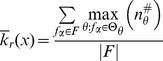
(2)and a lower bound is given by:



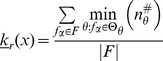
(3)These expressions compute a mean number of irrelevant inputs associated with the entries of the LUT 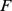. The number of irrelevant inputs in a schema 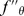 is the number of its wildcards 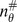. Because each entry 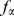 of 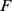 is redescribed by one or more schemata 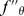, there are various ways to compute a characteristic number of irrelevant inputs associated with the entry, which is nonetheless bounded by the maximum and minimum number of wildcards in the set of schemata that redescribe 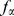. Therefore, the expressions above identify all schemata 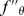 whose set of redescribed entries 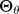 includes 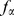. The upper (lower) bound of input redundancy, Equation 2 (Equation 3), corresponds to considering the maximum (minimum) number of irrelevant inputs found for all schemata 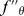 that redescribe entry 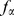 of the LUT – an optimist (pessimist) quantification of this type of canalization. Notice that input redundancy is not an estimated value. Also, it weights equally each entry of the LUT, which is the same as assuming that every automaton input is equally likely.

Here we use solely the upper bound, which we refer to henceforth simply as *input redundancy* with the notation 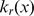. This means that we assume that the most redundant schemata are always accessible for control of the automaton. We will explore elsewhere the range between the bounds, especially in regards to predicting the dynamical behaviour of BNs. The range for input redundancy is 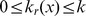, where 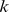 is the number of inputs of 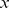. When 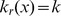 we have full input irrelevance, or maximum canalization, which occurs only in the case of frozen-state automata. If 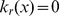, the state of every input is always needed to determine the transition and we have no canalization in terms of input redundancy.

In the context of a BN, if some inputs of a node 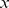 are irrelevant from a control logic perspective, then its *effective* set of inputs is smaller than its in-degree 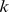. We can thus infer more about effective control in a BN than what is apparent from looking at structure alone (see analysis of macro-level control below). A very intuitive way to quantify such effective control, is by computing the mean number of inputs needed to determine the transitions of 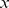, which we refer to as its *effective connectivity*:
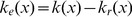
(4)whose range is 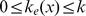. In this case, 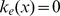 means full input irrelevance, or maximum canalization, and 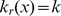, means no canalization.

The type of canalization quantified by the input redundancy and effective connectivity measures does not include the form of permutation redundancy entailed by group-invariant enputs. Yet this is a genuine form of redundancy involved in canalization, as in the case of nested canalization [14], since it corresponds to the case in which different inputs can be *alternatively* canalizing. The two-symbol schema redescription allows us to measure this form of redundancy by computing the mean number of inputs that participate in group-invariant enputs, easily tallied by the occurrence of the position-free symbol () in schemata. Thus we define a measure of *input symmetry* for an automaton 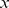, whose upper-bound is given by
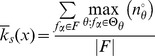
(5)and a lower-bound by,
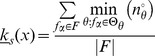
(6)where 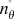 is the number of position-free symbols in schema 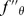.

The upper bound of input symmetry (Equation 5) corresponds to considering an optimist quantification of this type of canalization. Here we use solely the upper bound, which we refer to henceforth simply as input symmetry and denote by 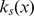. Again, the assumption is that the most redundant schemata are always accessible for control of the automaton. The range for input symmetry is 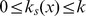. High (low) values mean that permutations of input states are likely (unlikely) to leave the transition unchanged.

Canalization in automata LUTs – the micro-level of networks of automata – is then quantified by two types of redundancy: *input redundancy* using 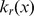 and *input symmetry* with 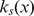. To be able to compare the canalization in automata with distinct numbers of inputs, we can compute *relative* measures of canalization:
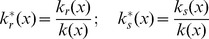
(7)the range of which is 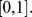 Automata transition functions can have different amounts of each form of canalization, which allows us to consider four broad canalization classes for automata: *class A* with high 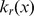 and high 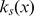, *class B* with high 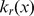 and low 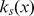, *class C* with low 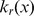 and high 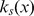, and *class D* with low 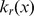 and low 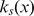. We will explore these classes in more detail elsewhere. Below, these measures are used to analyse micro-level canalization in the SPN model and discuss the type of functions encountered. Before that, let us introduce an alternative representation of the canalized control logic of automata, which allows us to compute network dynamics directly from the parsimonious information provided by schemata.

### Network Representation of a Schema

Canalization in an automaton, captured by a set of schemata, can also be conveniently represented as a McCulloch & Pitts threshold network – introduced in the 1940s to study computation in interconnected simple logical units [51]. These networks consist of binary units that can transition from quiescent to firing upon reaching an activity threshold 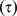 of the firing of input units. To use this type of network to represent two-symbol schemata we resort to two types of units. One is the *state unit* (s-unit), which represents an input variable in a specific Boolean state; the other is the *threshold unit* (t-unit) that implements the condition that causes the automaton to transition. Two s-units are always used to represent the (Boolean) states of any input variable that participates as enput in the condition of an automaton 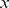: one fires when the variable is *on* and the other when it is *off*. To avoid contradiction, the two s-units for a given variable cannot fire simultaneously. Directed fibres link (source) units to (end) units, propagating a pulse – when the source unit is firing – that contributes to the firing of the end unit. The simultaneous firing of at least 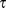 (threshold) incoming s-units into a t-unit, causes the latter to fire.

**Figure 6:**
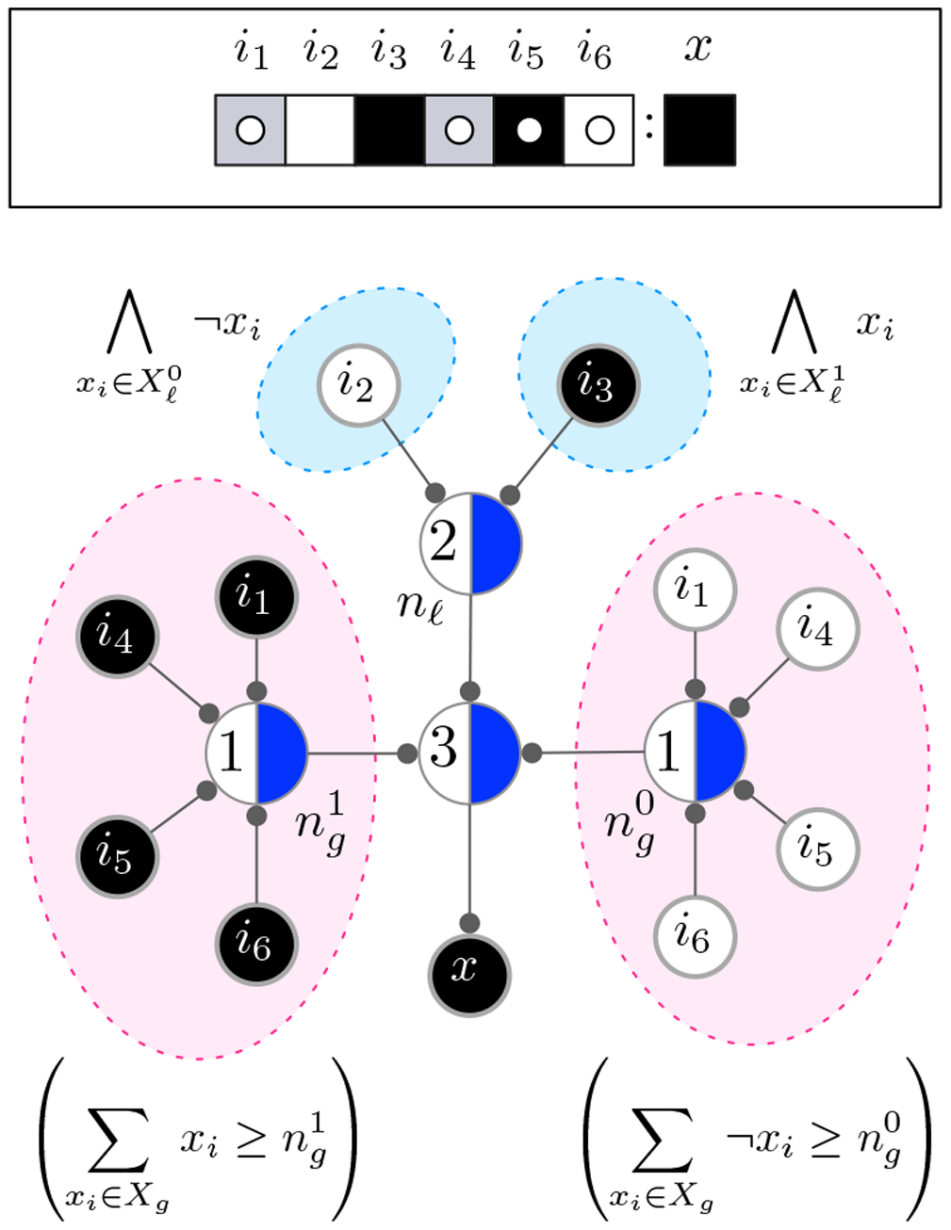
McCulloch & Pitts representation of Expression (1). The conjunction clauses in Expression (1) for the example automaton 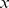 are directly mapped onto a standard McCulloch & Pitts network with two layers. On one layer the two literal enputs are accounted for by a threshold unit (at the top) with threshold 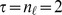. There is also a group-invariant enput with permutation subconstraints on both Boolean states. Two threshold units on the same layer are used to capture these. The threshold unit on the left accounts for the permutation subconstraint 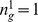. It thus has as incoming s-units the inputs 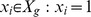 and threshold 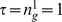. In a similar way, the threshold unit on the right accounts for the subconstraint 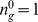. When all the constraints (literal and group-invariant) are satisfied, the last threshold unit (second layer) ‘fires’ causing the transition to *on*.

In the example automaton in Figure 5, the set of schemata 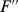 contains only one schema. This schema can be directly converted to a (2-layer) McCulloch & Pitts network. This conversion, which is possible due to the separation of subconstraints given by Expression (1), is shown in Figure 6 and explained in its caption. Note that in the McCulloch & Pitts representation, the transition of the automaton is determined in two steps. First, a *layer* of threshold units is used to check that the literal and group-invariant constraints are satisfied; then, a second layer – containing just one threshold unit – fires when every subconstraint in Expression (1) has been simultaneously satisfied, determining the transition. This means that in this network representation each schema with literal enputs and at least a group-invariant enput requires two layers and three t-units. Since in McCulloch & Pitts networks each threshold unit has a standard delay of one time step, this network representation of a schema takes two time steps to compute its transition. We introduce an alternative threshold network representation of a two-symbol schema 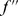 that only requires a single t-unit and takes a single time delay to compute a transition. We refer to this variant as the *Canalizing Map* of a schema or CM for short. A CM is essentially the same as a McCulloch and Pitts network, with the following provisos concerning the ways in which s-units and t-units can be connected:

1. Only one fibre originates from each s-unit that can participate as enput in 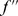 and it must always end in the t-unit used to encode 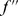.
2. The fibre that goes from a s-unit to the t-unit can *branch out* into several fibre endings. This means that if the s-unit is firing, a pulse propagates through its outgoing fibre and through its branches. Branching fibres are used to capture group-invariant enputs, as we explain later.
3. Branches from distinct s-units can *fuse* together into a single fibre ending – the fused fibre increases the end t-unit's firing activity by one if at least one of the fused fibres has a pulse.
4. A fibre that originates in a t-unit encoding a schema 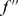 must end in a s-unit that corresponds to the automaton transition defined by 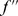.

**Figure 7:**
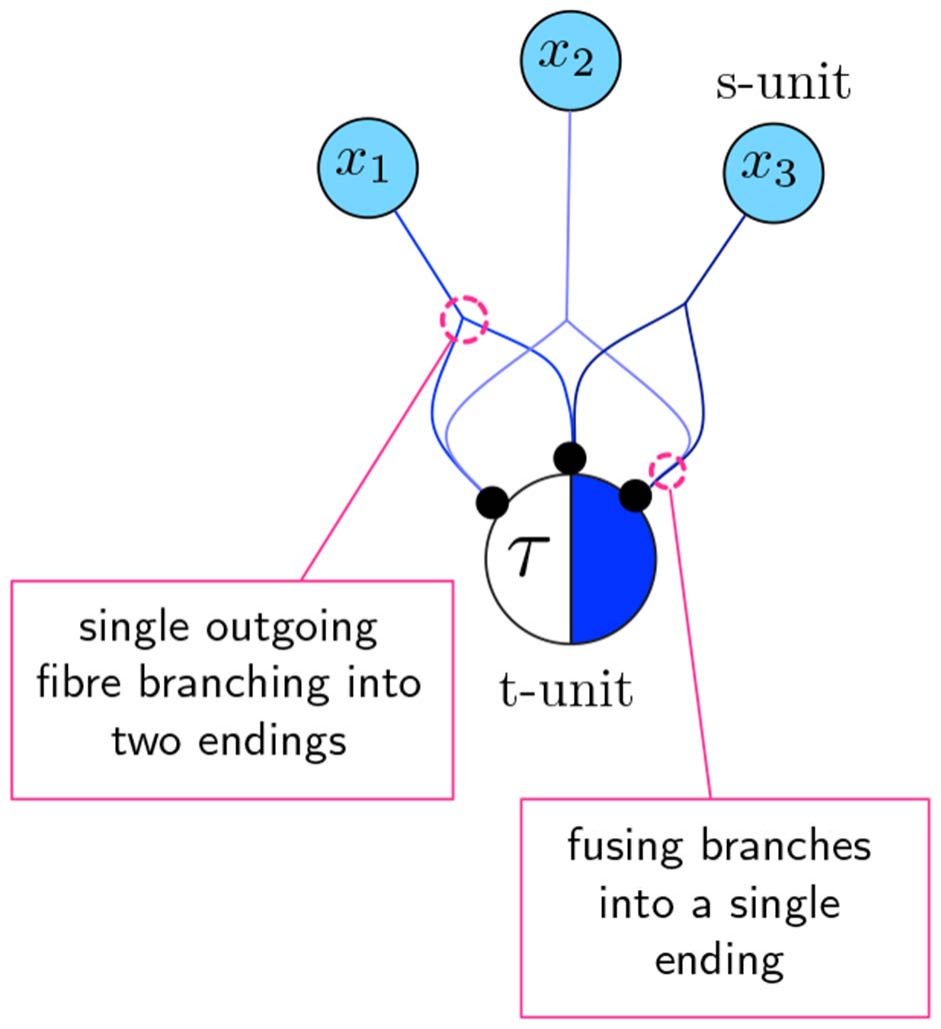
Elements of a Canalizing Map. Every s-unit is a circle, labelled according the automaton's input it represents and coloured according to its state: black is *on* and white is *off* (here we use light-blue for a generic state). The t-unit (schema) is represented using a larger circle. One of its halves is coloured, and the other labelled with the t-unit's threshold 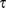. Fibres can be single, or branched. In this example there are branching fibres only, where fibre fusions represent all possible combinations of two out of the three s-units.

Figure 7 depicts the elements of a single schema's CM. Table 2 summarizes the rules that apply to the interconnections between units. Transitions in CMs occur in the same way as in standard McCulloch & Pitts networks. Once sufficient conditions for a transition are observed at some time 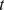, the transition occurs at 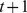. The firing (or not) of t-units is thus assumed to have a standard delay (one time-step), identical for all t-units. Note that in CMs, s-units can be regarded as a special type of t-unit with threshold 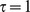 that send a pulse through their outgoing fibres instantaneously. Next we describe the algorithm to obtain the CM representation of a schema. Readers not interested in the algorithmic details of this computation can safely bypass the next subsection.

**Table 2:** Connectivity rules in canalizing maps.

	s-units	t-units
**incoming fibres**	one or more	one or more
**outgoing fibres**	one per schema of which is enput	one for the transition it causes
**branching out**	Yes	no
**fusing in**	No	yes

#### Algorithm to obtain the canalizing map of a schema

**Figure 8:**
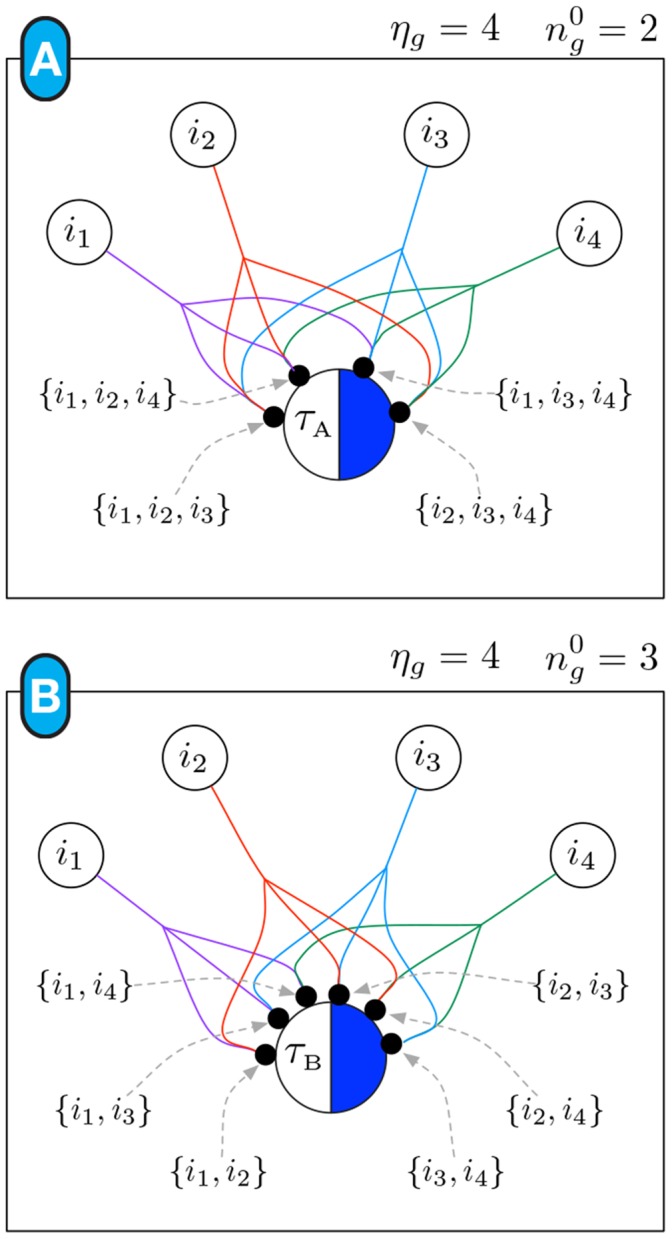
Canalizing map of example automaton 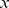
characterized by a single schema. (A) Since 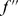 (shown on top) has 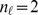, the corresponding s-units for literal enputs 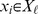 are directly linked to the t-unit for 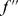 with single fibres; 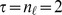. (B) Adding the subconstraint 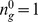 of the group-invariant enput 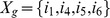. In this case, 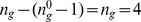, so there is only one subset 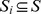 and thus a single branch from each s-unit in the group-invariant, fused into a single ending. The threshold becomes 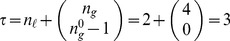. (C) Finally, we add the second subconstraint 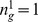 of the group-invariant enput 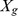, which has the same properties as the subconstraint integrated in (B). The final threshold of the t-unit is given by (9), therefore 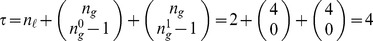. Notice that only the input-combinations that satisfy the constraints of Expression (1) for 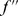 can lead to the firing of the t-unit; in other words, the canalizing map is equivalent to schema 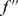.

Given a 2-symbol schema 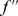, there are two steps involved in producing its CM representation. The first is connecting s-units to the t-unit for 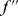 in such a way that it fires, if and only if, the constraints of 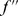 – defined by Expression (1) – are satisfied. The second step is determining the appropriate firing threshold 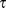 for the t-unit. If the schema does not have group-invariant enputs, the conversion is direct as for the standard McCulloch & Pitts network – see Figure 6: The s-units corresponding to literal enputs 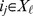 are linked to the t-unit using a single fibre from each s-unit to the t-unit, which has a threshold 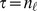. If the schema has a group-invariant enput, its subconstraints are implemented by branching and fusing fibres connecting the s-units and the t-unit. In cases such as our example automaton 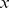 (Figures 5 and 6) where the subconstraints 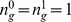, the solution is still simple. To account for subconstraint 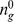, it is sufficient to take an outgoing fibre from each of the s-units 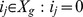 and fuse them into a single fibre ending. Therefore, if at least one of these s-units is firing, the fused fibre ending transmits a single pulse to the t-unit, signalling that the subconstraint has been satisfied. Increasing the t-unit's threshold by one makes the t-unit respond to this signal appropriately. The same applies for subconstraint 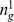, using a similar wiring for s-units 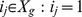. The final threshold for the t-unit that captures the example schema of Figure 5 is thus 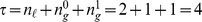, as shown in Figure 8C.

The case of general group-invariant constraints is more intricate. Every literal enput 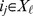 is linked to the t-unit via a single fibre exactly as above. Afterwards, the subconstraints 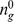 and 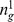 of a group-invariant enput 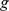 are treated separately and consecutively. Note that for every input variable 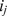 in the set 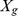 of symmetric input variables, there are two s-units: one representing 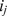 in state 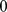 and another in state 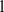. To account for subconstraint 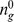 on the variables of set 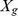, let 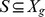 be the set of s-units that represent the variables of the group-invariant enput that can be in state 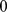, where 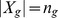. Next, we identify all possible subsets of 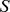, whose cardinality is 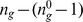. That is, 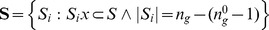. For each subset 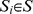, we take an outgoing fibre from every s-unit in it and fuse them into a single fibre ending as input to the schema t-unit. After subconstraint 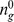 is integrated this way, the threshold of the t-unit is increased by,
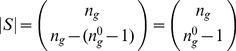
(8)


This procedure is repeated for the subconstraint 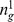 on 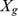. The final threshold of the t-unit is,
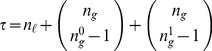
(9)


**Figure 9:**
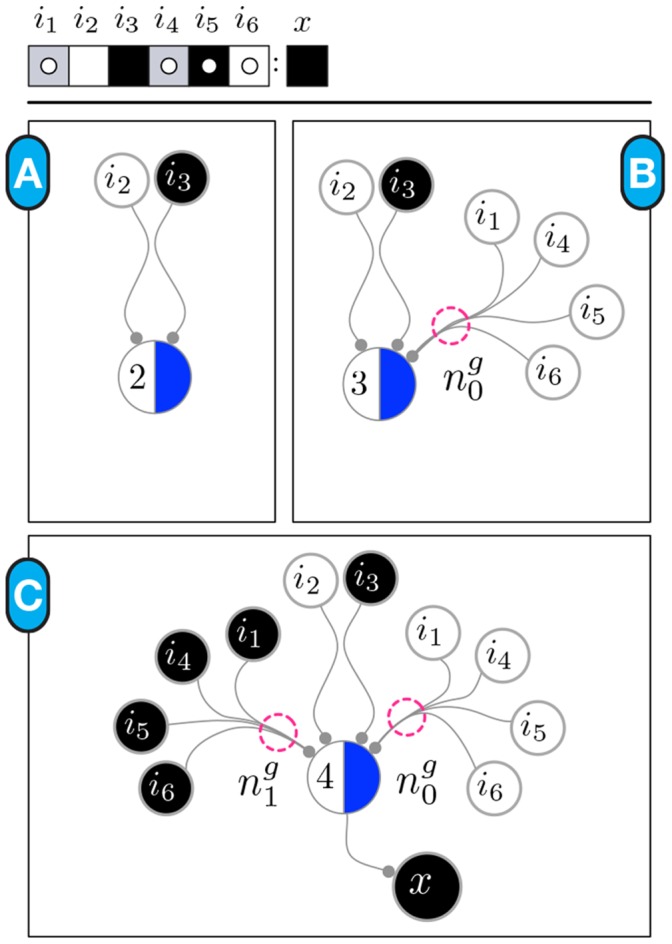
Procedure for obtaining the canalizing map of a group-invariant subconstraint. (A) subconstraint defined by 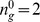, where 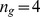. The first step is to consider the s-units (in state 0) for the four input variables in the group invariant enput 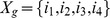. Next we identify all the subsets 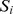 of these s-units containing 
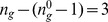
 s-units: 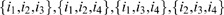 (shown with dotted arrows). From every s-unit in each such subset 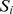, we take an outgoing fibre to be joined together into a single fibre ending as input to the t-unit. Finally, we increase the threshold of the t-unit by the total number of subsets, that is 
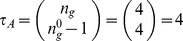
. (B) An example of the same procedure but for 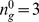 and 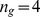: 
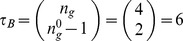
.

This algorithm is illustrated for the integration of two example subconstraints in Figure 9; in Figure 8, the case of the only schema describing the transitions to *on* of running example automaton 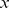 is shown. Further details concerning this procedure are provided in *Data S3.*

#### The canalizing map of an automaton

The algorithm to convert a single schema 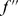 to a CM is subsequently used to produce the CM of an entire Boolean automaton 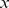 as follows: Each schema 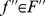 is converted to its CM representation. Each state of an input variable is represented by a single s-unit in the resulting threshold network. In other words, there is a maximum of two s-units (one for state 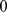 and one for state 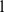) for each input variable that is either a literal enput or participates in a group-invariant enput of 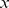. The resulting threshold network is the canalizing map of 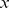. The connectivity rules of automata CMs include the following provisos:

1. Every s-unit can be connected to a single t-unit with a single outgoing fibre, which can be single or have branches.
2. Therefore, the number of outgoing fibres coming out of a s-unit (before any branching) corresponds to the number of schemata 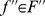 in which the respective variable-state participates as an enput. If such a variable is included in a group-invariant enput, then the fibre may have branches.
3. Any subset set of t-units with threshold 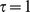 for the same automaton transition (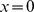 or 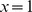) are merged into a single t-unit (also with 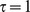), which receives all incoming fibres of the original t-units. In such scenario, any fused branches can also be de-fused into single fibres. Note that this situation corresponds to schemata that exhibit nested canalization, where one of several inputs settles the transition, but which do not form a symmetric group.

**Figure 10:**
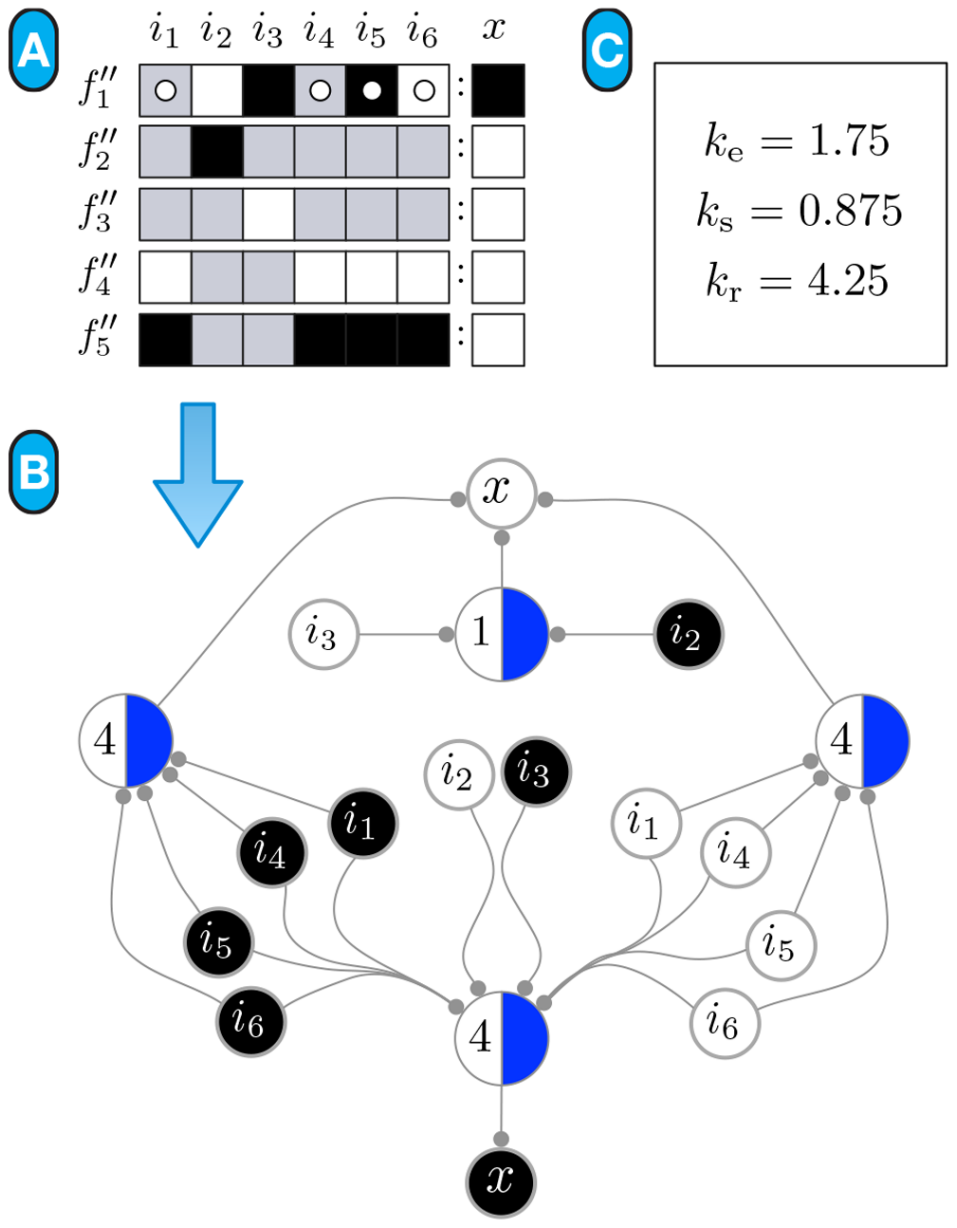
Canalizing Map of automaton x**.** (A) complete set of schemata 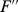 for 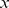, including the transitions to *on* shown in Figure 5 and the transitions of *off* (the negation of the first).(B) canalizing map; t-units for schemata 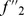 and 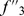 were merged into a single t-unit with threshold 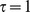 (see main text). (C) effective connectivity, input symmetry and input redundancy of 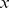.

The CM of 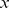 can be constructed from the subset of schemata 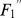 (the conditions to *on*), or 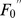 (the conditions to *off*). When the conditions are not met for convergence to *on*, one is guaranteed convergence to *off* (and vice-versa). However, since we are interested in exploring scenarios with incomplete information about the states of variables in networks of automata rather than a single automaton (see below), we construct the CM of a Boolean automaton 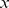 including all conditions, that is using 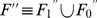. This facilitates the analysis of transition dynamics where automata in a network can transition to either state. Figure 10 depicts the complete CM of the example automaton 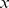 described in Figure 5– now including also its transitions to *off*.

By uncovering the enputs of an automaton, we gain the ability to compute its transition with *incomplete information* about the state of every one of its inputs. For instance, the possible transitions of the automaton in Figure 5 are fully described by the CM (and schemata) in Figure 10; as shown, transitions can be determined from a significantly small subset of the input variables in specific state combinations. For instance, it is sufficient to observe 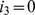 to know that automaton 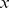 transitions to *off*. If 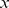 was used to model the interactions that lead a gene to be expressed or not, it is easy to see that to down-regulate its expression, it is sufficient to ensure that the regulator 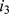 is not expressed. This is the essence of canalization: the transition of an automaton is controlled by a small subset of input states. In the macro-level canalization section below, we use the CM's ability to compute automata transitions with incomplete information to construct an alternative portrait of network dynamics, which we use in lieu of the original BN to study collective dynamics. Let us first apply our micro-level methodology to the SPN model.

### Micro-level Canalization in the SPN Model

**Figure 11:**
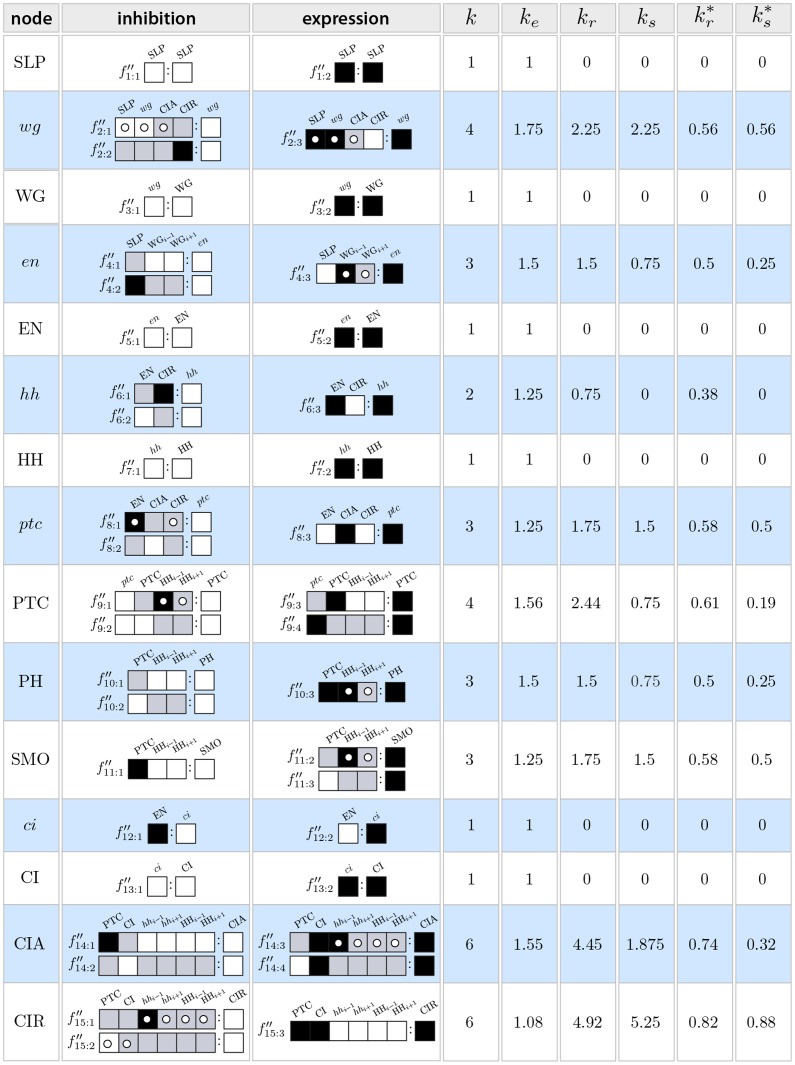
Micro-level canalization for the Automata in the SPN model. Schemata for inhibition (transitions to off) and expression (transitions to on) are shown for each node (genes or proteins) in model. In-degree (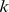), input redundancy (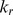), effective connectivity (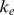), and input symmetry (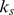) are also shown.

**Figure 12:**
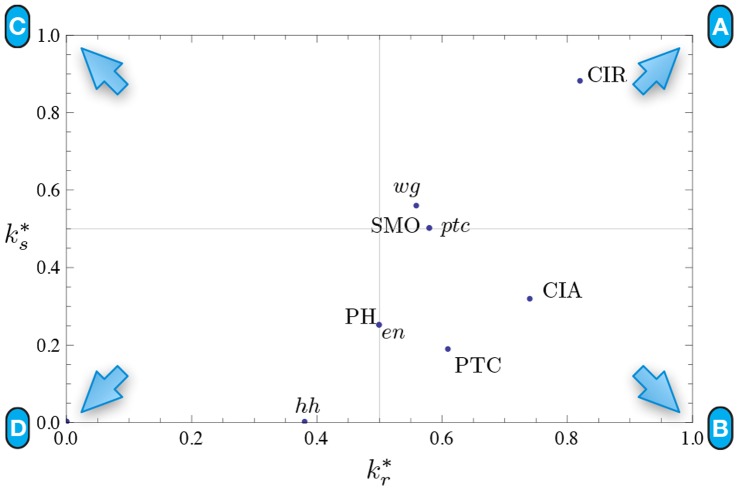
Quantification of canalization in the SPN automata. Relative input redundancy is measured in the horizontal axis (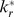) and relative input symmetry is measured in the vertical axis (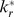). Most automata in the SPN fall in the class II quadrant, showing that most canalization is of the input redundancy kind, though nodes such as CIR and 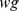 display strong input symmetry too.

**Figure 13:**
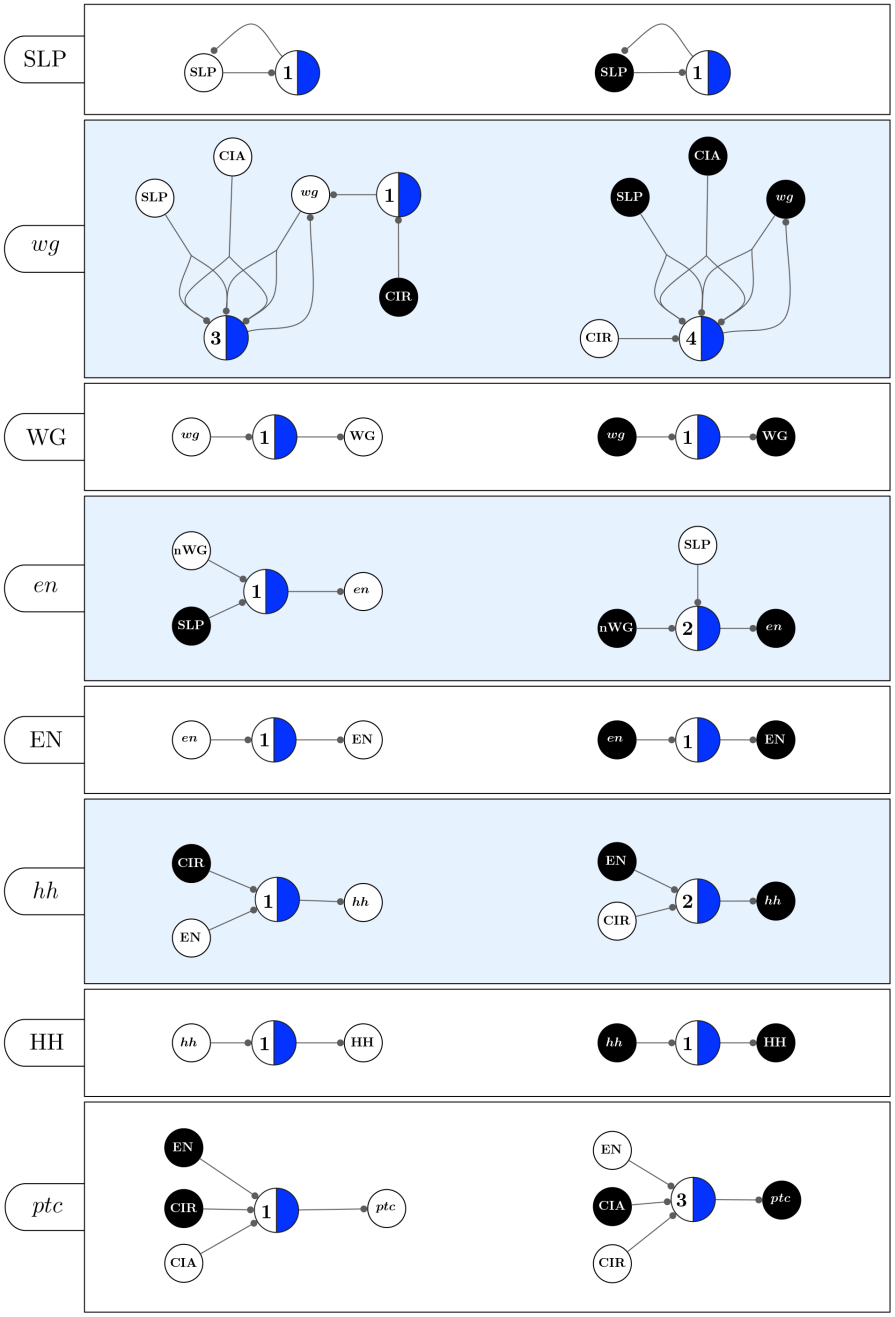
Canalizing Maps of individual nodes in the SPN model (part 1). The set of schemata for each automaton is converted into two CMs: one representing the minimal control logic for its inhibition, and another for its expression. Note that 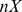 denotes the state of node 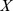 in both neighbour cells: 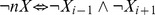 and 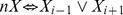, where 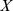 is one of the spatial-signals 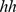, HH, or WG (see text).

**Figure 14:**
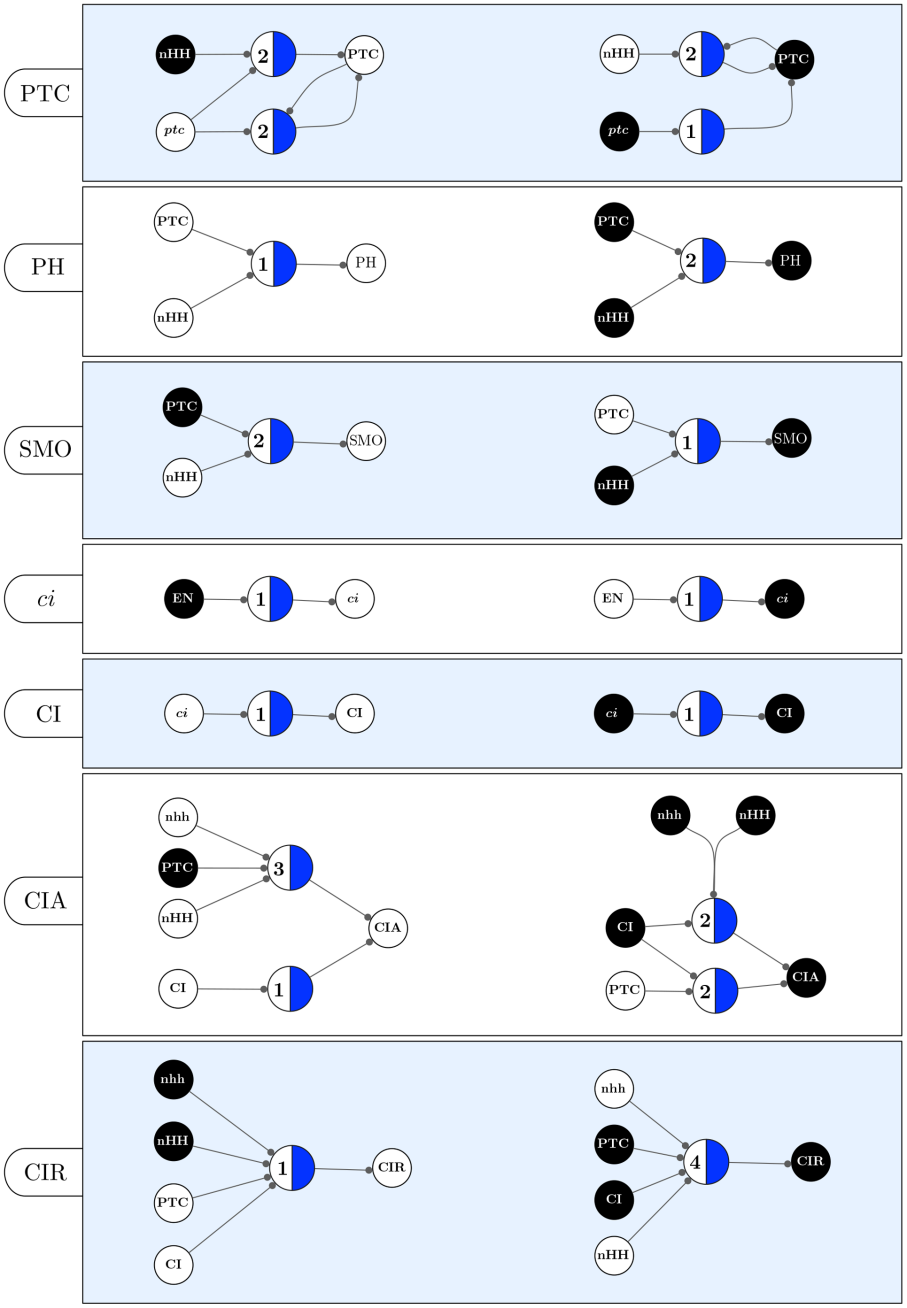
Canalizing Maps of individual nodes in the SPN model (cont). The set of schemata for each automaton is converted into two CMs: one representing the minimal control logic for its inhibition, and another for its expression. Note that 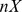 denotes the state of node 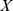 in both neighbour cells: 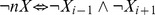 and 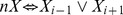, where 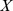 is one of the spatial-signals 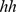, HH, or WG (see text).

The automata in the SPN fall in two categories: those that have a single input (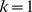), the analysis of which is trivial, namely, SLP, WG, EN, HH, 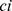 and CI, and those with 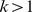. The two-symbol schemata and canalization measures for each automaton in the SPN model are depicted in Figure 11; Figure 12 maps the automata to their canalization classes. Schemata easily display all the sufficient combinations of input states (enputs) to control the transitions of the automata in this model, which represent the inhibition or expression of genes and proteins. Indeed, the resulting list of schemata allows analysts to quickly infer how control operates in each node of the network. Wildcard symbols (depicted in Figure 11 as grey boxes) denote redundant inputs. Position-free symbols (depicted in Figure 11 as circles), denote ‘functionally equivalent’ inputs; that is, sets of inputs that can be alternatively used to ensure the same transition. For example, for 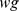 to be expressed, SLP, the previous state of 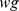 (reinforcing feedback loop) and CIA can be said to be ‘functionally equivalent’, since any two of these three need to be expressed for 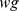 to be expressed. The several schemata that are listed for the expression or inhibition of a specific node (genes and gene products), give experts alternative ‘recipes’ available to control the node according to the model – and which can be experimentally tested and validated. Let us now present some relevant observations concerning micro-level canalization in the SPN model:

1. The inhibition of 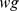 can be attained in one of two ways: either two of the first three inputs (SLP, 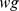, CIA) are *off* (unexpressed), or CIR is *on* (expressed). The expression of 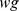 – essential in the posterior cell of a parasegment to attain the wild-type expression pattern (Figure 3)– is attained in just one way: CIR must be *off* (unexpressed), and two of the other three inputs (SLP, 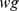, CIA) must be *on* (expressed). Note the simplicity of this control logic vis a vis the 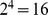 possible distinct ways to control 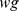 specified by its LUT, given that it is a function of 4 inputs. This control logic is also not obvious from the Boolean logic expression of node 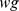, as shown in Table 1; at the very least, the schemata obtained for 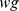 provide a more intuitive representation of control than the logical expression. Moreover, schema redescription, unlike the logical expression, allows us to directly quantify canalization. The control logic of this gene shows fairly high degree of both types of canalization: even though there are 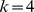 inputs, on average, only 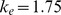 inputs are needed to control the transition, and 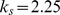 inputs can permute without effect on the transition (see Figures 11 and 12); 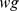 is thus modelled by an automaton of class A.
2. The inhibition of CIR can be attained in one of two simple, highly canalized, ways: either one of its first two inputs (PTC, CI) is *off* (unexpressed), or one of its four remaining inputs (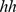 and 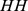 in neighbouring cells) is *on* (expressed); all other inputs can be in any other state. The expression of CIR can be attained in only one specific, non-canalized, way: the first two inputs must be *on* (expressed), and the remaining four inputs must be *off* (unexpressed) – a similar expression behaviour is found for 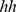 and 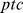. Note the simplicity of this control logic vis a vis the 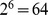 possible distinct ways to control CIR specified by its LUT, given that it is a function of 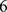 inputs. While, in this case, the control logic is also pretty clear from the original Boolean logic expression of node CIR (in Table 1), the schemata obtained for CIR provide a more intuitive representation of control and allows us to directly quantify canalization. CIR is a protein with a very high degree of both types of canalization: even though there are 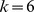 inputs, on average, only 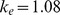 inputs are needed to control the transition, and 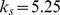 inputs can permute without effect on the transition (see Figures 11 and 12). This high degree of both types of canalization, which is not quantifiable directly from the logical expression or the LUT, is notable in Figure 12, where CIR emerges very clearly as an automaton of class A.
3. The control logic of CIA entails high canalization of the input redundancy kind. For instance, its inhibition can be achieved by a single one of its six inputs (CI *off*) and its expression by two inputs only (PTC *off* and CI *on*). On the other hand, there is low canalization of the input symmetry kind, therefore CIA is modelled by an automaton in class B.
4. The expression of 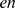 – essential in the anterior cell of a parasegment to achieve the wild-type phenotype – depends on the inhibition of (input node) SLP in the same cell, and on the expression of the wingless protein in at least one neighbouring cell.
5. Most automata in the model fall into canalization class B described above. CIR and 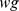 discussed above display greatest input symmetry, and fall in class A (see Figure 12).
6. Looking at all the schemata obtained in Figure 11, we notice a consistent pattern for all spatial signals, 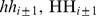 and 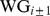. Whenever they are needed to control a transition (when they are enputs in the schemata of other nodes), either they are *off* in both neighbouring cells, or they are *on* in at least one of the neighbouring cells. For instance, for a given cell 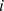, HH in neighbouring cells is only relevant if it is unexpressed in both cells (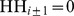), or if it is expressed in at least one of them (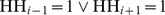). This means that the six nodes corresponding to spatial signals affecting a cell in a parasegment can be consolidated into just three *neighbour nodes*, a similar consolidation of spatial signals was used previously by Willadsen & Wiles [52] to simplify the spatial model into a single-cell non-spatial model. In what follows, we refer to these spatial signals simply as 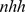, nHH and nWG. If such a node is *off* it means that the corresponding original nodes are *off* in both adjacent cells; if it is *on* it means that at least one of the corresponding original nodes in an adjacent cell is *on*.
7. Only PTC and 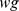 have feedback loops that are active after schema redescription, both for their inhibition and expression; these are self-reinforcing, but also depend on other enputs (see also Figures 13 and 14).

Because this is a relatively simple model, some of the observations about control, especially for nodes with fewer inputs, could be made simply by looking at the original transition functions in Table 1, since they are available as very simple logical expressions – this is the case of CIR, but certainly not 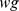 above. However, the *quantification* of canalization requires the additional symbols used in schema redescription to identify redundancy, which are not available in the original automata logical expressions or their LUTs. Moreover, the transition functions of automata in larger Boolean models of genetic regulation and signalling are rarely available as simple logical expressions, and nodes can be regulated by a large number of other nodes, thus making such direct comprehension of control-logic difficult. In contrast, since redescription uncovers canalization in the form of input redundancy and symmetry, the more canalization exists, the more redundancy is removed and the simpler will be the schemata representation of the logic of an automaton. This makes canalizing maps (CM) particularly useful, since they can be used to visualize and compute the minimal control logic of automata. The CMs that result from converting the schemata of each node in the SPN to a threshold-network representation are shown in Figure 13 and Figure 14. For a biochemical network of interest, such as the SPN or much larger networks, domain experts (e.g. biomedical scientists and systems and computational biologists) can easily ascertain the control logic of each component of their model from the schemata or the corresponding CMs.

In summary, there are several important benefits of schema redescription of Boolean automata vis a vis the original Boolean logic expression or the LUT of an automaton: (1) a parsimonious and intuitive representation of the control logic of automata, since *redundancy is clearly identified* in the form of the two additional symbols, which gives us (2) the ability to *quantify* all forms of canalization in the straightforward manner described above; finally, as we elaborate next, the integration of the schema redescription (or CMs) of individual automata in a network (micro-level) allows us to (3) *characterize macro-level dynamics* parsimoniously, uncovering minimal control patterns, robustness and the modules responsible for collective computation in these networks.

### Macro-level Canalization and Control in Automata Networks

After removing redundancy from individual automata LUTs in networks (micro-level), it becomes possible to integrate their canalizing logic to understand control and collective dynamics of automata networks (macro-level). In other words, it becomes feasible to understand how biochemical networks process information collectively – their emergent or collective computation [39], [53]–[56].

#### Dynamics canalization map and dynamical modularity

The CMs obtained for each automaton of a BN, such as the SPN model (see Figures 13 and 14), can be integrated into a single threshold network that represents the control logic of the entire BN. This simple integration requires that (1) each automaton is represented by two unique s-units, one for transition to *on* and another to *off*, and (2) s-units are linked via t-units with appropriate fibres, as specified by each individual CM. Therefore a unique t-unit represents each schema obtained in the redescription process. This results in the *Dynamics Canalization Map* (DCM) for the entire BN. Since the DCM integrates the CMs of its constituent automata, it can be used to identify the *minimal control conditions* that are sufficient to produce transitions in the dynamics of the entire network. Notice that when a node in the original BN undergoes a state-transition, it means that at least one t-unit fires in the DCM. When a t-unit fires, according to the control logic of the DCM, it can cause subsequent firing of other t-units. This allows the identification of the *causal chains of transitions* that are the *building blocks* of macro-level dynamics and information processing, as explained in detail below.

Another important feature of the DCM is its compact size. While the dynamical landscape of an automata network, defined by its state-transition graph (STG), grows exponentially with the number of nodes – 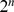 in Boolean networks – its DCM grows only linearly with 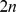 units plus the number of t-units needed (which is the number of schemata obtained from redescribing every automaton in the network): 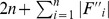. Furthermore, the computation of a DCM is tractable even for very large networks with thousands of nodes, provided the in-degree of these nodes is not very large. In our current implementation, we can exhaustively perform schema redescription of automata with 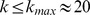; that is, LUTs containing up to 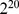 entries. It is very rare that dynamical models of biochemical regulation have molecular species that depend on more than twenty other variables (see e.g. [57]). Therefore, this method can be used to study canalization and control in all discrete models of biochemical regulation we have encountered in the literature, which we will analyse elsewhere.

It is important to emphasize that the integration of the CMs of individual automata into the DCM does not change the control logic encoded by each constituent CM, which is equivalent to the logic encoded in the original LUT (after removal of redundancy). Therefore, there is no danger of violating the logic encoded in the original LUT of any automaton in a given BN. However, it is necessary to ensure that any initial conditions specified in the DCM do not violate the laws of contradiction and excluded middle. This means, for instance, that no initial condition of the DCM can have the two (*on* and *off*) s-units for the same automaton firing simultaneously.

**Figure 15:**
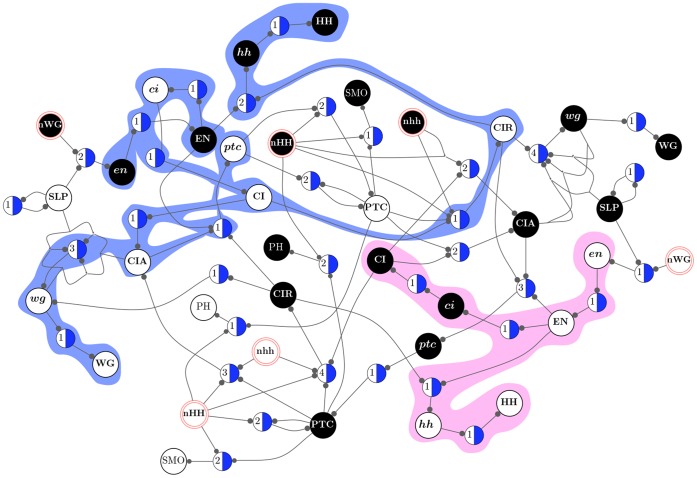
Dynamics Canalization Map for a single cell of the SPN Model. Also depicted are pathway modules 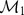 (pink) and 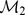 (blue), whose initial conditions depend exclusively on the expression and inhibition of input node SLP and of WG in neighbouring cells (the nWG spatial-signals). 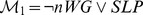, 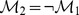 (see details in text).

The DCM for a single cell in the SPN model is shown in Figure 15. The spatial signals from adjacent cells are highlighted using units with a double border 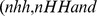
n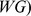. For the simulations of the spatial SPN model described in subsequent sections, we use four coupled single-cell DCMs (each as in Figure 15) to represent the dynamics of the four-cell parasegment, where nodes that enable inter-cellular regulatory interactions are appropriately linked, as defined in the original model. Also, as in the original model, we assume periodic boundary conditions for the four-cell parasegment: the posterior cell is adjacent to the anterior cell. When making inferences using the DCM, we use *signal* to refer to the firing of a s-unit and the transmission of this information through its output fibres. When a s-unit fires in the DCM, it means that its corresponding automaton node in the original BN transitioned to the state represented by the s-unit. We also use *pathway* to refer to a logical sequence of signals in the DCM.

We highlight two *pathway modules* in the DCM of the SPN in Figure 15: 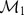 and 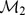. The first is a pathway initiated by either the inhibition of WG in neighbour cells, or the expression of SLP upstream in the same cell. That is, the initial pattern for this module is 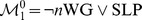. The initiating signal for 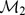 is defined by the negation of those that trigger the first: 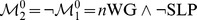. Both modules follow from (external or upstream) input signals to a single cell in the SPN; they do not depend at all on the initial states of nodes (molecular species) of the SPN inside a given cell. Yet, both of these very small set of initial signals necessarily cause a cascade of other signals in the network over time. 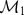 is the only pathway that leads to the inhibition of 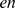 (and EN) as well as the expression of 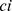 (and CI). It also causes the inhibition of 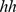 and HH, both of which function as inter-cellular signals for adjacent cells – this inhibition can be alternatively controlled by the expression of CIR, which is not part of neither 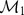 nor 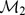. Since 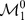 is a disjunction, its terms are equivalent: either the inhibition of 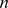
WG or the upstream expression of SLP control the same pathway, regardless of any other signals in the network. 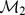 is the only pathway that leads to the expression of 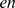 (and EN) as well as the inhibition of 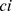 (and CI); It also causes the inhibition of CIA, 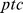 and CIR – these inhibitions can be alternatively controlled by other pathways. If the initial conditions 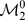 are sustained for long enough (steady-state inputs), the downstream inhibition of CIA and sustained inhibition of SLP lead to the inhibition of 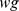 (and WG); likewise, from sustaining 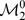, the downstream expression of EN and inhibition of CIR lead to the expression of 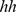 (and HH). Since 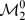 is a conjunction, both terms are required: both the expression of 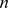
WG and the upstream inhibition of SLP are necessary and sufficient to control this pathway module, regardless of any other signals in the network.


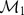
 and 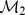 capture a cascade of state transitions that are inexorable once their initiating signals (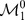 and 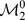) are observed: 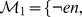 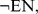 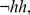 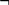 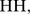 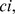 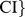 and 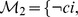 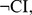 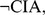 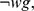 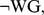 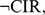 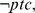 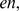 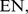 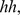 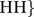. Furthermore, these cascades are *independent* from the states of other nodes in the network. As a consequence, the transitions within a module are insensitive to delays once its initial conditions are set (and maintained in the case of 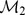 as shown). The *dynamics* within these portions of the DCM can thus be seen as *modular*; these pathway modules can be *decoupled* from the remaining regulatory dynamics, in the sense that they are not affected by the states of any other nodes other than their initial conditions. Modularity in complex networks has been typically defined as sub-graphs with high intra-connectivity [21]. But such structural notion of community structure does not capture the dynamically decoupled behaviour of pathway modules such as 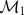 and 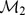 in the SPN. Indeed, it has been recently emphasized that understanding modularity in complex molecular networks requires accounting for dynamics [58], and new measures of modularity in multivariate dynamical systems have been proposed by our group [59]. We will describe methods for automatic detection of dynamical modularity in DCMs elsewhere.

Collective computation in the macro-level dynamics of automata networks ultimately relies on the interaction of these pathway modules. Information gets integrated as modules interact with one another, in such a way that the timing of module activity can have an effect on downstream transitions. For instance, the expression of CI via 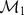 can subsequently lead to the expression of CIA, provided that 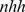 is expressed – and this is controlled by 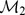 in the adjacent cells. The expression of CI can also be seen as a necessary initial condition to the only pathway that results in the expression of CIR, which also depends on the inhibition of 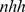 and 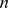 HH and the expression of PTC, which in turn depends on the interaction of other modules, and so on. As these examples show, pathway modules allow us to uncover the building blocks of macro-level control – the collective computation of automata network models of biochemical regulation. We can use them, for instance, to infer which components exert most control on a target collective behaviour of interest, such as the wild-type expression pattern in the SPN. Indeed, modules 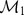 and 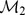 in the SPN model, which include a large proportion of nodes in the DCM, highlight how much SLP and the spatial signals from neighbouring cells control the dynamical behaviour of segment polarity gene regulation in each individual cell. Particularly, they almost entirely control the expression and inhibition of EN and WG; as discussed further below. The behaviour of these proteins across a four-cell parasegment mostly define the attractors of the model (including wild-type). The transitions of intra-cellular nodes are thus more controlled by the states of ‘external’ nodes than by the initial pattern of expression of genes and proteins in the cell itself. This emphasizes the well-known spatial constraints imposed on each cell of the fruit fly's developmental system [60], [61]. We next study and quantify this control in greater detail.

#### Dynamical unfolding

A key advantage of the DCM is that it allows us to study the behaviour of the underlying automata network without the need to specify the state of all of its nodes. Modules 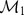 and 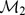 are an example of how the control that a very small subset of nodes exerts on the dynamics of SPN can be studied. This can be done because, given the schema redescription that defines the DCM, subsets of nodes can be assumed to be in an *unknown* state. Since the schema redescription of every automaton in the DCM is *minimal* and *complete* (see micro-level canalization section), every possible transition that can occur is accounted for in the DCM. By implementing the DCM as a threshold network, we gain the ability to study the dynamics of the original BN by setting the states of subsets of nodes. This allows us study convergence to attractors, or other patterns of interest, from knowing just a few nodes.

More formally, we refer to an initial pattern of interest of a BN 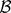 as a *partial configuration*, and denote it by 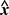. For example, 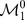 is a partial configuration 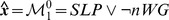, where the states of all other nodes is 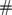, or unknown. We refer to *dynamical unfolding* as the sequence of transitions that necessarily occur after an initial partial configuration 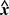, and denote it by 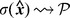, where 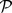 is an *outcome pattern* or configuration. From the DCM of the single-cell SPN model (Figure 15), we have 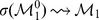 and 
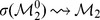
. An outcome pattern can be a fully specified attractor 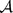, but it can also be a partial configuration of an attractor where some nodes remain unknown – for instance, to study what determines the states of a specific subset of nodes of interest in the network. In the first case, it can be said that 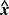 *fully controls* the network dynamics towards attractor 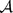. In the second, control is exerted only on the subset of nodes with determined logical states.

The ability to compute the dynamical unfolding of a BN from partial configurations is a key benefit of the methodology introduced here: it allows us to determine how much partial configurations of interest *control* the collective dynamics of the network. For instance, in the SPN model it is possible to investigate how much the input nodes to the regulatory network of each cell control its dynamics. Or, conversely, how much the initial configuration of the intra-cellular regulatory network is irrelevant to determining its attractor. The nodes within each cell in a parasegment of the SPN are sensitive to three inter-cellular (external) input signals: 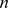
WG, 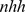 and 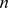
HH, and one intra-cellular (upstream) input, SLP. Given that the formation of parasegment boundaries in *D. melanogaster* is known to be tightly spatially constrained [60], [61], it is relevant to investigate how spatio-temporal control occurs in the SPN model. We already studied the control power of SLP and 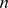
WG, which lead to modules 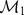 and 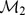. We now exhaustively study the dynamical unfolding of all possible states of the intra- and inter-cellular input signals.

**Figure 16:**
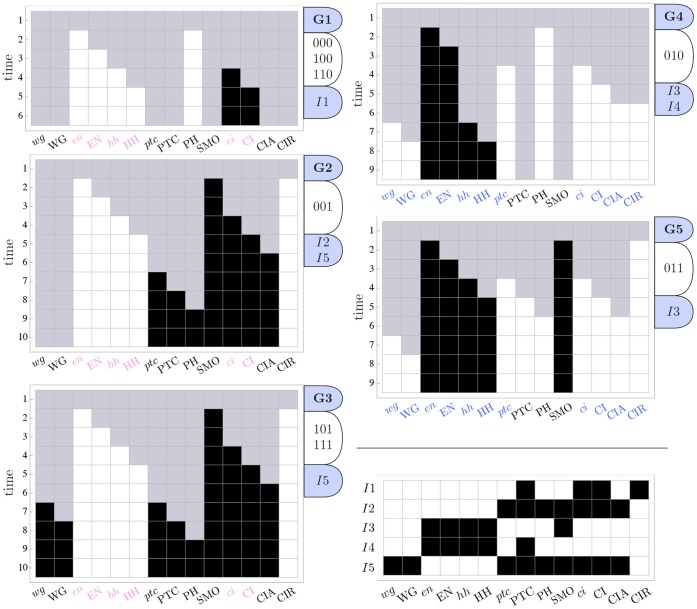
Dynamical unfolding of the (single-cell) SPN with partial input configurations. The eight initial partial configurations tested correspond to the combinations of the steady-states of intra- and inter-cellular inputs SLP, 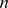
WG and 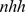 (and where 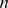
HH and 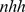 are merged into a single node, 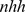). The specific state-combinations of these three variables is depicted on the middle (white) tab of each dynamical unfolding plot. Initial patterns that reach the same target pattern are grouped together in five groups 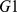 to 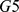 (identified in the top tab of each plot). The six input configurations in groups G1, G2, and G3 depict the dynamics where pathway module 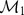 is involved (nodes involved in this module are highlighted in pink.) The two input configurations in G4 and G5 depict the dynamics where pathway module 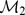 is involved (nodes involved in this module are highlighted in blue.) Three of the eight combinations are in 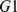 because they reach the same final configuration which, although partial, can only match the attractor 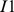. There are five possible attractor patterns of the SPN model for a single cell, shown in bottom right inset: 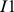 to 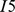 (see 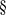 background). Attractors reached by each group are identified in the bottom tab of each plot. Groups 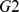 and 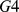 both unfold to an ambiguous target pattern that can end in 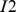 or 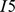 for 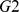, and 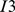 or 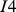 for 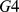. Finally, the initial partial configurations in groups 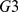 and 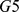 are sufficient to resolve the states of every node in the network.

We assume that SLP (upstream) and the (external) spatial signals are in steady-state to study what happens in a single cell. Since the state of 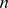
HH is the same as 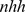 after one time step, we consolidate those input signals into a single one: 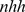. We are left with three input signals to the intra-cellular regulatory network: nodes SLP, 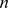
WG and 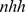. Each of these three nodes can be in one of two states (*on*, *off*) and thus there are eight possible combinations of states for these nodes. Such simplification results in a non-spatial model and this was done previously by Willadsen & Wiles [52]. Setting each such combination as the initial partial configuration 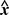, and allowing the DCM to compute transitions, yields the results shown in Figure 16. We can see that only two of the outcome patterns reached by the eight input partial configurations are ambiguous about which of the final five possible attractors is reached. Each individual cell in a parasegment can only be in one of five attractor patterns 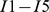 (see 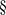 background). This is the case of groups 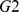 and 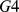 in Figure 16. For all the other input partial configurations, the resulting outcome pattern determines the final attractor. We also found that for almost every input partial configuration, the states of most of the remaining nodes are also resolved; in particular the nodes that define the signature of the parasegment attractor – Engrailed (EN) and Wingless (WG) – settle into a defined steady-state. Notice also that for two of the input partial configurations (groups 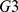 and 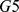 in Figure 16), the states of every node in the network settle into a fully defined steady-state. The picture of dynamical unfolding from the intra- and inter-cellular inputs of the single-cell SPN network also allows us to see the roles played by modules 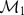 and 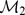 in the dynamics. The six input configurations in groups G1, G2, and G3 depict the dynamics where 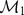 is involved, while the two input configurations in G4 and G5 refer to 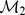 (node-states of each module in these groups appear shaded in Figure 16). By comparing the resulting dynamics, we can see clearly the effect of the additional information provided by knowing if 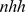 is expressed or inhibited; we also see that the dynamics of the modules is unaffected by other nodes, as expected.

It is clear from these results that (single-cell) cellular dynamics in the SPN is almost entirely controlled from the inputs alone. We can say that extensive micro-level canalization leads the macro-level network dynamics to be highly canalized by external inputs – a point we explore in more detail below. For the dynamical unfolding depicted in Figure 16 we assumed that the three input signals to the intra-cellular regulatory network are in steady-state, focusing on a single cell. This is not entirely reasonable since inter-cellular signals are regulated by spatio-temporal regulatory dynamics in the full spatial SPN model. We thus now pursue the identification of *minimal* partial configurations that guarantee convergence to outcome patterns of interest in the spatial SPN model, such as specific (parasegment) attractors.

#### Minimal configurations

To automate the search of minimal configurations that converge to patterns of interest, we rely again on the notion of schema redescription, but this time for network-wide configurations rather than for individual automata LUTs. Notice that the eight input partial configurations used in the dynamical unfolding scenarios described in Figure 16 are wildcard schemata of network configurations: the state of the 14 inner nodes is *unknown* (wildcard), and only three (input) nodes (SLP, nWG,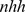) are set to a combination of Boolean states. Each of these eight schemata redescribes 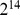 possible configurations of the single-cell SPN. Six of the eight input schemata converge to one of the five possible attractors for inner nodes in a single cell of the SPN model (Figure 16). We can thus think of those six schemata as *minimal configurations* (MCs) that guarantee convergence to patterns (e.g. attractors) of interest.

More specifically, a MC is a 2-symbol schema 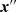 that redescribes a set of network configurations that converge to target pattern 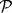; when the MC is a wildcard schema, it is denoted by 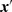. Therefore, 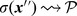. MC schemata, 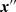 or 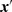, are network configurations where the truth value of each constituent automaton can be 0, 1, or 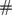 (unknown); symmetry groups are allowed for 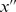 and identified with position-free symbols 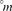 (see Micro-level canalization section). An MC schema redescribes a subset 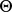 of the set of configurations 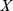: 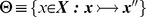. A partial configuration is a MC if no Boolean state in it can be raised to the unknown state (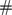) and still guarantee that the resulting partial configuration converges to 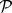. In the case of a two-symbol schema, no group-invariant enput can be enlarged (include additional node-states) and still guarantee convergence to 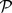. Finally, the target pattern 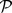 can be a specific network configuration (e.g. an attractor), or it can be a set of configurations of interest (e.g. when only some genes or proteins are expressed). After redescription of a set of configurations 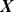 of a BN – a subset or its full dynamical landscape – we obtain a set of two-symbol MCs 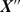; a set of wildcard MCs is denoted by 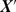. Similarly to micro-level schemata, we can speak of enputs of MCs. In this context, they refer to individual and sets of node-states in the network that are essential to guarantee convergence to a target pattern.

The dynamical unfolding example of the single-cell SPN model shows that to converge to the attractor 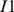 (Figure 16, G1), only the states of the three input nodes need to be specified, in one of three possible Boolean combinations: 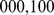 or 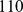 for the nodes SLP, 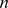
WG and 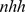; all other (inner) nodes may be unknown (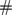). Moreover, these three initial patterns can be further redescribed into two schemata: 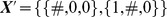. This shows that to guarantee converge to 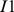, we only need to know the state of two (input) nodes: either 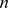
WG 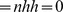, or SLP  = 1 and 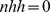. All other nodes in the single-cell model can remain unknown. Therefore, the MCs for attractor pattern 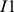 are:
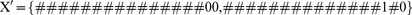
(10)


where the order of the inner nodes is the same as in Figure 16, and the last three nodes are SLP, 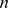
WG and 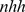 in that order. Notice that in this case there is no group-invariance, so 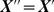. Any initial configuration not redescribed by 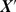, does not converge to pattern 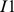. Therefore, these MCs reveal the enputs (minimal set of node-states) that *control* network dynamics towards attractor 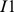: 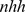 must remain unexpressed, and we must have either SLP expressed, or 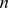
WG unexpressed. However, as mentioned above, this example refers to the case when the three input nodes are in steady-state. For the single-cell SPN, the steady-state assumption is reasonable. But for the spatial SPN, with parasegments of four cells, we cannot be certain that the spatial signals (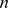
WG and 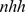) have reached a steady-state at the start of the dynamics. Therefore, we now introduce a procedure for obtaining MCs, without the steady-state assumption, which we apply to the spatial SPN network model.

It was discussed previously that individual automata in BN models of biochemical regulation and signalling very rarely have large numbers of input variables. This allows tractable computation of two-symbol schema redescription of their LUTs (see micro-level section). In contrast, computing MCs for network configurations easily becomes more computationally challenging. Even for fairly small networks with 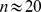, the size of their dynamical landscape becomes too large to allow full enumeration of the possible configurations and the transitions between them. As shown above, it is possible to identify pathway modules, and to compute dynamical unfolding on the DCM, without knowing the STG of very large BNs, but it remains not feasible to fully redescribe their entire dynamical landscape.

One way to deal with high-dimensional spaces is to resort to *stochastic search* (see e.g. [62]). We use stochastic search to obtain MCs that are guaranteed to converge to a pattern of interest 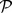. We start with a *seed* configuration known to converge to 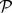. Next, a random node in a Boolean state is picked, and changed to the unknown state. The resulting partial configuration is then allowed to unfold to determine if it still converges to 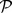. If it does, the modified configuration becomes the new seed. The process is repeated until no more nodes can be ‘raised’ to the unknown state and still ensure convergence to 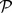. Otherwise, the search continues picking other nodes. The output of this algorithm (detailed in *Data S4*) is thus a single wildcard MC. Afterwards, the goal is to search for *sets* of MCs that converge to 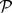. We do this in two steps: first we search for a set of MCs derived from a single seed, followed by a search of the space of possible different seeds that still converge to 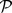. We use two ‘tolerance’ parameters to determine when to stop searching. The first, 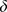, specifies the number of times a single seed must be ‘reused’ in the first step. When the algorithm has reused the seed 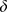 consecutive times without finding any new MCs, the first step of the MC search stops. The second tolerance parameter, 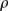, is used to specify when to stop searching for new seeds from which to derive MCs. When 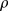 consecutively generated random (and different) seeds are found to be already redescribed by the current set of MCs, the algorithm stops. Both parameters are reset to zero every time a new MC is identified. These two steps are explained in greater detail in *Data S4*.

The two-step stochastic search process results in a set of wildcard schemata 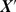 that redescribe a given set of configurations 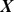 guaranteed to converge to pattern 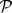. We next obtain a set of two-symbol MCs 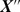 from 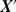, by identifying group-invariant subsets of nodes using the same method described in the micro-level canalization section. Since 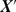 can be quite large (see below), this computation can become challenging. In this case, we restrict the search for symmetric groups in 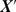 that redescribe a minimum number 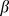 of wildcard MCs 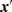.

Notice that it is the DCM, implemented as a threshold network, that allows us to pursue this stochastic search of MCs. With the original BN, we cannot study dynamics without setting every automaton to a specific Boolean truth value. With the DCM, obtained from micro-level canalization, we are able to set nodes to the unknown state and study the dynamical unfolding of a partial configuration (see previous subsection) to establish convergence to a pattern of interest. Therefore, the DCM helps us link micro-level canalization to macro-level behaviour. Let us exemplify the approach with the SPN model.

We started our study of MCs in the spatial SPN model, with a set of *seed* configurations 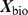 that contains the known initial configuration of the SPN (shown in Figure 3), the wild-type attractor (Figure 4a), and the five configurations in the dynamic trajectory between them. When searching for MCs using these seed configurations we set 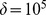. This resulted in a set containing 90 wildcard MCs 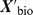 (available in *data S7*). Using the set 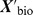, we performed the two-step stochastic search with 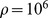 and 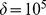. This resulted in a much larger set of 1745 wildcard MCs (available in *data S8*) which guarantee convergence to wild-type: 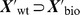. The number of literal enputs in each MC contained in this set varies from 23 to 33 – out of the total 60 nodes in a parasegment. In other words, from all configurations in 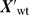 we can ascertain that to guarantee convergence to the wild-type attractor, we need only to control the state of a minimum of 23 and a maximum of 33 of the 60 nodes in the network. Equivalently, 27 to 37 nodes are irrelevant in steering the dynamics of the model to the wild-type attractor – a high degree of canalization we quantify below.

We chose to study two further subsets of 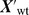 separately: 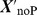 and 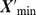. The first (available in *data S9*) is the subset of MCs that do not have enputs representing expressed (*on*) proteins, except SLP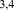 – since SLP in cells 3 and 4 is assumed to be present from the start, as determined by the pair-rule gene family (see [26] and introductory section). This is a subset of interest because it corresponds to the expected control of the SPN at the start of the segment-polarity dynamics, including its known initial configuration (Figure 3); thus 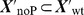. The second, 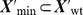 is the subset of MCs with the smallest number of enputs (available in *data S10*. This corresponds to the set of 32 MCs in 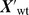 that have only 23 enputs each. This is a subset of interest because it allows us to study how the unfolding to wild-type can be guaranteed with the smallest possible number of enputs. Notice that 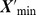 redescribes a large subset of configurations in 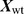 because it contains the MCs with most redundant number of nodes. These sets of wildcard MCs are available in *data S7,S8, S9* and *S10*; Table 3 contains their size.

**Table 3:** Macro-level canalization in the wildcard MC sets converging to wild-type in the SPN.

MC set	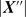	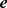 (min)	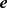 (max)	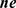	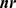	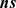
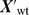	1745	23	33	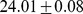	35.99 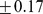	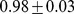
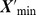	32	23	23	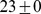	37 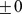	0
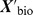	90	25	28	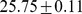	34.25 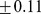	0
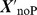	24	26	30	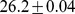	34.8 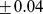	0

The table lists for every set of MCs reported in the main text: cardinality, minimum number of enputs, maximum number of enputs, estimated canalization. Canalization measures were obtained, for each MC set, from 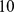 independent samples of 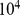 configurations, thus 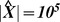. Values shown refer to the mean plus 95% confidence intervals for the 10 independent measurements.

There are severe computational limitations to counting exactly the number of configurations redescribed by each set of MCs, since it depends on using the inclusion/exclusion principle [63] to count the elements of intersecting sets (MCs redescribe overlapping sets of configurations). See *Data S6* for further details. We can report the exact value for 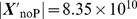, which is about 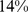 of the number of configurations – or pre-patterns – estimated by Albert & Othmer [26] to converge to the wild-type attractor 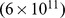. Using the inclusion/exclusion principle, it was also computationally feasible to count the configurations redescribed by a sample of 20 of the 32 MCs in 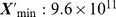. Since this sample of 20 MCs is a subset of 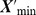, which is a subset of 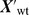, we thus demonstrate that 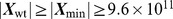, which is 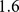 times larger than the previously estimated number of pre-patterns converging to the wild-type attractor [26]. This means that the wild-type attraction basin is considerably (at least 1.6 times) larger than previously estimated, with a lower bound of at least 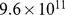 network configurations. Although it was not computationally feasible to provide exact counts for the remaining MC sets, it is reasonable to conclude that the set 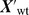 redescribes a significant proportion of the wild-type attractor basin, given the number of configurations redescribed by 20 of its most canalized MCs in comparison to the previous estimate of its size. Indeed, we pursued a very wide stochastic search with large tolerance parameters, arriving at a large number (1745) MCs, each of which redescribes a very large set of configurations. For instance, each MC with the smallest number of enputs (23) alone redescribes 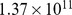 configurations, which is about 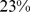 of the original estimated size of the wild-type attractor basin, and 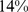 of the lower bound for the size of the attractor basin we computed above. Given the large number of MCs in the 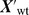 set, even with likely large overlaps of configurations, much of the attractor basin ought to be redescribed by this set.

**Figure 17:**
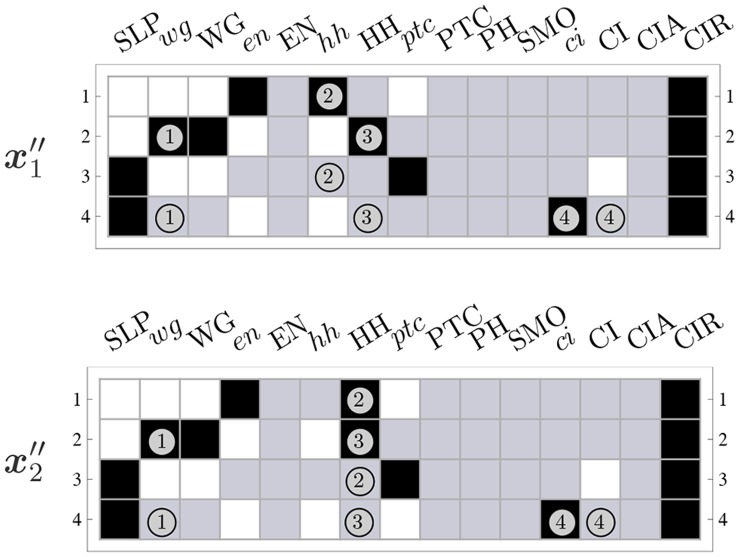
Two-Symbol schemata with largest number of position-free symbols, obtained from redescription of ***X_wt_*** **.** The pair 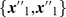 were the two-symbol schemata obtained in our stochastic search; both include 4 pairs of symmetric node-pairs, each denoted by a circle and a numerical index.

From 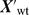, we derived two-symbol MC sets using 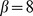. That is, due to the computational limitations discussed previously, we restricted the search to only those two-symbol MCs 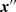 that redescribe at least 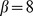 wildcard MCs 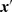. Given that configurations of the spatial SPN are defined by 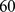 automata states, the group-invariance enputs we may have missed with this constraint are rather trivial. For instance, we may have missed MCs with a single group-invariant enput of 3 variables (any group-invariant enput with 4 variables would be found), or MCs with 2 distinct group-invariant enputs of 2 variables each (any MCs with 3 group-invariant enputs would be found.) With this constraint on the search for two-symbol MCs, we identified only the pair of two-symbol MCs depicted in Figure 17: 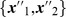 – each redescribing 16 wildcard MCs – the MCs redescribed are available in *data S13*. These two MCs redescribe 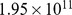 configurations; that is, about 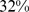 of the wild-type attraction basin as estimated by [26], or 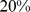 of the lower bound for the size of the attractor basin we computed above – a very substantial subset of the wild-type attractor basin.

No other two-symbol MCs redescribing at least eight wildcard MCs were found in the set 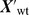. Therefore, 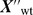 is comprised of the wildcard MCs in 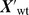 with the addition of 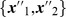 and removal of the wildcard MCs these two schemata redescribe. Table 3 contains the size of all MC sets. Moreover, 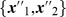 have no intersecting schemata with the additional three subsets of 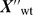 we studied. This means that the two-symbol redescription (with 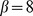) is equal to the wildcard redescription of the sets of configurations 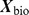, 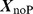 and 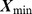. The pair of two-symbol MCs identified denote two very similar minimal patterns that guarantee convergence to the wild-type attractor. In both MCs, the pairs of nodes 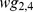, HH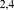 as well as 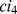 and CI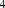 are marked with distinct position-free symbols. In other words, they have three identical group-invariant enputs. For 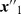 a fourth group-invariant enput comprises the nodes 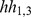, while for 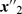 the fourth group-invariant enput contains the nodes HH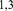. For 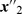 there is an extra literal enput: 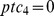 (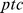 gene in fourth cell is unexpressed). The remaining literal enputs are identical to those of 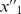. The group-invariance in these MCs is not very surprising considering the equivalent roles of neighbouring hedgehog and Wingless for intra-cellular dynamics – as discussed previously when the SPN's DCM was analysed. Notice that most group-invariance occurs for the same genes or proteins in alternative cells of the parasegment; for instance, 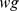 expressed in either cell 2 or cell 4. Nonetheless, both two-symbol MCs offer two minimal conditions to guarantee convergence to the wild-type attractor, which includes a very large proportion of the wild-type attractor basin. Therefore, they serve as a parsimonious prescription for analysts who wish to control the macro-level behaviour (i.e. attractor behaviour) of this system. Finally, the MCs obtained observe substantial macro-level canalization which we quantify below.

### Quantifying Macro-level Canalization

In the micro-level canalization section, we defined measures of *input redundancy*, *effective connectivity* and *input symmetry* to quantify micro-level canalization from the schema redescription of individual automata. Since we can also redescribe configurations that produce network dynamics, leading to the minimal configurations (MCs) of the previous section, we can use very similar measures to quantify macro-level canalization and control. At the macro-level, high canalization means that network dynamics are more easily controllable: MCs contain fewer necessary and sufficient node-states (enputs) to guarantee convergence to an attractor or target pattern 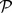. Similarly to the micro-level case, we first define upper and lower bounds of *node redundancy* computed from the set of MCs 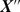 for a target pattern:
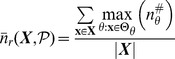
(11)

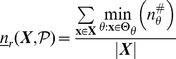
(12)


These expressions tally the mean number of irrelevant nodes in controlling network dynamics towards 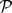 for all configurations 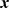 of a set of configurations of interest 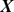 (e.g. a basin of attraction). The number of irrelevant nodes in a given MC 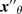 is the number of its wildcards 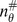. Because each configuration 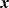 is redescribed by one or more MCs, there are various ways to compute a characteristic number of irrelevant nodes associated with the configurations, which is nonetheless bounded by the maximum and minimum number of wildcards in the set of MCs that redescribe 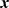. Therefore, the expressions above identify all MCs whose set of redescribed configurations 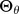 includes 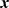. The upper (lower) bound of node redundancy, Equation 11 (Equation 12), corresponds to considering the maximum (minimum) number of irrelevant nodes found for all MCs that redescribe configuration 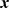 of the interest set – an optimist (pessimist) quantification of this type of macro-level canalization. Here we use solely the upper bound, which we refer to henceforth simply as *node redundancy* with the notation 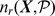. Similarly to the micro-level case, the assumption is that the most redundant MCs are always accessible for control of the network towards pattern 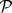. The range for node redundancy is 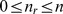, where 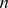 is the number of nodes in the network. When 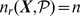 we have full node irrelevance, or maximum canalization, which occurs only in the case of networks where the state of every node is not dependent on any input (that is, when 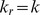 for every node). If 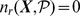, the state of every node is always needed to determine convergence to 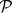 and we have no macro-level canalization.

If some nodes of a network are irrelevant to steer dynamics to 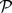, from a control logic perspective, we can say that 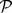 is effectively controlled by a subset of nodes of the network with fewer than 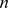 nodes. In other words, by integrating the micro-level control logic of automata in a network into the DCM, we are able to compute MCs and infer from those the macro-level *effective control*, which is not apparent from looking at connectivity structure alone:
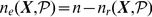
(13)whose range is 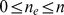. If 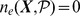 it means full node irrelevance, or maximum canalization. When 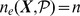, it means no canalization i.e. one needs to control all 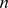 nodes to guarantee converge to 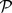.

Macro-level canalization can also manifest *alternative* control mechanisms. The two-symbol schema redescription allows us to measure this form of control by computing the mean number of nodes that participate in group-invariant enputs, easily tallied by the number of position-free symbols (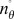) in MC schemata 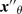 that characterize convergence to target pattern 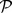. Thus, we quantify the upper and lower bounds of *node symmetry* in a set of configurations of interest 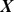 related to target pattern 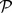 (e.g. a basin of attraction).
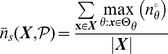
(14)

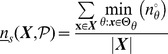
(15)


Here we use solely the upper bound, which we refer to henceforth simply as node symmetry and denote by 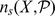; its range is 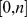. Again, the assumption is that the most canalized MCs are always accessible for control of the network towards pattern 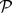. High (low) values mean that permutations of node-states are likely (unlikely) to leave the transition unchanged.

Macro-level canalization in network dynamics is then quantified by two types of redundancy: node redundancy (or its counterpart, effective control) and node symmetry. To be able to compare macro-level control in automata networks of different sizes, we can compute *relative* measures of canalization:
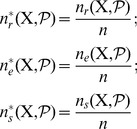
(16)whose range is 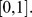 Network dynamics towards a pattern of interest 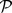 can have different amounts of each form of canalization, which allows us to consider four broad classes of control in network dynamics – just like the micro-level canalization case (see above).

The two MCs identified above for the single-cell SPN model (Eq. 10), redescribe the full set of configurations that converge to 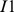. Since these MC schemata do not have group-invariant enputs, node symmetry does not exist: 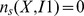. Node redundancy and effective control is 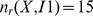 and 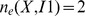, respectively. In other words, even though the network of the single-cell SPN model comprises 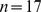 nodes, to control its dynamics towards attractor 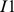, it is sufficient to ensure that the states of only two nodes remain fixed; the initial state of the other 15 nodes is irrelevant. More concretely, 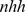 must remain *off* and either SLP remains *on* or 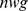 remains *off*. The relative measures become: 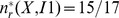 (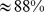 of nodes are redundant to guarantee convergence to attractor 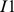) 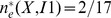 (one only needs to control 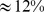 of nodes to guarantee convergence to attractor 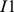), and 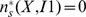 (there is no node symmetry in these MCs). This means that there is a large amount of macro-level canalization of the node redundancy type – and thus higher controllability – in the basins of attraction of the SPN model where pattern 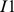 is present.

The macro-level canalization measures above assume that the interest set of configurations 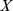 can be enumerated. Moreover, schema redescription of network configurations itself assumes that 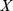 can be sufficiently sampled with our stochastic search method (see previous sub-section). The node symmetry measure additionally assumes that the set of wildcard MCs obtained by stochastic search is not too large to compute symmetric groups. While these assumptions are easily met for micro-level analysis, because LUT entries of individual automata in models of biochemical regulation do not have very large number of inputs, they are more challenging at the macro-level. Certainly, canalization in the single-cell SPN model can be fully studied at both the micro- and macro-levels – see Figures 11 and 12 for the former as well as example above for the latter. But quantification of macro-level canalization of larger networks, such as the spatial SPN model, needs to be estimated. Therefore, in formulae 11, 12, 14, and 15, the set of configurations 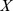 is sampled: 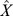. Configurations for 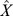 are sampled from each MC in the set 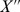, proportionally to the number of configurations redescribed by each MC – i.e. roulette wheel sampling. Configurations from a selected MC are sampled by ascribing Boolean truth values to every wildcard in the MC schema; the proportion of each of the truth values is sampled from a uniform distribution. If a selected MC is a 2-symbol schema, the truth-values of group-invariant enputs are also sampled from a uniform distribution of all possible possibilities. Naturally, the same configuration 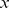 can be redescribed by more than one MC 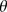. In summary, macro-level canalization for larger networks is quantified with the estimated measures: 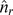, 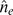, and 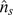, as well as their relative versions.

Tables 3 and 4 summarize the quantification of macro-level canalization estimated for the four MC sets obtained above: 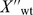, 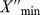, 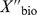, and 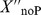. Effective control (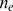) ranges between 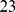 and 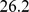 nodes (out of 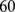) for the four sets of MCs; this means (see 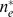) that only 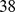 to 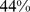 of nodes need to be controlled to guarantee convergence to wild-type. This shows that there is substantial macro-level canalization in the wild-type attractor basin; from 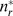, we can see that 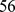 to 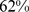 of nodes are, on average, redundant to guarantee convergence to wild-type. On the other hand, macro-level canalization in the form of alternative (or symmetric) control mechanisms is not very relevant on this attractor basin, as observed by the low values of 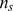 and 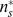: in the wild-type attractor basin, on average, only approximately 1 out 60 nodes, or 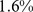 can permute.

**Table 4:** Macro-level canalization in the wildcard MC sets converging to wild-type in the SPN.

MC set	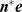	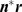	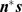
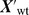	0.4 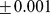	0.6 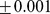	0.016 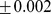
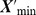	0.38	0.62	0
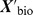	0.43 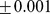	0.57 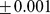	0
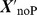	0.436 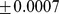	0.564 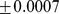	0

The table lists the relative canalization measures for every set of MCs reported in the main text. Canalization measures were obtained, for each MC set, from 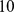 independent samples of 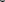 configurations, thus 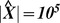. Values shown refer to the mean plus 95% confidence intervals for the 10 independent measurements.

### Enput Power and Critical Nodes

Every MC is a schema, and hence comprises a unique set of enputs, not entirely redescribed by any other MC. As defined in the micro-level canalization section, an enput 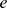 can be literal – a single node in a specific Boolean state – or a group-invariant enput: a set of nodes with a symmetry constraint. Every enput 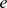 in a given MC is essential to ensure convergence to a pattern 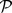, e.g. an attractor 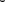. Consequently, if the state or constraint of 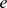 is disrupted in the MC, without gaining additional knowledge about the configuration of the network, we cannot guarantee convergence to 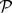. How *critical* is 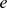 in a set of configurations 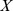 redescribed by an MC set 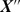 – such as the set of MCs that redescribe a basin of attraction? Since there are usually alternative MCs that redescribe the possible dynamic trajectories to 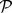, the more 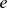 appears in 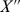, the more critical it is in guaranteeing convergence to 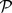.

For instance, in the two MCs shown in Equation 10, the enput 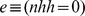 is common to both. Therefore, disrupting it, without gaining additional knowledge about the state of other nodes, would no longer guarantee convergence to the attractor pattern 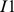 in the single-cell SPN dynamics. Similarly, for the two-symbol MC set of the spatial SPN model, shown in Figure 17, enputs 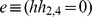 and group-invariant enput 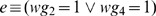 appear in both MCs. Disrupting them, would no longer guarantee convergence to wild-type attractor in the spatial SPN dynamics.

Let us quantify the potential disruption of target dynamics by perturbation of enputs in an MC set. The *power* of an enput 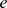 in a set of configurations 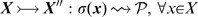, is given by:



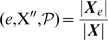
(17) where 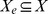 is the subset of configurations redescribed by 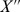 that contain enput 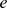: 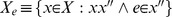. Thus, this measure yields the proportion of configurations in 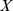 redescribed by the MCs in which 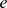 is an enput; its range is 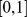. If an enput appears in every MC, as in the examples above, then 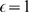 – in which case 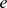 is said to have *full power* over 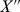. For the analysis of the SPN model below when 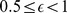, 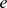 is a *high power* enput, when 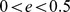 it is a *low power* enput, and when 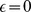 it is a *null power* enput. The larger the power of 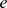, the more its perturbation is likely to disrupt convergence to the target pattern 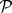. When 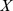 is too large, we estimate 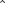 – similarly to the canalization measures discussed in the previous subsection.

**Figure 18:**
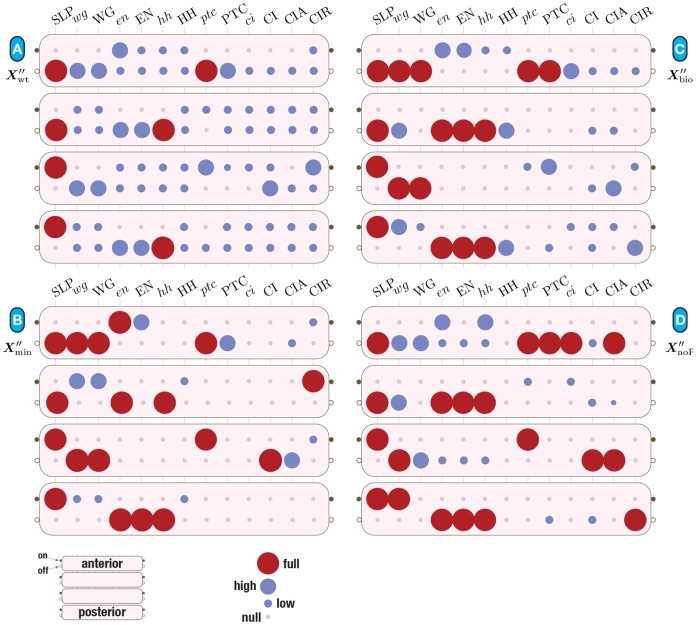
Enput power in the wild-type basin of attraction of the spatial SPN model. Enput power is shown for each of the four sets of MCs considered in our analysis: (A) 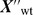, (B) 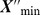, (C) 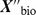 and (D) 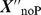. A parasegment is represented by four rounded rectangles, one for each cell, where the anterior cell is at the top, and posterior at the bottom. Since enput power is computed for every node in each of its two possible states, every cell rectangle has two rows of circles. The bottom row (marked on the sides with a white circle on the outside) corresponds to enput power of the nodes when *off*, while the top row is the enput power when the same nodes are *on* (marked on the sides with a dark circle). Each circle inside a cell's rectangle corresponds to the power of a given enput in the corresponding subset of MCs identified by the letters A to D. Full power is highlighted in red, other values in blue and scaled, while null power is depicted using small grey circles. Full power occurs only for enputs that are present in every MC (and configurations) of the respective set, whereas null power identifies nodes that are never enputs in any MC – always irrelevant for the respective dynamical behaviour.

We studied the wild-type attractor basin of the spatial SPN model using the four MC sets of interest: 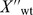, 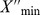, 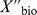, and 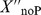 (see Minimal configurations subsection above) focusing on the power of literal enputs only. It is also possible to compute the enput power of group-invariant enputs. For example, the two-symbol MC 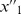 in Figure 17, has one of its four group-invariant enputs defined by 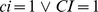. The power of this enput would tally those MCs in which this condition holds. Nonetheless, here we only measure the power of literal enputs and present the study of the power of group-invariant enputs elsewhere. The enput power computed for these four sets is depicted in Figure 18, where the output nodes PH and SMO are omitted because they are never input variables to any node in the SPN model, and therefore have null power. For the discussion of these results, it is useful to compare them to the known initial condition, 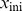 depicted in Figure 3, and the wild-type attractor, 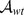 depicted in Figure 4 (a).

**Enput power in** 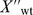 (see Figure 18A). The enputs with *full power* (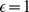) are: SLP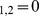, SLP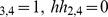 and 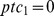. This is not entirely surprising since all of these genes and proteins are specified as such in both 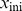 and 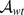. However, these values show that these enputs must remain in these states in the entire (sampled) wild-type basin of attraction. In other words, these enputs are *critical controllers* of the dynamics to the wild-type attractor. Indeed, the wild-type is not *robust* to changes in these enputs, which are likely to steer the dynamics to other attractors, as discussed further in the next section. Therefore, the spatial SPN model appears to be unable to recover the dynamic trajectory to the wild-type attractor when either the hedgehog gene is expressed in cells two and four; or the patched gene is expressed in the anterior cell, as well when the initial expression pattern of SLP determined upstream by the pair-rule gene family is disrupted in any way. There are also enputs with *high power* to control wild-type behaviour: 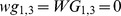, 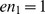, PTC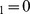, 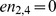, 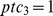, CI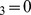 and CIR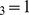. Again, these are the states of these genes and proteins in the known initial configuration of the SPN 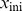, and most of them, except for 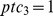, CI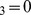 and CIR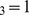 correspond to their final states in 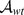.

In Figure 18A every node in the SPN – except the omitted nodes PH and SMO – appear as an enput, in at least one Boolean state, in many cases with very low values of . Thus, while macro-level dynamics is significantly canalized (see above), especially by SLP and the spatial signals for each cell, control of wild-type can derive from alternative strategies, whereby every node can act as an enput in some context. Nonetheless, most nodes ultimately do not observe much power to control wild-type behaviour, thus interventions to disturb wild-type behaviour are most effective via the few more powerful controllers (see also next section).

We can also compare the enput power computed for 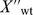 (Figure 18A), with the two-symbol MCs 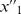 and 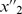 in Figure 17. These two MCs redescribe a significant portion of the wild-type attractor basin – 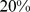 of our lower bound count of this basin. Because they only appear in 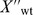 and not in any of the other MC sets we studied, the portion of the wild-type attractor basin they redescribe is unique to 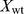, and can be analysed via 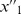 and 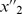. Most of the literal enputs specified in 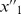 and 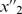 have high power in 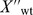, except for WG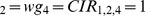, which are enputs in these two-symbol MCs that have low power. Conversely, there are literal enputs with high-power in 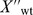 that are not enputs in these two-symbol MCs: EN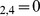 and PTC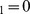. A key distinguishing feature of 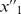 and 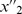 is the expression of CIR across the entire parasegment as well as of the wingless protein in the second cell, both of which are different from the trajectory between the known initial condition of the SPN and the wild-type attractor. Therefore, 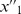 and 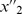 redescribe a (large) portion of the attractor basin outside of the more commonly studied dynamical trajectories.

**Enput power in** 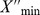 (see Figure 18B). We found an unexpected expression of CIR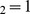 (now with full power) as well as 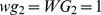 (high power). Other enputs whose expression is in opposition to both 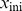 and 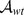 appear with low power: HH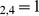 and CIR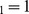. This again suggests that there is a substantial subset of the wild-type attractor basin, controlled by these and other enputs, distinct from the trajectory that results from the known (biologically plausible) initial configuration. We can also see that there is a significant number of nodes that do not play the role of enput in any MC – nodes with *null power*, depicted as small grey circles – as well as many more enputs with full power. 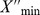 redescribes wild-type dynamics with the smallest number (23) of enputs; this set contains only 32 MCs out of the 1731 in 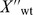. However, these are the most macro-canalizing MCs that guarantee convergence to wild-type. Indeed, because of their parsimony, they redescribe a very large subset of the wild-type attractor basin with at least 1.6 times more configurations than what was previously estimated for this basin (see above). Therefore, 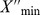 provides a solid baseline for the understanding of control in the wild-type attractor basin. This means that the genes and proteins with full power in this set are critical controllers of wild-type behaviour.

**Enput power in** 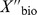 (see Figure 18C). Because this MC set only redescribes configurations in the dynamic trajectory from 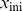 to 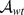, the transient dynamics observed in 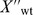 and 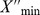, e.g. 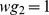 and CIR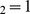, disappear. There are, however, other enputs with full power: 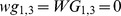, 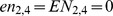, 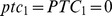. These critical enputs are particularly important for restricting analysis to a better-known portion of the wild-type attractor basin, for which the model was especially built.

**Enput power in** 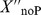 (see Figure 18D). This set of MCs is useful to understand the beginning of the segment polarity regulatory dynamics, with no proteins expressed. The set of critical genes that must be expressed (*on*) are 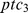 and 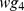, which appear with full power; moreover, 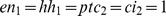 appear with high power. As shown in the figure, most other enputs with full or high power correspond to genes and proteins that must be inhibited (*off*), except, of course, SLP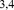 that are assumed to be always *on* in the SPN model.

We compared these results with previous work on identifying critical nodes in the SPN model. Chaves et al. [38] deduced, from the model's logic, minimal ‘pre-patterns’ for the initial configuration of the SPN that guarantee convergence to wild-type attractor. More specifically, two necessary conditions and one sufficient condition were deduced, which we now contrast with the enput power analysis.

The **first necessary condition** for convergence to the wild-type attractor is: 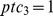, assuming that all proteins are unexpressed (*off*) initially, and the sloppy pair gene rule is maintained constant (i.e. SLP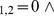 SLP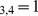.) Of the MC sets we analysed, only 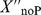 obeys the (biologically plausible) assumptions for this necessary condition. As we can see in Figure 18D, the enput 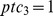 has full power on this MC set, which confirms this previous theoretical result. However, since every enput with full power is a necessary condition for the set of configurations described by its MC set, we can derive other necessary conditions for this set of configurations (with the same assumptions), such as 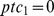, 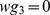, or 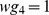 (see below). We can also see that not all assumptions for the first necessary condition are necessary; while the sloppy pair rule appears as four enputs with full power, not all proteins are required to be unexpressed: the expression of HH is irrelevant in every cell of the parasegment, as is the expression of PTC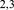, WG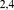, CIA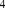, and CIR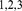. Moreover, the enput power analysis allows us to identify ‘degrees of necessity’; some enputs may not be necessary, but almost always necessary. This is the case of the expression of 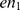, which has high power in 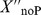, but is not a necessary condition as a few MCs can guarantee convergence to wild-type with 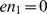 (which also appears as enput with low power). Naturally, if we relax the assumptions for condition 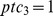, it may no longer be a necessary condition. This can be see when we look at the enput power analysis of the entire (sampled) wild-type basin 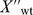 (Figure 18A) or the smaller 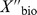 (Figure 18C). In these cases, which still preserve the sloppy pair rule assumption, 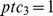 is no longer an enput with full power. This means that, according to this model, if some proteins are expressed initially, 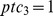 is no longer a necessary condition. Interestingly, we found that in the most macro-canalizing subset of the attractor basin, 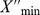 (Figure 18B) – which assumes the sloppy pair rule constraint but is not constrained to initially unexpressed proteins – 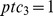 does appear as an enput with full power again. This means that in the most parsimonious means to control convergence to wild-type attractor, 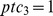 is a necessary condition too. It is noteworthy that in this case, not only can some proteins be expressed, but the expression of CIR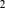 is also a necessary condition (enput with full power).

**Figure 19:**
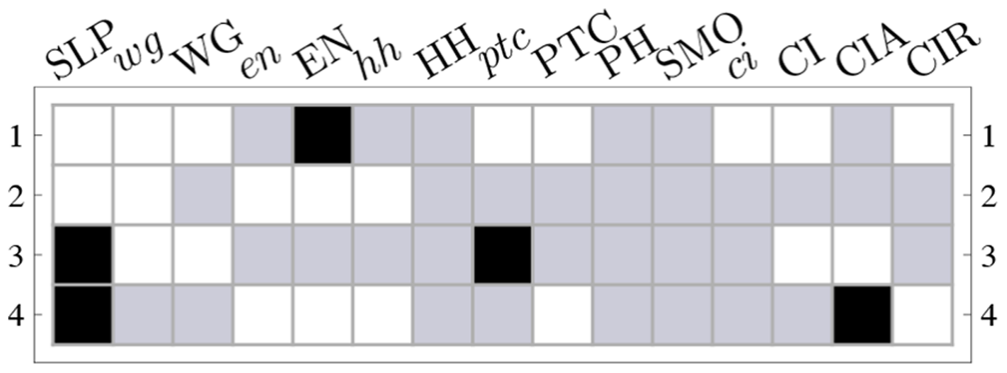
A MC not requiring 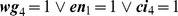 in wild-type attractor basin. When proteins are allowed to be expressed initially, the second necessary condition, reported in [38], ceases to be a necessary condition, as discussed in the main text; in the MC shown, 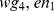 and 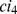 can be in any state and the network still converges to the wild-type attractor.

The **second necessary condition** for convergence to the wild-type attractor is: 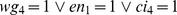, assuming that all proteins are unexpressed (*off*) initially, and the sloppy pair gene rule is maintained constant (i.e. SLP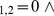 SLP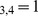) [38]. Again, only 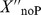 obeys the (biologically likely) assumptions for this necessary condition. As we can see in Figure 18D, the enput 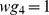 has full power, therefore it is a necessary condition. However, the enput 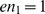 has high power, and the enput 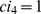 has no power. This means that they are not necessary, though 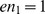 is most often needed. These results suggest that this necessary condition could be shortened to 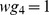, because in our sampling of the wild-type attractor basin, in the subset meeting the assumptions of the condition, we did not find a single configuration where 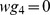. Even though our stochastic search was very large, it is possible that there may be configurations, with no proteins expressed, where 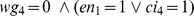, thus maintaining the original necessary condition. However, our enput power analysis gives a more realistic and nuanced picture of control in the SPN model under the same assumptions. While the necessary condition may be 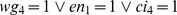, the individual enputs have strikingly different power in controlling for wild-type behaviour: 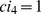 was never needed (no power), 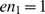 has high power, and 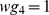 has full power. Naturally, if we relax the assumptions for this condition, it may no longer be a necessary condition. For instance, if we allow proteins to be expressed initially (still preserving the sloppy pair constraint), we can find MCs that redescribe configurations where 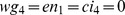. We found 171 MCs in 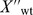 (available in *data S14* where this condition is not necessary, one of them depicted in Figure 19.

The **sufficient condition** for convergence to the wild-type attractor is: 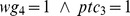, assuming that the sloppy pair gene rule is maintained constant (i.e. SLP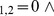 SLP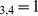). A variation of this sufficient condition assumes instead (maintaining the sloppy pair gene rule): 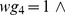 PTC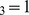 In their analysis, Chaves et al. [38] assume that all proteins are unexpressed and that many other genes are initially inhibited (*off*). Even though in Chaves et al. [38] the initial condition itself only requires 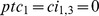, the argument hinges on propositions and facts that require knowing the state of additional genes such as 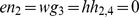. While Chaves et al. [38] concluded rightly from this minimal pre-pattern, that convergence to the wild-type pattern has a remarkable error correcting ability to expression *delays* in all other genes, the condition does not really describe robustness to *premature expression* of genes and proteins. It is interesting to investigate sufficient conditions that do require the states of most variables to be specified, giving us the ability to study robustness to both delays and premature expression of chemical species. The MC schemata we obtained with our macro-level analysis allows us to investigate such sufficient conditions directly.

**Figure 20:**
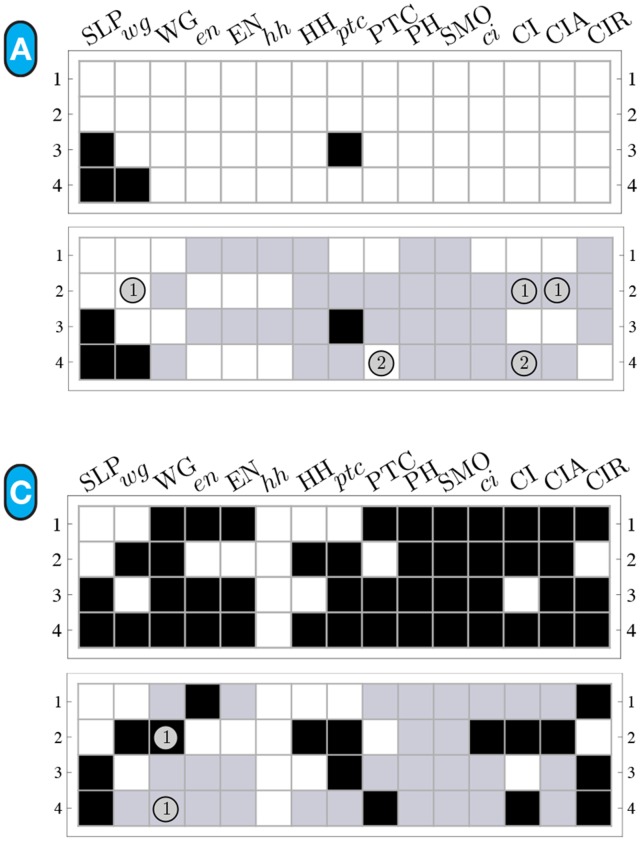
‘Extreme’ configurations converging to wild-type in the SPN model. (A) A configuration with the minimal number of nodes expressed that converges to wild-type, and its corresponding MC: 32 nodes are irrelevant, 24 must be unexpressed (*off*), and only 4 must be expressed (*on*). (B) The opposite extreme condition where 16 genes and proteins are unexpressed and all other 44 are expressed.

We searched the entire MC set 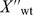 to retrieve the MCs with the *fewest* number of enputs specified as *on*. The 10 MCs (available in *S11*) we retrieved contain only 26 literal enputs, where in six MCs the two nodes in the sufficient condition above (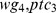), plus the nodes from the sloppy pair rule (SLP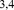) are *on*, 24 are *off* and the remaining 32 are wildcards, and thus irrelevant. In the remaining MCs, instead of 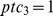, we found PTC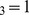 to be an enput. In those MCs 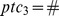. Converting all wildcards to *off* in one of these MCs, confirms the sufficient condition, as can be seen from Figure 20A, where SLP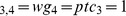, and everything else is *off*. This can be seen as an ‘extreme’ condition to wild-type attractor, with a minimum set of genes expressed. We also searched for the opposite extreme scenario, retrieving all MCs with the largest number of *on* nodes, that still converges to the wild-type pattern (available in *data S12*. By replacing all wildcards in such MCs to *on*, we obtained the configuration in which only 16 nodes must be inhibited (*off*), while the remaining 44 are expressed (*on*), depicted in Figure 20B. Interestingly, in this extreme configuration, 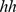 must remain *off* across the whole parasegment.

### Robustness to Enput Disruption

The power measure introduced in the previous subsection allows us to predict critical nodes in controlling network dynamics to a pattern of interest 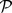. A natural next step is to investigate what happens when the critical controllers are actually disrupted. We can disrupt an enput 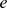 in an MC set with a variety of dynamic regimes. Here, we adopt the approach proposed by Helikar *et* *al.* [64], where a node of interest flips its state at time 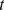 with a probability 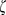, which can be seen to represent noise in regulatory and signalling events, as well as the ‘concentration’ of a gene (its corresponding mRNA) or protein – thus making it possible to use Boolean networks to study continuous changes in concentration of biochemical systems (see [64]).

We start from an initial set of configurations of interest: 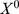. This can be a single configuration, such as the known initial configuration of the SPN 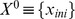 (as in Figure 3A), where the enput 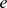 is in a specific (Boolean) value. Next, we set the value of *noise* parameter 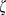, which is the probability that 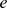 momentarily flips from its state in 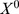 at time 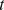. This noise is applied at every time step of the simulated dynamics; when a state-flip occurs at time 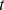, the node returns to its original state at 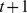 when noise with probability 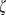 occurs again. Noise is applied to 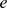 from 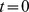 to 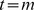. At time step 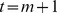 no more noise is applied to 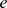 (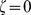) and the network is allowed to converge to an attractor. This process is repeated for 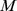 trials. Finally, we record the proportions of the 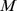 trials that converged to different attractors.

Since in this paper we only computed enput power for literal enputs (see previous subsection), we also only study literal enput disruption. It is straightforward to disrupt group-invariant enputs; for instance, the group-invariant enput defined by 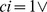 CI 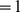 from the two-symbol MC 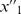 in Figure 17, can be perturbed by making 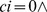 CI 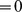. Nonetheless, for simplicity, we present the study of the disruption of group-invariant enputs elsewhere.

The enput power analysis in the previous subsection, revealed that in the wild-type attractor basin (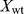) of the spatial SPN model there are the following critical nodes (or key controllers): across the parasegment, SLP proteins must be inhibited in cells 1 and 2 (SLP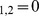) and expressed in cells 3 and 4 (SLP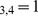), as determined by the pair-rule gene family; hedgehog genes (spatial signals) in cells 2 and 4 must be inhibited (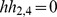); the patched gene in the anterior cell must also be inhibited (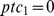). With the *stochastic intervention* procedure just described, we seek to answer two questions about these key controllers: (1) how sensitive are they to varying degrees of stochastic noise? and (2) which and how many other attractors become reachable when they are disrupted? In addition to the seven full power enputs, for comparison purposes, we also test the low power enput CI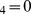. In the original SPN model the states of SLP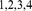 are fixed (the sloppy gene constraints). Because these naturally become enputs with full power (see Figure 18), it is relevant to include them in this study of enput disruption. However, by relaxing the fixed-state constraint on SLP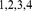, by inducing stochastic noise, the dynamical landscape of the spatial SPN model is enlarged from 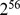 to 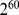 configurations. This means that more attractors than the ten identified for the SPN Boolean model (depicted in Figure 4) are possible, and indeed found as explained below.

**Figure 21:**
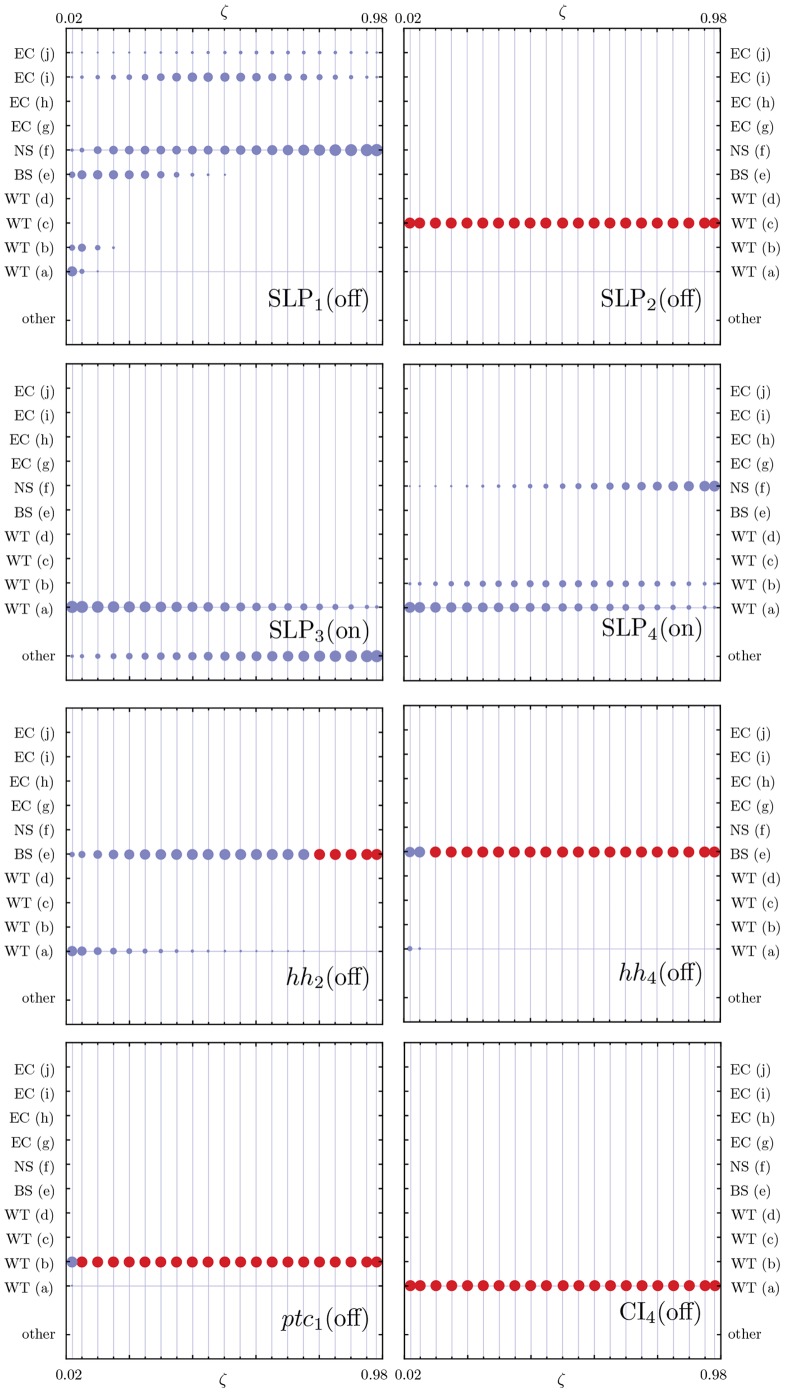
Wild-type enput disruption in the SPN model. Each coordinate 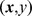 in a given diagram (each corresponding to a tested enput) contains a circle, depicting the proportion of trials that converged to attractor 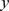 when noise level 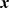 was used. Red circles mean that all trajectories tested converged to 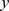.

We used 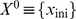 as the initial state of the networks analysed via stochastic interventions, because of its biological relevance. The simulations where performed with the following parameters: 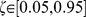, swept with 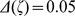, plus extremum values 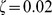 and 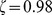; 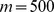 steps; 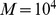. The simulation results are shown in Figure 21.

The first striking result is that disruption of SLP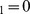 makes it possible to drive the dynamics away from wild-type into one of five other attractors (one of which a variant of wild-type). For 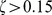 no further convergence to wild-type is observed, and at 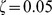 the proportion of trials that converged to wild-type was already very small. We also found phase transitions associated with the values of 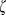. For 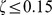 most trials converged to wild-type, wild-type (ptc mutant), broad-stripes or no-segmentation, and a very small proportion to two variants of the ectopic mutant. When 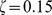 the proportion of trials converging to broad-stripes reaches its peak, and decreases, so that no trial converged to this mutant expression pattern for 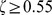. Finally, for 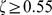 convergence to the ectopic variants reaches its peak and decreases steadily but does not disappear, while convergence to the no-segmentation mutant increases becoming almost 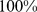 when 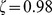. We thus conclude that SLP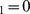 is a wild-type attractor enput which is very sensitive to noise.

In the case of SLP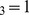, we observed convergence to an attractor that is not any of the original ten attractors – characterized by having two engrailed bands in cells 1 and 3 (see Data *S5*). The proportion of trials converging to wild-type and to the new attractor decrease and increase respectively, reaching similar proportions when 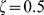. When 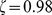, almost every trial converged to the new attractor. We conclude that SLP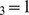 is a wild-type attractor enput whose robustness is proportional to noise.

Disruption of SLP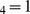 resulted in a behaviour similar to SLP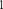, but with fewer possible attractors reached. As 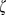 is increased, fewer trials converge to wild-type and growing proportions of trials converge to the wild-type 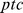 mutant pattern (reaching a peak at 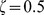) and the no-segmentation mutant. For more extreme values of 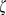, the majority of trials converged to the no-segmentation mutant. However, an important difference with respect to SLP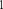 was observed: for 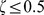 the majority of trials converged to wild-type, and convergence to this attractor is observed for the whole range of 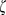. Thus the wild-type phenotype in the SPN model is much more robust to perturbations to the expression of SLP in the posterior cell (SLP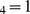), than to perturbations to its inhibition in the anterior cell (SLP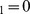).

With the parameters chosen, the disruption of SLP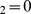 leads to a remarkable similar behaviour: any disruption (any amount of noise) leads to the same wild-type variant attractor pattern with two wingless stripes (c). Therefore, SLP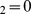 is not robust at all – though the resulting attractor is always the same and a variant of wild-type. In this case, convergence to a single attractor for all values of 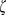 is the result of setting 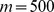 in our experiments. When we lower the value of 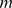 enough in our simulations, for low values of 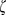, there are trials that are not perturbed and thus maintain convergence to the wild-type attractor. But any perturbation of SLP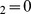 that occurs leads the dynamics to the wild-type variant.

Disruption of 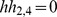 increasingly drives dynamics to the broad-stripes mutant. However, disruption of 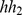 reveals greater robustness since a large number of trials still converges to wild-type for 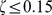, and residual convergence to wild-type is observed up to 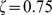. In contrast, any disruption of 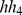 above 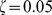 leads to the broad-stripes mutant, and even very small amounts of disruption lead to a large proportion of mutants. Similarly, disruption of 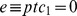 drives the dynamics to one – and the same – of the wild-type variants. Yet, when 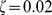 there is a minute proportion of trajectories that still converge to the wild-type attractor. Therefore, as expected, the wild-type attractor in the SPN model is not very robust to disruptions of the enputs with full power. Finally, and in contrast, no disruption of low-power enput CI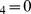 is capable of altering convergence to the wild-type attractor.

## Discussion

We introduced wildcard and two-symbol redescription as a means to characterize the control logic of the automata used to model networks of biochemical regulation and signalling. We do this by generalizing the concept of *canalization*, which becomes synonymous with redundancy in the logic of automata. The two-symbol schemata we propose capture two forms of logical redundancy, and therefore of canalization: input redundancy and symmetry. This allowed us to provide a straightforward way to *quantify* canalization of individual automata (micro-level), and to integrate the entire canalizing logic of an automata network into the Dynamics Canalization Map (DCM). A great merit of the DCM is that it allows us to make inferences about collective (macro-level) dynamics of networks from the micro-level canalizing logic of individual automata – with incomplete information. This is important because even medium-sized automata models of biochemical regulation lead to dynamical landscapes that are too large to compute. In contrast, the DCM scales linearly with number of automata – and schema redescription, based on computation of prime implicants – is easy to compute for individual automata with the number of inputs typically used in the literature.

With this methodology, we are thus providing a method to link micro- to macro-level dynamics – a crux of complexity. Indeed, in this paper we showed how to uncover *dynamical modularity*: separable building blocks of macro-level dynamics. This an entirely distinct concept from community structure in networks, and allows us to study complex networks with node dynamics – rather than just their connectivity structure. The identification of such modules in the dynamics of networks is entirely novel and provides insight as to how the collective dynamics of biochemical networks uses these building blocks to produce its phenotypic behaviour – towards the goal of explaining how biochemical networks ‘compute’.

By basing our methodology on the redescription of individual automata (micro-level), we also avoid the scaling problems faced by previous schemata approaches which focused solely on redescription of the dynamical landscape (macro-level) of networks [52]. By implementing the DCM as a threshold network, we show that we can compute the dynamical behaviour of the original automata network from information about the state of just a few network nodes (partial information). In its original formulation, the dynamic unfolding of an automata network cannot be computed unless an initial state of all its nodes is specified. In turn, this allows us to search for minimal conditions (MCs) that guarantee convergence to an attractor of interest. Not only are MCs important to understand how to *control* complex network dynamics, but they also allow us to *quantify macro-level canalization* therein. From this, we get a measurable understanding of the robustness of attractors of interest – the greater the canalization, the greater the robustness to random perturbations – and, conversely, the identification of *critical node-states* (enputs) in the network dynamics to those attractors. We provided a measure of the capacity of these critical nodes to control convergence to an attractor of interest (enput power), and studied their robustness to disruptions. By quantifying the ability of individual nodes to control attractor behaviour, we can obtain a testable understanding of macro-level canalization in the analysed biochemical network. Indeed, we can uncover how robust phenotypic traits are (e.g. robustness of the wild-type attractor), and which critical nodes must be acted upon in order to disrupt phenotypic behaviour.

We exemplified our methodology with the well-known segment polarity network model (in both the single-cell and the spatial versions). Because this model has been extensively studied, we use it to show that our analysis does not contradict any previous findings. However, our analysis also allowed us to gain new knowledge about its behaviour. From a better understanding of the size of its wild-type attractor basin (larger than previously thought) to uncovering new minimal conditions and critical nodes that control wild-type behaviour. We also fully quantified micro- and macro-level canalization in the model, and provided a complete map of its canalization logic including dynamical modularity. Naturally, our results pertain to this model; we do not claim that our results characterize the real Drosophila segment polarity gene network. However, our results, should they be found to deviate from organism studies, can certainly be used to improve the current model, and thus improve our understanding of Drosophila development. Thus a key use of our methodology in systems biology should be to help improve modelling accuracy. With the methodology now tested on this model, in subsequent work we will apply it to several automata network models of biochemical regulation and signalling available in the systems biology literature.

The pathway modules we derived by inspection of the DCM for the segment polarity network revealed a number of properties of complex networks dynamics that deserve further study. For instance, the dynamical sequence that occurs once each such module is activated is independent of the temporal update scheme utilized. Therefore, if the dynamics of a network is captured exclusively by such modules, its intra-module behaviour will be similar for both synchronous and asynchronous updating – denoting a particular form of robustness to timing. We will explore this property in future work, but as we showed here, the dynamics of the single-cell version of the SPN model is very (though not fully) controlled by only two pathway modules. This explains why its dynamical behaviour is quite robust to timing events as previously reported [38].

Research in cellular processes has provided a huge amount of genomic, proteomic, and metabolomics data used to characterize networks of biochemical reactions. All this information opens the possibility of understanding complex regulation of intra- and inter-cellular processes in time and space. However, this possibility is not yet realized because we do not understand the dynamical constraints that arise at the phenome (macro-) level from micro-level interactions. One essential step towards reaching these ambitious goals is to identify and understand the loci of control in the dynamics of complex networks that make up living cells. Towards this goal, we developed the new methodology presented in this paper. Our methodology is applicable to any complex network that can be modelled using binary state automata – and easily extensible to multiple-state automata. We currently focus only on biochemical regulation with the goal of understanding the possible mechanisms of collective information processing that may be at work in orchestrating cellular activity.

## Supporting Information

Data S1 **Glossary and mathematical notation.**
(PDF)

Data S2 **Details about the computation of wildcard and two-symbol schemata.**
(PDF)

Data S3 **Details about the conversion of schemata into a single threshold network.**
(PDF)

Data S4 **Algorithms for the computation of minimal configurations.**
(PDF)

Data S5 **Further details concerning the minimal configurations found for the segment polarity network model.**
(PDF)

Data S6 **Basic notions of the inclusion/exclusion principle.**
(PDF)

Data S7 **Minimal configurations for the segment polarity network model obtained from biologically-plausible seed configurations.**
(CSV)

Data S8 **Entire set of minimal configurations obtained for the segment polarity network model.**
(CSV)

Data S9 **Minimal configurations for the segment polarity network where no protein is** ***on*** **.**
(CSV)

Data S10 **Minimal configurations for the segment polarity network with the smallest number of nodes that need to be specified in a Boolean state.**
(CSV)

Data S11 **Minimal configurations for the segment polarity network with the fewest number of** ***on*** **nodes.**
(CSV)

Data S12 **Minimal configurations for the segment polarity network with the largest number of** ***on*** **nodes.**
(CSV)

Data S13 **(Wildcard) minimal configurations for the segment polarity network that were redescribed as two-symbol schemata.**
(CSV)

Data S14
**Minimal configurations for the segment polarity network that do not satisfy** 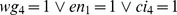 **.**
(CSV)
