# Supplementary material for: Canalization and Control in Automata Networks: Body Segmentation in Drosophila melanogaster
Source: PLoS One. 2013 Mar 8;8(3):e55946. doi: 10.1371/journal.pone.0055946 (PMC3592869; doi:10.1371/journal.pone.0055946)
Supplement: Data S1 — Glossary and mathematical notation. (PDF) [file pone.0055946.s001.pdf]

1 Notation

Symbol	Name	Context	Description
x	Boolean Automaton	Automata	Binary-state automaton
k	Number of inputs of x	Automata	How many inputs determine the transitions of an automaton x
F	Look-up table (LUT) of x	Automata	The transition function of x represented as a LUT (2^k entries).
f_α	LUT entry in F	Automata	k-tuple combination of input states i.e. *condition* and corresponding *transition*
c_α	condition part in a LUT entry f_α	Automata	k-tuple combination of input states i.e. *condition*
s_α	transition in a LUT entry f_α	Automata	Boolean state prescribed as the transition in f_α
$\mathcal{B}$	Boolean network	Networks	A graph of N automata with directed edges (source node is input of end node)
X	set of automata in $\mathcal{B}$	Networks	set of Boolean automata that constitute a BN $\mathcal{B}$
n	number of nodes in $\mathcal{B}$	Networks	$n = \|X\|$
$\boldsymbol{x}$	network configuration	Networks	collection of the states of all nodes in a BN $\mathcal{B}$
X_i	set of inputs of node x_i in $\mathcal{B}$	Networks	Set of input nodes of x_i
k_i	in-degree of x_i	Networks	Cardinality of X_i
F_i	Look-up table of x_i	Networks	Transition function represented as a LUT
$f_{i:\alpha}$	LUT entry in F_i	Networks	Sub-indices i and α, separated by ':', are used to specify node and entry.
$\mathcal{A}_i$	An attractor of $\mathcal{B}$	Networks	A specific (index i) fixed-point or periodic attractor of a BN $\mathcal{B}$.
$\sigma(\boldsymbol{x}) \rightsquigarrow \mathcal{A}$	Dynamic trajectory of $\boldsymbol{x}$ to $\mathcal{A}$	Networks	This notation is used to represent that the trajectory of some configuration $\boldsymbol{x}$ is known to converge to $\mathcal{A}$
$\#$	Wildcard symbol	Wildcard	If this symbol appears in a condition, the variable it represents can be in any state.
F'	wildcard-schema redescription of F	Wildcard	LUT where entries are wildcard schemata
f'_υ	a wildcard schema in F'	Wildcard	An entry in F' is like an entry in F but its condition part can have wildcard symbols.
Υ_υ	Entries $f_\alpha \in F$ in f'_υ	Wildcard	The set of original LUT entries in F redescribed by a single wildcard schema $\Upsilon_\upsilon \equiv \{f_\alpha : f_\alpha \rightarrowtail f'_\upsilon\}$
$\circ_m$	Position-free symbol	2-Symbol	If a variable in the condition part of a schema is marked with this symbol, it can exchange places with any other variable in the same schema marked with the same symbol. Index m used to distinguish subsets of identically-marked inputs
β	Depth of search for two-symbol schemata	2-Symbol	Defines the minimum number of wildcard schemata in a two-symbol redescription.
F''	2-symbol redescription of F	2-Symbol	LUT where entries are two-symbol schemata
f''_θ	a 2-symbol schema in F''	2-Symbol	An entry in F'' is like an entry in F but its condition part can have wildcard and position-free symbols.
Θ_θ	Entries $f_\alpha \in F : f_\alpha \rightarrowtail f''_\theta$	2-Symbol	The set of original LUT entries in F redescribed by a single 2-symbol schema $\Theta_\theta \equiv \{f_\alpha : f_\alpha \rightarrowtail f''_\theta\}$
Θ'_θ	Schemata $f'_\upsilon \in F' : f'_\upsilon \rightarrowtail f''_\theta$	2-Symbol	The set of wildcard schemata in F' redescribed by a single 2-symbol schema $\Theta'_\theta \equiv \{f'_\upsilon : f_\upsilon \rightarrowtail f''_\theta\}$
X_ℓ	set of literal enputs in a schema f''	2-Symbol	The variables in the condition part of schema f'' that are specified in a Boolean state (not wildcard)
η_ℓ	size of literal-enput set in a schema f''	2-Symbol	$n_\ell = \|X_\ell\|$
X^s_ℓ	state-s literal enputs in f''	2-Symbol	Subset $X^s_\ell \subset X_\ell$ of literal enputs in a specific state $s : s \in \{0,1\}$
X_g	group-invariant enput in a schema f''	2-Symbol	The set variables in the condition part of schema f'' that are marked with an identical position-free symbol, in every state they can take
X^s_g	elements of X_g in state s	2-Symbol	This notation is used to refer to the members of a group-invariant enput instantiated in a specific state s, that is $X^s_g = \{\forall x_i \in X_g \wedge x_i = s\}$
η	number of group-invariant enputs in f''	2-Symbol	Number of subsets of inputs marked with a distinct position-free symbol
n_g	size of a single group-invariant enput g in f''	2-Symbol	Number of inputs marked with the position-free symbol in g.
n^s_g	a sub-constraint in X_g on state $s \in \{0,1\}$	2-Symbol	specifies a group-invariant constraint in the set X_g, at least n^s_g variables must be in state s

Symbol	Name	Context	Description
τ	Threshold of a t-unit	Canalizing Maps	The firing activity threshold of a transition unit in the canalizing map of an automaton x
$\underline{k}_{\rm e}(x)$	lower-bound effective connectivity of x	Automata control	Smallest number of inputs that, on average, determine the transition of x when the states of all its inputs are equi-probable
$\overline{k}_{\rm e}(x)$	upper-bound effective connectivity of x	Automata control	Maximum number of inputs that, on average, are needed to determine the transition of x when the states of all its inputs are equi-probable
$k_{\rm r}(x)$	input redundancy in x	Automata control	$k_{\rm r}(x) = k(x) - k_{\rm e}(x)$
$\underline{k}_{\rm s}(x)$	lower-bound input symmetry of x	Automata control	Smallest number of inputs with with, on average, an input can 'switch places with' to determine the transition of x when the states of all its inputs are equi-probable
$\overline{k}_{\rm s}(x)$	upper-bound input symmetry of x	Automata control	Largest number of inputs with with, on average, an input can 'switch places with' to determine the transition of x when the states of all its inputs are equi-probable
$\hat{\boldsymbol{x}}$	partial configuration	dynamic unfolding	A configuration of the BN where a subset of nodes is specified (in a Boolean state) while other nodes are *unknown*
$\sigma(\hat{\boldsymbol{x}}) \rightsquigarrow \mathcal{P}$	dynamics from $\hat{\boldsymbol{x}}$ leads to outcome $\mathcal{P}$	dynamic unfolding	Dynamic trajectory from $\hat{\boldsymbol{x}}$ ends in outcome pattern $\mathcal{P}$ (which can be a full attractor, or a partially specified steady-state configuration).
$\boldsymbol{\mathcal{P}}$	target outcome	Minimal Configs.	A target pattern comprises any configurations that share some property of interest. It can contain a single attractor, or a set of outcome patterns.
$\boldsymbol{x}'$	minimal configuration	Minimal Configs.	A configuration of the BN where a subset of nodes is specified (in a Boolean state) while other nodes are *unknown* such that $\sigma(\boldsymbol{x}') \rightsquigarrow \mathcal{P}$
$\|\boldsymbol{X}'\|$ or $\|\boldsymbol{X}''\|$	Number of configurations $\boldsymbol{X}$ redescribed by a set of MCs	Minimal Configs.	The cardinality $\|\boldsymbol{X}\|$ of the set of configurations redescribed by a set of MCs may be counted exactly, or sampled.
$\sigma(\boldsymbol{x}') \rightsquigarrow \boldsymbol{\mathcal{P}}$	input-output relationship between a MC $\boldsymbol{x}'$ and the target pattern it unfolds to, $\boldsymbol{\mathcal{P}}$	Minimal Configs.	Dynamic trajectory of a minimal configuration $\boldsymbol{x}'$ ends in target pattern $\boldsymbol{\mathcal{P}}$ (this can be a single (full or partial) configuration, or a set of these that defines a pattern).
