# Supplementary material for: Canalization and Control in Automata Networks: Body Segmentation in Drosophila melanogaster
Source: PLoS One. 2013 Mar 8;8(3):e55946. doi: 10.1371/journal.pone.0055946 (PMC3592869; doi:10.1371/journal.pone.0055946)
Supplement: Data S2 — Details about the computation of wildcard and two-symbol schemata. (PDF) [file pone.0055946.s002.pdf]

2 Computation of Wildcard and Two-Symbol Schemata

For the computation of wildcard schemata we use espresso [1], available for download at http://embedded.eecs.berkeley.edu/pubs/downloads/espresso/index.htm/. This implementation contains an option for computing all the prime implicants for a set of LUT entries, typically those with transition to on, thus providing an efficient implementation for the computation of the Blake's normal form of the input LUT F. We compute the prime implicants for the subsets of entries of F with each possible transition separately, to obtain the complete set of wildcard schemata F'.

For the computation of two-symbol schemata for given set F' we use the functions fgi and g (below), based on the work of McCluskey on detecting of symmetry groups [2]. The algorithm takes as input the set F' after it has been pre-processed as follows: First, the set is partitioned into subsets where each contains schemata with identical transition. For Boolean schemata this means separating schemata with transitions to on in one partition, and schemata with transitions to off in another. Then each of these partitions is partitioned again into subsets of schemata the condition parts of which have the same counts of 0s, 1s and wildcards (and of any number of other states if $k > 2$). Here we focus on the Boolean case, and thus counts are a triplet of values with the counts of 0s, 1s and wildcards (in that order). For simplicity, we use the term 'schema counts' to refer to the number of 0s, 1s and $\#s$ in the condition part of a schema, or 'variable counts' to refer to the counts for a given variable in a set of wildcard schemata – that is, the counts in the column of the matrix that contains the condition parts of a set of schemata F', denoted in the algorithm by $A_{m\times k}$ (m schemata and k input variables). Refer to the set containing all these partitions as P, which is the input provided to the function find group-invariant enputs fgi(P), below.

The function checkCounts(s) takes a list of variable counts, where the position of each corresponds to a column of A and (1) removes counts that correspond to columns in which states are fixed (all wildcards, or a possible literal enput); (2) for each of the remaining counts (not fixed) that may correspond to variables in a group-invariant enput, its position (column index) along with the count itself are all gathered in a set. This set is partitioned into subsets where each contains the variables (column indexes) with idential variable counts. If all of these sets contain at least two columns (indexes) then the set of these is returned as the variable sv, otherwise sv is returned with the value -1, meaning that A cannot be a symmetric group. The function checkValidSpec(A, sv) (returns $True$ or $False$) checks that the input variables in every subset of colums of A with identical, non-fixed, variable counts (specified in sv) have the same schema counts. This is another condition for A to be a possible symmetric group. The function canSwap(A, i, j) simply permutes the ith and j^{th} columns of A and rearranges the rows of the resulting matrix. If this resulting matrix is identical to A the function returns $True$, and returns $False$ otherwise. The pseudocode for fgi and g is provided below, as well as a worked example in the following subsection.

For partitions $H' \in P : |H'| > 20$ the size of the search space for symmetric groups becomes significantly large. For example, if in such partition H' there are only symmetric groups containing e.g. two wildcard schemata, the algorithm has to search on approximately $2^{|H'|}$ subsets of H'. For example, if $|H'| = 20$ and there are no symmetric groups in it, the algorithm will evaluate approximately 10^7 of its subsets before it fails. If $|H'| = 30$ and it does not contain symmetric groups the algorithm will evaluate approximately 1.6×10^{10} subsets – a search space that is too large for feasible computation. However, it is important to emphasize that $|H'| \approx 20$ is not a strict limit: if a very large set H' contains large symetric groups, then the algorithm can identify it after just relatively few expansions of the search space. Therefore, when a given set P contains large partitions H' it is possible to limit the expansion of the search space. By default, the algorithm ends when all partitions $H' \in P$ have cardinality $|H'| = 1$, which means the entire search space has been expanded.

Algorithm 1 Function fgi(P)

```
F'' = ∅ { the output two-symbol schemata will be stored here}
map the transition for every partion in P onto a list S
map the condition parts of each partition in P onto P
while P ≠ ∅ do
    H' = head(P)
    P = tail(P) {here head of list is processed, thus we remove it from P}
    colcnts = ∅
    for j = 1 to j = k do
        colcnts = append(colcnts, counts(H'[_, j])) {counts of 0s, 1s, and #s for each column of H'}
    end for
    sv = checkCounts(colcnts)
    if sv ≠ −1 then
        if checkValidSpec(H') then
            sw = true
            GI = sv
        end if
    else
        sw = False
    end if
    if sw ∧ (H', GI) ⊈ F'' then
        r = g(H', GI)
        if r ≠ ∅ then
            F'' = F'' ∪ (H', r) {H' with its group-invariant enputs}
        end if
    else
        if m > 2 then
            find C' ⊂ H' : |C'| = m − 1
            P = P ∪ C' {expanded search tree}
        else
            do nothing, H' was the head of P (removed at the start of the while loop)
        end if
    end if
end while
```

Algorithm 2 Function g(H', L)

H' {input: a partition of F' (condition parts only) with identical schema counts states$\{0, 1, .., k\}$}
L {input: list of columns (variables) of H' with identical (not fixed) var. counts}
$G_t = \emptyset$
for $s = 1$ *to* $|L|$ do
  $sofar = \emptyset$
  for $i = L[s, 1]$ *to* $i = |L[s]|$ do
    $G_c = \{i\}$
    if $i \notin sofar$ then
      $sofar = sofar \cup \{i\}$
      for $j = L[s, 2]$ *to* $j = |L[s]|$ do
        if $j \notin sofar \wedge canSwap(H', i, j)$ then
          $G_c = G_c \cup \{j\}$
          $sofar = sofar \cup \{j\}$
        end if
      end for
      if $|G_c| \neq 1$ then
        $G_t = G_t \cup G_c$
      end if
    end if
    if $\bigcup G_t = \bigcup L$ then
      $returnG_t$ {all variables in L are part of a group invariant enput}
    else
      return$\emptyset$
    end if
  end for
end for

References

1. Rudell R, Sangiovanni-Vincentelli A (1987) Multiple-valued minimization for pla optimization. IEEE Transactions on Computer-Aided Design 6: 727-751.
2. McCluskey E (1956) Detection of group invariance or total symmetry of a boolean function. Bell System Technical Journal 35: 1445-1453.

2.1 Worked Example

Assume the algorithm fgi is called with a set of partitions P containing the following single partition

$$P = \left\{ \begin{pmatrix} 1 & \# & \# & 0 & \# & : & 0 \\ \# & 1 & \# & 0 & \# & : & 0 \\ 1 & \# & \# & \# & 0 & : & 0 \\ \# & 1 & \# & \# & 0 & : & 0 \\ \# & \# & \# & 1 & 0 & : & 0 \end{pmatrix} \right\}$$

In step (2) the output variable F'' is initialized as an empty set. After steps (3) and (4) we obtain a new P that contains only the condition parts of each original partion in P; the common transition for each partition in P is stored in S:

$$P = \left\{ \begin{pmatrix} 1 & \# & \# & 0 & \# \\ \# & 1 & \# & 0 & \# \\ 1 & \# & \# & \# & 0 \\ \# & 1 & \# & \# & 0 \\ \# & \# & \# & 1 & 0 \end{pmatrix} \right\}$$

$$S = \{0\}$$

The while loop is entered on step (5) after checking that $P \neq \emptyset$. In step (6) A is assigned the first partition in P (head of P)

$$A = \begin{pmatrix} 1 & \# & \# & 0 & \# \\ \# & 1 & \# & 0 & \# \\ 1 & \# & \# & \# & 0 \\ \# & 1 & \# & \# & 0 \\ \# & \# & \# & 1 & 0 \end{pmatrix}$$

In step (7) P is assigned the rest of the list P, which means $P = \emptyset$. In steps (8-10) the variable colcnts is assigned the counts of 0s, 1s and # in the columns of A:

$$colcnts = \{\{0,2,3\}, \{0,2,3\}, \{0,0,5\}, \{2,1,2\}, \{3,0,2\}\}$$

And with this variable the function checkCounts is called. The function removes the count $\{0,0,5\}$ because it corresponds to a fixed column of A and records the how many of the remaining columns have the same count. Since there are two counts unique to single columns $\{2,1,2\}$ and $\{3,0,2\}$, the function returns -1, and this means that A is not a candidate for a symmetric group under permutation. The algoritm then checks this value at step (12) and assigns the Boolean value 'False' to sw in step (17). Since $sw = False$ at step (18) the algorithm jumps to step (23) in which A is 'split', and the search space for symmetric groups expanded. This is done at step (22), where if A contains $m > 2$ schemata, then (in step 23) find all the distinct subsets of A that contain $m - 1$ schemata and append the result to P (step 24). For the current example, five subsets of A are now in P. With the new P the algorithm returns to the start of the main While loop. The head of P is now:

$$A = \begin{pmatrix} 1 & \# & \# & 0 & \# \\ 1 & \# & \# & \# & 0 \\ \# & 1 & \# & 0 & \# \\ \# & 1 & \# & \# & 0 \end{pmatrix}$$

At step (10) the variable colcnts is update to $colcnts = \{\{0,2,2\}, \{0,2,2\}, \{0,0,4\}, \{2,0,2\}, \{2,0,2\}\}$. checkCounts ignores the fixed wildcard column (variable 3) and identifies that for every distinct count there are at at

least two matching columns, returning a list of lists where each list corresponds to the indexes of columns of A that have the same variable counts: $sv = \{\{1, 2\}, \{4, 5\}\}$

At step (12) since $sw \neq -1$ the function checkValisSpec is called with the current A and sv. This function selects the columns in each element of sv and computes the schema counts, checking they are identical. For the first element of sv the corresponding columns of A are

$$A[\_, \{1, 2\}] = \begin{pmatrix} 1 & \# \\ 1 & \# \\ \# & 1 \\ \# & 1 \end{pmatrix}$$

All with schema counts $\{0, 1, 1\}$. For the second element of sv the corresponding columns of A are

$$A[\_, \{4, 5\}] = \begin{pmatrix} 0 & \# \\ \# & 0 \\ 0 & \# \\ \# & 0 \end{pmatrix}$$

All with schema counts $\{1, 0, 1\}$. Since for every element of sv the corresponding columns of A have identical schema counts, the function returns 'True'. At step (14) the variable $sw = True$; at step (15) $H = \{\{1, 2\}, \{4, 5\}\}$. At step (18) since $sw = True$ and the schemata in A are not all subsumed by any schema in F'' (it is currently empty), the function g is called with A and H. This function checks if A is indeed a symmetric group under permutation.

`Shifting control to function g, with inputs A and H`

This function iterates over each of the groups of columns that have the same variable counts, stored in H. For every list of columns in H the algorithm iteratively checks what pairs of columns can be exchanged leaving A unchanged. It is possible that the column indexes of a single element of H even though they have the same counts, cannot all be exchanged with each other. For example, it is possible that given one such list, e.g. $\{2, 3, 4, 5\}$ columns 2 (4) and 4 (2) can be exchanged, and columns 3 (5) and 5 (3) can be exhanged, but not any other pair. In such case, the element of H is broken down into two sublists that will be marked with different position-free symbols – provided A as a whole is a symmetric group. It is also possible that – continuing with the same example – three columns can exhange places with each other, but that a fourth one cannot exchange places with any other. In this case g would return the empty set, since A is not a symmetric group. In our core example, this function identifies that columns 1 and 2 as well as 4 and 5 can be exchanged, returning the value of H unchanged, which is assigned to the variable r. In summary, function g checks that for every list of columns of H every element can exachange places with at least one other column.

`Shifting control back to fgi`

At step (20) since r is not the empty set, the output variable F'' is updated, now containing a two-symbol schema represented as the tuple (A,r). After this the algorithm goes back to the start of the While loop, to continue checking the remaining four partitions in P. All of these fail to satisty the checkCounts criterion, and P is expanded each time to contain further subsets to search on. No other partition satisfies all the criteria needed and eventually P is the empty set, when the algorithm stops, returning the single two-symbol schema identified, with its transition in S (to 0), and including the the wildcard schema $\{\#, \#, \#, 1, 0, :, 0\}$ in the original P that was not redescribed into any two-symbol schema. For various subsets of size $m = 2$ the criterion to file is the one specified in step (18) of fgi where the algorithm checks whether the schemata in the current matrix A are all contained in at least one element of F''.
