# Supplementary material for: Canalization and Control in Automata Networks: Body Segmentation in Drosophila melanogaster
Source: PLoS One. 2013 Mar 8;8(3):e55946. doi: 10.1371/journal.pone.0055946 (PMC3592869; doi:10.1371/journal.pone.0055946)
Supplement: Data S3 — Details about the conversion of schemata into a single threshold network. (PDF) [file pone.0055946.s003.pdf]

3 Conversion to threshold network

The condition part of a schema f'' can be formulated as a conjunction of constraints in the form of Expression (1), here the general expression that accounts for one or more group invariant enputs. Recall that a schema's condition part contains literal enputs, group invariant enputs or both. The first two conjuncts of Expression (1) represent the literal-enput constraints. That is, the subset of inputs $X_\ell \subseteq X$ that must be observed at time t in state 0 and state 1. There are $|X_\ell|$ literal enputs in f'', and this quantity is denoted by n_ℓ. The last two conjuncts represent a single group-invariant enput g as permutation sub-constraints n_g^0, n_g^1 and $n_g^\#$ on the subset of inputs $X_g \subseteq X$ marked with the position-free symbol. Any sub-constraint on a state s must satisfy $n_g^s < n_g$ in a valid group-invariant enput (see main text). The instantiation of Expression (1) for a specific schema f'' only needs to include the conjuncts that are appropriate for representing the schema. For instance, if a schema does not have a group-invariant enput, but has literal enputs (in both Boolean states), the corresponding instantiation of Expression (1) includes only the first two conjuncts. Thus to meet the condition of f'' it is sufficient that the observed input states of the automaton at time t satisfy every constraint in the schema's instance of Expression (1). The inverse relation holds as well: if the condition of a schema f'' is met, the constraints specified in Expression (1) are all satisfied. This decomposition of the condition part of a schema f'' allows its conversion to the threshold network formalism introduced in the main text. The conversion of every schema $f'' \in F''$ for x is the automaton's canalizing map.

$$\bigwedge_{i_j \in X_\ell^0} \neg i_j \bigwedge_{i_j \in X_\ell^1} i_j \bigwedge_{g=1}^{\zeta} \left[\left(\sum_{i_j \in X_g} \neg i_j \geq n_g^0 \right) \wedge \left(\sum_{i_j \in X_g} i_j \geq n_g^1 \right) \right] \tag{1}$$

3.1 A first approach: direct constraint mapping

Before explaining the conversion method introduced in the main text, it is useful to consider an alternative – and very simple – method that allows a direct mapping of Expression (1) to a threshold network. In the main text, for simplicity, for this expression of the condition part of a schema we assumed the existence of only one group invariant enput. Here we present that same expression in its general form to account for any number η of group-invariant enputs. The obtained threshold-network representation has some important limitations, but it is useful as a conceptual bridge to the core redescription method explained in the following section. The procedure takes as input the instantiation of Expression (1) for a schema f'' and then instantiates and connects t-units and s-units as follows: (1) a single t-unit to account for all existing literal enputs, with all inputs $x_i \in X_\ell$ as its incoming s-units, linked with single fibres. Since all literal enputs are mandatory, the threshold of this t-unit is $\tau = n_\ell$; (2) a distinct t-unit for each sub-constraint specified in every group invariant enput, that is, if $\eta \geq 1$. The t-unit for a given sub-constraint n_g^s (on some state s) must have the n_g inputs $x_i \in X_g : x_i = s$ as incoming s-units, and its threshold is $\tau = n_g^s$; (3) finally, a 'consolidating' t-unit integrates the output signals from the previously instantiated t-units. The threshold τ of this t-unit equals the number of t-units previously instantiated for the schema. The general expression is $\tau = 1 + 2\zeta$, assuming there are literal enputs. See Figure 1 for an illustration of this simple procedure.

The resulting threshold network captures all the features of f'', but it has some important limitations. The first is that it is not a parsimonious representation: a minimum of two t-units is always needed to represent a schema. That is, the consolidating unit, plus the appropriate number of t-units needed to capture the constraints in f''. The second limitation is that – because of the need of a consolidating t-unit – this representation introduces an extra delay for every transition. The 'computation' performed by the t-units – each of which is assumed to take a standard one-step delay – requires that the different constraints in the schema are checked individually by a 'layer' of t-units, and then integrated by a consolidating t-unit. This means that the transition of the automaton in this threshold-network representation always takes two discrete time steps [1]. The method explained below

[1] For a more parsimonious representation it seems possible to remove the t-unit used to integrate the literal enputs, linking the s-units corresponding to these enputs directly to the consolidating t-unit, and increasing the threshold of the latter by $|X_\ell|$ instead

produces a threshold network representation that overcomes these two limitations: each schema is mapped to a single t-unit and therefore every transition takes one time step.

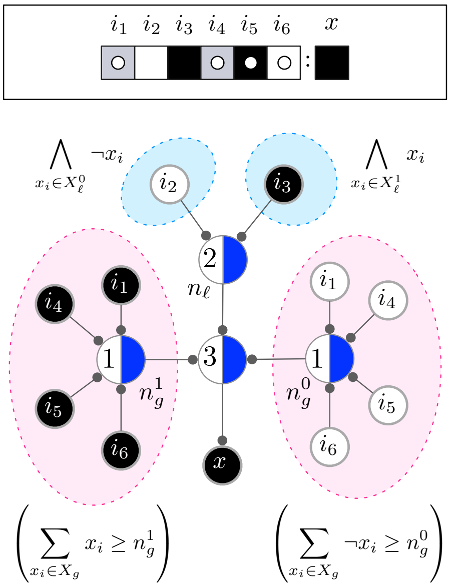

Figure 1: Simple procedure for the redescription of a schema f'' into a threshold network representation (with important limitations). In the exemplar schema shown in the figure (top) – the same example schema used in the main text – there are two literal enputs accounted for by the t-unit at the top with threshold $\tau = n_\ell = 2$. Since there is a group-invariant enput with permutation sub-constraints on both Boolean states, two extra t-units are instantiated. The t-unit on the left accounts for the permutation sub-constraint $n_g^1 = 1$. It thus has as incoming s-units the inputs $x_i \in X_g : x_i = 1$ and threshold $\tau = n_g^1 = 1$. In a similar way, the t-unit on the right accounts for the sub-constraint $n_g^0 = 1$. Notice that each of the conjuncts in Expression (1) are directly mapped onto the network representation.

3.2 The implemented approach: parsimonious representation, one t-unit per schema

The method for the conversion of a schema to a CM using branching and fusing wires was explained in the main text. The key aspects of this conversion are the wiring mechanism itself – explained in the text – and the computation of the t-unit's threshold τ, introduced in the main text, and explained further here.

'Branch-Fuse' approach to the integration of a group-invariant enput. The short rationale for this procedure is the following.

The satisfaction of a sub-constraint e.g. n_g^0 can be broken in two parts, one that assumes that a combination of $n_g^0 - 1$ input variables are off; the other concerns the remaining variables, namely $n_g - (n_g^0 - 1)$, amongst which at least one must be off so that the sub-constraint is satisfied.

Focusing on the latter part, that is, on the remaining $n_g - (n_g^0 - 1)$ variables, we can consider the set $\boldsymbol{S}$ of all subsets S_i that contain $n_g - (n_g^1 - 1)$ distinct s-units each, and where the s-units correspond to the variables in the group-invariant enput in state 0.

of by one. However, this resulting threshold network does not reflect the temporal properties of the constraints in the condition part of f''. That is, a transition takes one time step when f'' does not contain any group-invariant enputs. But if there are both literal and group-invariant enputs, the signals coming from the former would reach the consolidating t-unit one time step before the signals coming from the latter. In other words, literal and group-invariant enputs would be out of phase.

From the Pascal's combinatorial identity, we know that any s-unit is a member of

$$a = \binom{n_g - 1}{n_g^0 - 1}$$

subsets $S_i \in \boldsymbol{S}$.

Therefore, since every subset $S_i \in \boldsymbol{S}$ is represented in the CM by fusing branches taken from its constituent s-units, the firing of just one s-unit will increase the t-unit's threshold activity by a.

In the general case, if m s-units are firing, the firing activity of the t-unit is given by,

$$a = \sum_{i=1}^{m} \binom{n_g - i}{n_g^0 - i}$$

where $m \leq n_g$. This means that if $m = n_g^0$, there is at least one firing s-unit in every subset $S_i \in \boldsymbol{S}$, and the sum above can be thus simplified to,

$$a = \sum_{i=1}^{n_g^0} \binom{n_g - i}{n_g^0 - i} = \binom{n_g}{n_g^0 - 1} = |\boldsymbol{S}|$$

Hence a is the threshold increase for τ to satisfy the sub-constraint n_g^0. Finally, if there is more than one group-invariant enput, the procedure is repeated for the collection of all sub-constraints specified by them. Each subconstraint results in new fibres added, as well as on an increase of the threshold τ.

$$\tau = \tau_{\text{prev}} + \binom{n_g}{n_g - (n_g^s - 1)}$$

where τ_{prev} is the previous threshold for the t-unit in the case literal enputs and/or other group-invariant enput sub-constraints were previously integrated. This expression can be simplified obtaining,

$$\tau = \tau_{\text{prev}} + \binom{n_g}{n_g^s - 1}$$

The t-unit's threshold is therefore increased by the total number of fibre endings produced by the integration procedure of a single sub-constraint n_g^s just described. This threshold is only reached if at least n_g^s of the s-units for the inputs $x_i \in X_g : x_i = s$ are firing.

How and why does the 'branch and fuse' work? A mathematical way to reason about the integration procedure just introduced is to consider first that the total number of distinct combinations (subsets) of firing s-units for $x_i \in X_g^s$ that satisfy the sub-constraint n_g^s is given by,

$$\binom{n_g}{n_g^s} \tag{2}$$

which by the Pascal's combinatorial identity about binomial coefficients can be expanded as follows,

$$\binom{n_g}{n_g^s} = \binom{n_g - 1}{n_g^s} + \binom{n_g - 1}{n_g^s - 1} \text{ for } 1 \leq n_g^s \leq n_g \tag{3}$$

that is, the number of subsets containing n_g^s s-units for inputs $x_i \in X_g : x_i = s$ is the sum of the number of such subsets that do not contain some s-unit (x_i) plus the number of subsets that do contain it. If follows that the number of branches each s-unit needs for the integration of a sub-constraint in which the s-unit is involved is given by,

$$b = \binom{n_g - 1}{n_g^s - 1} \tag{4}$$

The final step in this reasoning is to show that the threshold increase for the t-unit is reached if and only if at least n_g^s s-units are firing. This can be expressed mathematically as follows,

$$\binom{n_g}{n_g^s - 1} = \sum_{e=1}^{n_g^s} \binom{n_g - e}{n_g^s - e} \tag{5}$$

proof. Using Pascal's identity we expand Equation (4), and then, recurrently, expand the last term on the RHS of resulting next expansion (k times)

$$\begin{aligned}
\binom{n_g}{n_g^s - 1} &= \binom{n_g - 1}{n_g^s - 1} + \binom{n_g - 1}{n_g^s - 2} \\
&= \binom{n_g - 1}{n_g^s - 1} + \binom{n_g - 2}{n_g^s - 2} + \binom{n_g - 2}{n_g^s - 3} \\
&= \binom{n_g - 1}{n_g^s - 1} + \binom{n_g - 2}{n_g^s - 2} + \ldots + \binom{n_g - (n_g^s - 1)}{n_g^s - (n_g^s - 1)} + \binom{n_g - n_g^s}{n_g^s - n_g^s} + \binom{n_g - n_g^s}{n_g^s - n_g^s - 1} \\
&= \binom{n_g - 1}{n_g^s - 1} + \binom{n_g - 2}{n_g^s - 2} + \ldots + \binom{n_g - (n_g^s - 1)}{n_g^s - (n_g^s - 1)} + 1 + 0
\end{aligned}$$

Therefore the original Equation (4) for τ can be rewritten as follows:

$$\binom{n_g}{n_g^s - 1} = \sum_{e=1}^{n_g^s} \binom{n_g - e}{n_g^s - e} \tag{6}$$

□

The procedure just outlined is repeated for each sub-constraint in g, namely n_g^0 and n_g^1 for Boolean automata. In the end, the final threshold for the t-unit representing f'' is the sum of the thresholds obtained from the integration of literal-enput constraints and group-invariant enput sub-constraints. The integration of every (sub) constraint in Expression (1) results in a number of single (or branching/fusing) fibres to the t-unit, the endings of which are all needed to reach the specific threshold increase. This means that it is not possible for observed input combinations that do not satisfy all the constraints in the condition part of f'' to cause the activation of its corresponding t-unit.

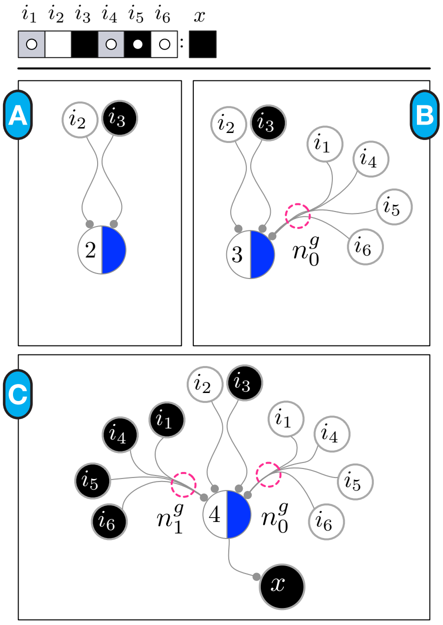

Figure 2: 'Branch and fuse' integration of a group-invariant enput into a single t-unit. (A) Since f'' has $n_\ell = 2$ the corresponding units for the inputs $x_i \in X_\ell$ are linked to the t-unit with single fibres, and τ is incremented from zero to two. (B) shows the integration of the sub-constraint $n_g^0 = 1$. Since in this case $n_g - (n_g^0 - 1) = n_g = 4$ there is only one subset S and hence a single branch from each s-unit for $x_i \in X_g : x_i = 0$ is taken and fused into a single ending. The threshold is increased from two to three. (C) Finally, the integration of the second sub-constraint in g, namely n_g^1 has the same properties as the sub-constraint integrated in (B). The final threshold of the t-unit is therefore $\tau = 4$. Notice that only input-combinations that satisfy all the constraints in Expression (1) can produce the firing activation of the t-unit.
