# Supplementary material for: Canalization and Control in Automata Networks: Body Segmentation in Drosophila melanogaster
Source: PLoS One. 2013 Mar 8;8(3):e55946. doi: 10.1371/journal.pone.0055946 (PMC3592869; doi:10.1371/journal.pone.0055946)
Supplement: Data S4 — Algorithms for the computation of minimal configurations. (PDF) [file pone.0055946.s004.pdf]

4 Minimal Configurations

To obtain a single MC, start with a seed configuration $\boldsymbol{x}$ such that $\sigma(\boldsymbol{x}) \rightsquigarrow \boldsymbol{\mathcal{P}}$ – or with a partially specified configuration $\hat{\boldsymbol{x}} : \sigma(\hat{\boldsymbol{x}}) \rightsquigarrow \boldsymbol{\mathcal{P}}$ not guaranteed to be a MC.

Systematically, assume a randomly picked specified state in the seed to become unknown, checking each time whether the network still unfolds to the target; when it does, the modified configuration becomes the new seed.

This random state selection and de-specification procedure is repeated until there are no more specified states in the seed configuration that can be set to the unknown state and still guarantee dynamic unfolding to the target.

In such case the final seed left is a MC, which is then added to a set $\boldsymbol{X}'$ that contains the MCs that unfold to $\boldsymbol{\mathcal{P}}$ (see Algorithm 3).

Algorithm 3 Compute a single MC from a given *seed* configuration

$\boldsymbol{x}' \leftarrow seed$
$N_e \leftarrow \emptyset$
while $|N_e| \neq o(\boldsymbol{x}')$ **do** {where $o(\boldsymbol{x}')$ is the number of specified nodes in the original seed}
  $\boldsymbol{x}^* \leftarrow \boldsymbol{x}'$
  Randomly select a node x in one of the Boolean states $\{0, 1\}$, such that $x \in \boldsymbol{x}^* \wedge x \notin N_e$
  Assign the unknown state to node x ($x = \#$)
  if $\sigma(\boldsymbol{x}^*) \rightsquigarrow \boldsymbol{\mathcal{P}}$ **then**
    $\boldsymbol{x}' \leftarrow \boldsymbol{x}^*$
  end if
  $N_e \leftarrow N_e \cup \{x\}$
end while
return $\boldsymbol{x}'$

When Algorithm 3 is executed several times starting with the same seed, it is likely to find different MCs.

This is because the algorithm selects nodes that become unknown at random and thus repeating the algorithm enough times will find all the distinct – yet overlapping – MCs that redescribe the seed.

This would allow, for instance, to identify MCs with group invariant enputs.

To control the search for MCs that can be derived from a single seed – while enabling the identification of as many as possible – we use a search tolerance parameter (δ) that specifies how many times to run Algorithm 3 without returning a new MC that is not already a member of $\boldsymbol{X}'$.

Every time a new MC is added to $\boldsymbol{X}'$, the δ counter is reset.

There is no heuristic to choose the optimal value of δ that guarantees that all possible MCs that describe the seed have been found.

Thus the larger its value, the greater the confidence that the complete set of MCs for the seed has been identified.

After the algorithm is run multiple times to either exhaustively search or stochastically sample $\boldsymbol{X}$, we obtain a set of MCs $\boldsymbol{X}'$, all of which unfold to $\boldsymbol{\mathcal{P}}$.

We refer to the function that returns a set of MCs ($\boldsymbol{X}'$) obtained from a *seed* with tolerance δ (using Algorithm 3) as $\gamma(seed, \delta)$.

The set $\boldsymbol{X}'$ can be then redescribed for group-invariance, obtaining the final set of two-symbol MCs $\boldsymbol{X}''$.

However, considering the computational limitations that are often associated with two-symbol redescription of large sets of wildcard schemata, we refer to a set of MCs – in general – simply as $\boldsymbol{X}'$, and to $\boldsymbol{X}''$ only in particular contexts, where the two-symbol redescription of MCs is necessary for some particular type of inference.

The stochastic search algorithm for finding MCs just described takes as input a single seed.

Once the set of MCs for this seed has been identified, it is straightforward to expand the algorithm to find MCs that describe different seed configurations by generating new seeds that (1) unfold to $\boldsymbol{\mathcal{P}}$; (2) are not redescribed by the current set of MCs $\boldsymbol{X}'$ and (3) repeating the algorithm.

We use the algorithm in such an iterative fashion to find, for instance, MCs that characterize a given basin of attraction.

The procedure is simple, see Algorithm 4.

Algorithm 4 Compute the set of MCs starting with a given *seed* configuration (tolerance ρ)

$\sigma(seed) \rightsquigarrow \boldsymbol{\mathcal{P}}$
$\boldsymbol{X}' \leftarrow \gamma(seed, \delta)$
$i \leftarrow 1$
while $i \leq \rho$ do
    Generate a new seed $\boldsymbol{y} : (\boldsymbol{y} \not\mapsto \boldsymbol{X}') \wedge \sigma(\boldsymbol{y}) \rightsquigarrow \boldsymbol{\mathcal{P}}$ { the symbol $\not\mapsto$ means ‘not redescribed by’}
    $\boldsymbol{Y}' \leftarrow \gamma(\boldsymbol{y}, \delta)$
    $\boldsymbol{X}' \leftarrow \boldsymbol{X}' \cup \boldsymbol{Y}'$
    $i \leftarrow i + 1$
end while
return $\boldsymbol{X}'$
