# Supplementary material for: Canalization and Control in Automata Networks: Body Segmentation in Drosophila melanogaster
Source: PLoS One. 2013 Mar 8;8(3):e55946. doi: 10.1371/journal.pone.0055946 (PMC3592869; doi:10.1371/journal.pone.0055946)
Supplement: Data S5 — Further details concerning the minimal configurations found for the segment polarity network model. (PDF) [file pone.0055946.s005.pdf]

5 MCs for the SPN model

To control how many MCs were derived from single seeds we executed Algorithm 3 using a tolerance $\delta = 10^5$ to stop searching for new MCs from a given seed.

Then, using the obtained set of MCs $\boldsymbol{X}'$, we executed Algorithm 4 to find new MCs from randomly generated seed configurations that, while unfolding to the wild-type attractor pattern, are not redescribed by any MC $\boldsymbol{x}' \in \boldsymbol{X}'$.

For every new seed, the resulting set of MCs was added to $\boldsymbol{X}'$ before generating a new seed.

The tolerance parameter to stop the search for new seeds was set to $\rho = 10^6$ and the same tolerance used previously ($\delta = 10^5$) was kept for deriving MCs from each new seed configuration.

The resulting MCs are available in different files as follows:

- `mcc_wt.csv` contains the 1745 wildcard MCs identified overall.
- `mcc_bio.csv` contains the subset of wildcard MCs that redescribe the trajectory between the known initial condition of the SPN and the wild-type attractor.
- `mcc_min.csv` contains the subset of wildcard MCs with the smallest number of enputs (23).
- `mcc_noprot.csv` contains the subset of wildcard MCs that were no protein (except for SLP in cells 3 and 4) can be present and acting as an enput.
- `mcc_twosym.csv` contains the subset of wildcard MCs that were redescribed as two-symbol schemata using $\beta = 8$.
- `mcc_chaves_2nd.csv` contains the subset of 171 wildcard MCs that do not require the second necessary condition proposed by Chaves and Albert (see main text).
- `mcc_minexpressed.csv` contains the subset of wildcard MCs with the lowest number of on enputs.
- `mcc_maxexpressed.csv` contains the subset of wildcard MCs with the largest number of on enputs.

During the stochastic interventions on literal enputs, the states of SLP across the parasegment were disturbed from the fixed-state assumption that determines their state in the SPN model. This means that the STG of the original model, which contains 2^{56} configurations is enlarged to another that contains 2^{60}, where new attractors are possible. We identified one of these when perturbing the state of SLP_3, this attractor is depicted in Figure

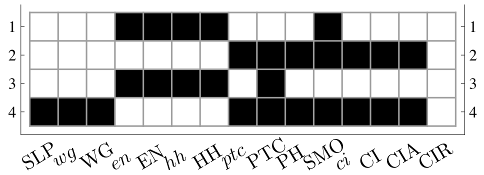

Figure 3: New attractor identified when performing stochastic interventions on SLP_3.
