# Supplementary material for: Canalization and Control in Automata Networks: Body Segmentation in Drosophila melanogaster
Source: PLoS One. 2013 Mar 8;8(3):e55946. doi: 10.1371/journal.pone.0055946 (PMC3592869; doi:10.1371/journal.pone.0055946)
Supplement: Data S6 — Basic notions of the inclusion/exclusion principle. (PDF) [file pone.0055946.s006.pdf]

6 Inclusion/Exclusion Principle

Given a set of MC (two-symbol) schemata X'', the number of configurations it redescribes can be approximated by a lower and an upper bound computed from the equivalent set of wildcard schemata X'. A lower bound is given by:

$$|\underline{X}| = \sum_{i=1}^{|X'|} N(x'_i) - \sum_{i=1}^{|X'|-1} \sum_{j=i+1}^{|X'|} N(x'_i, x'_j) \tag{7}$$

where $N(\{x'\})$ is the number of configurations commonly redescribed by every schema in the set {x'}. This is computed using the number of wildcards in common to every schema:

$$N(x'_i, x'_j, x'_k, ...) = \begin{cases} 0, & \text{if no overlap} \\ 2^{n^{\#}_{i,j,k,\dots}}, & \text{otherwise} \end{cases} \tag{8}$$

where $n_{i,j,k,\dots}$ is the number of wildcards in common for schemata $x'_i, x'_j, x'_k, \dots$. If the schemata in this set are logically consistent, meaning that no variable state in one is the negation of the other, then there is a nonempty intersection (overlap) in the sets of configurations they redescribe. But if even one variable in one schema is the negation of a variable in another schema, there is no overlap[2].

An upper bound for the number of configurations redescribed by MC schemata set X'' is given by:

$$|\overline{X}| = \sum_{i=1}^{|X'|} N(x'_i) - \sum_{i=1}^{|X'|-1} \sum_{j=i+1}^{|X'|} N(x'_i, x'_j) + \sum_{i=1}^{|X'|-2} \sum_{j=i+1}^{|X'|-1} \sum_{k=j+1}^{|X'|} N(x'_i, x'_j, x'_k) \tag{9}$$

The lower bound assumes that the only overlaps that exist are between pairs of schemata; it is a lower bound because it subtracts the pairwise overlaps more than necessary when overlaps exist amongst three or more sets of schemata.

The upper bound adds the overlaps amongst triplets of schemata that were removed from the lower bound, but it adds (or counts) the overlaps amongst four or more schemata more than necessary.

Naturally, there is a simpler, alternative upperbound, which assumes no overlap among the sets of configurations redescribed by every schema:

$$|\overline{X}| = \sum_{i=1}^{|X'|} N(x'_i) \tag{10}$$

The former upper bound (eq. 9) yields a more accurate estimate, which will typically be close to the lower bound.

[2] Naturally, if a variable in schema x'_i is set to a logical state, and is set to wildcard in x'_j, there is no logical inconsistency.
